# Multiplex genomewide association analysis of breast milk fatty acid composition extends the phenotypic association and potential selection of *FADS1* variants to arachidonic acid, a critical infant micronutrient

**DOI:** 10.1136/jmedgenet-2017-105134

**Published:** 2018-03-07

**Authors:** Josyf C Mychaleckyj, Uma Nayak, E Ross Colgate, Dadong Zhang, Tommy Carstensen, Shahnawaz Ahmed, Tahmeed Ahmed, Alexander J Mentzer, Masud Alam, Beth D Kirkpatrick, Rashidul Haque, Abu Syed Golam Faruque, William A Petri Jr

**Affiliations:** 1 Center for Public Health Genomics, Department of Public Health Sciences, University of Virginia, Charlottesville, Virginia, USA; 2 Department of Medicine, Vaccine Testing Center, University of Vermont, College of Medicine, Burlington, Vermont, USA; 3 Center for Public Health Genomics, University of Virginia, Charlottesville, Virginia, USA; 4 Wellcome Trust Sanger Institute, Cambridge, UK; 5 Center for Nutrition and Food Security, International Centre for Diarrhoeal Disease Research, Dhaka, Bangladesh; 6 International Centre for Diarrhoeal Disease Research, Dhaka, Bangladesh; 7 Wellcome Trust Centre for Human Genetics, Oxford, UK; 8 Division of Infectious Diseases and International Health, Department of Medicine, Department of Pathology, University of Virginia, Charlottesville, Virginia, USA

**Keywords:** breast milk, fatty acids, selection, fatty acid desaturase, genomewide association

## Abstract

**Background:**

Breast milk is the sole nutrition source during exclusive breastfeeding, and polyunsaturated fatty acids (FAs) are critical micronutrients in infant physical and cognitive development. There has been no prior genomewide association study of breast milk, hence our objective was to test for genetic association with breast milk FA composition.

**Methods:**

We measured the fractional composition of 26 individual FAs in breast milk samples from three cohorts totalling 1142 Bangladeshi mothers whose infants were genotyped on the Illumina MEGA chip and replicated on a custom Affymetrix 30K SNP array (n=616). Maternal genotypes were imputed using IMPUTE.

**Results:**

After running 33 separate FA fraction phenotypes, we found that SNPs known to be associated with serum FAs in the *FADS1/2/3* region were also associated with breast milk FA composition (experiment-wise significance threshold 4.2×10^−9^). Hypothesis-neutral comparison of the 33 fractions showed that the most significant genetic association at the *FADS1/2/3* locus was with fraction of arachidonic acid (AA) at SNP rs174556, with a very large per major allele effect size of 17% higher breast milk AA level. There was no evidence of independent association at *FADS1/2/3* with any other FA or SNP after conditioning on AA and rs174556. We also found novel significant experiment-wise SNP associations with: polyunsaturated fatty acid (PUFA) 6/PUFA3 ratio (sorting nexin *29*), eicosenoic (intergenic) and capric (component of oligomeric Golgi complex 3) acids; and six additional loci at genomewide significance (<5×10^−8^).

**Conclusions:**

AA is the primary FA in breast milk influenced by genetic variation at the *FADS1/2/3* locus, extending the potential phenotypes under genetic selection to include breast milk composition, thereby possibly affecting infant growth or cognition. Breast milk FA composition is influenced by maternal genetics in addition to diet and body composition.

## Introduction

Long-chain polyunsaturated fatty acids (LCPUFAs) are important for growth and cognitive development during early life since they are structural components of membrane phospholipids, precursors of inflammation-mediating eicosanoids, and also modulate gene expression by acting as agonists or ligands for transcription factors.^1^ In postnatal exclusively breastfeeding infants, breast milk is the sole source of these compounds and other essential FAs. Since the first enzyme in the omega-6 and omega-3 pathway conversion of precursor essential linoleic (LA, C18:2n6) and alpha-linolenic (ALA, C18:3n3) FAs, delta-6 desaturase encoded by the FA desaturase 2 gene (*FADS2)*, is likely rate limiting, and LCPUFAs are beta-oxidised or used in developmental processes, endogenous neonatal LCPUFA synthesis is unable to meet total demand and preformed LCPUFA metabolites in breast milk are necessary to prevent depletion.^2–5^ Hence, the composition of breast milk is important in ensuring that the infant obtains the correct balance of macronutrients and micronutrients, particularly the critical arachidonic (AA, 20:4n6) and docosahexaenoic (DHA, 22:6 n−3) acids.^6–8^

Previous candidate gene and genomewide association studies (GWAS) of FA levels have focused on circulating FA levels in blood plasma or erythrocyte membranes in adults^9–16^ with fewer in infants and children^17–20^ and were motivated by hypotheses of lipids as mediators of cardiovascular disease, inflammation and cancer.^15 21–23^ By comparison, there are limited extant data exploring the association of genetic variation with breast milk composition, and previous studies have generally tested a few candidate SNPs in the *FADS1/2/3* region against selected breast milk LCPUFA components.^24–26^

Given the dearth of information about genetic influences on breast milk composition and hence the nutritional supply to the early developing ex utero infant, we undertook a GWAS of genetic variants associated with the percentage composition of 26 FAs in breast milk samples from more than 1100 Bangladeshi mothers, enrolled with their infants in three cohorts in two locations in Bangladesh, from the *Performance of Rotavirus and Oral Polio Vaccines in Developing Countries* (PROVIDE) study,^27^ and a more recent study of Cryptosporidium infection in infants (*Field Studies of Cryptosporidiosis in Bangladesh*, ‘CRYPTO’ study, manuscript in preparation). We hypothesised that GWA analysis could identify genetic variants that are associated with breast milk FA composition, and by comparing the GWA results of the 26 assayed FAs, we could identify the FA components most likely to be under the influence of genetic variation. The study design included infant genotyping only, but the breast milk compositional data prompted us to ask whether we could impute maternal genotypes using the uniparental obligate allelic transmissions and hence perform a GWAS in the mothers accounting for uncertainty in the imputation. Our results suggest that the approach worked well, particularly benefiting from the extraordinary proportion of variance in the FA traits explained by individual SNPs.

## Materials and methods

### Study populations

Study participants were drawn from two separate studies of three birth cohorts conducted in two different locations in Bangladesh: urban Mirpur Thana (in Dhaka) and rural Mirzapur Thana (25 miles North-West of Mirpur). Both populations generally lacked access to treated water, but Mirzapur had reduced household crowding compared with the denser slums of Mirpur.

#### PROVIDE study

The study design and population has been described elsewhere.^27 28^ Seven hundred predominantly slum-dwelling mother–infant dyads, with no known maternal or fetal complications, were enrolled in Mirpur, Dhaka within 7 days post-delivery, into a birth cohort between May 2011 and November 2012.

#### Field studies of Cryptosporidiosis in Bangladesh (CRYPTO) study

Seven hundred and fifty-eight mother–infant pairs were recruited into two cohorts, one in Mirzapur (n=258) and one in Mirpur (n=500), Bangladesh, enrolled within 7 days postdelivery between June 2014 and March 2016.

The protocol and procedures were approved by the Ethical Review Committee for human subjects protection and Research Review Committee for scientific merit at the International Centre for Diarrhoeal Diseases Research, Bangladesh and the Institutional Review Boards at the University of Virginia and University of Vermont. All participants provided written informed consent at study entry.

### Breast milk FA determination

A description of breast milk collection and quantification of a comprehensive panel of FAs to include saturated, monounsaturated and polyunsaturated FAs has been published for the PROVIDE study.^29^ Similar sample collection and processing was adopted in CRYPTO study. A single breast milk sample from 1419 mothers (PROVIDE n=683 and CRYPTO n=736), collected within 6 weeks postpartum using a dried milk spot protocol, was assayed for 26 FAs by gas chromatography at OmegaQuant Analytics Laboratory (Sioux Falls, South Dakota, USA). Individual breast milk FAs were expressed as %wt/wt of total identified FA.^30 31^

### DNA extraction and handling

Blood samples from 700 PROVIDE and 713 CRYPTO infants were drawn in the field clinics and processed using standard laboratory protocols. More details are in online supplementary methods.

10.1136/jmedgenet-2017-105134.supp1 Supplementary file 1

### Custom Affymetrix Axiom 30K SNP ‘MalChip’ Array—PROVIDE study

A custom Affymetrix SNP array was developed during 2013 to investigate the genetic associations of a broad spectrum of infant and maternal phenotypic traits linked to impaired infant growth, development, metabolism, infectious disease susceptibility, enteric and systemic inflammation and cognitive development. PubMed searches identified SNPs described in prior GWAS of related phenotypes. The 11 500 unique SNPs identified were supplemented with tagging SNPs for 162 candidate genes. After designability review, the resulting malnutrition SNP array (dubbed the ‘MalChip’) contained probes for 33 588 SNPs. Four-digit resolution human leucocyte antigen (HLA) genotyping was performed on the 700 PROVIDE infant DNA samples and converted to 159 pseudo-SNPs which were merged into the MalChip genotype file. Six hundred and forty infant DNA samples were genotyped on the MalChip at the Center for Public Health Genomics, University of Virginia, USA on an Affymetrix GeneTitan machine. More details are available in online supplementary methods.

### Illumina GWA genotyping—both studies

Infant DNA samples from the PROVIDE (n=576) and CRYPTO (n=672) studies were also genotyped under the VaccGene consortium programme (*VaccGene: Characterising the Genetic Determinants of Vaccine Response in Children from the Developing World*) on precommercial versions of the Illumina multiethnic genotyping array (MEGA) at the Wellcome Trust Sanger Institute. The second version of the array contained 1 522 034 SNPs and was used for the PROVIDE samples. Subsequent genotyping for the CRYPTO samples was performed on a later array version containing 1 655 469 SNPs.

### Imputation of maternal genotypes

Using obligate Mendelian inheritance and assuming Hardy-Weinberg equilibrium, we calculated the probability that a maternal genotype is either AA, AB or BB conditional on the genotype of the infant. Statistical details are described in online supplementary methods. For SNPs directly typed in the infant, the imputation into mother is the only source of uncertainty, but for untyped, imputed infant SNPs, the maternal genotype is the convolution of the two sources of uncertainty. The dual loss of information means that properly calibrated statistical inference from de novo ranking of maternal association results in imputed SNPs is not possible, so the initial GWAS results were filtered to include only genotyped or perfectly imputed SNPs. A custom script converted the infant genotype data to maternal genotype data in Oxford Statistics format, preserving the maternal imputation uncertainty.

### SNP imputation and GWA analysis

Imputation and GWA analysis of the two studies was performed separately, at different times, as genotyping data became available. The samples in both study data sets were separately phased using SHAPEIT V2.r837^32^ and then imputed using IMPUTE V2.3.2 and Oct2014 Phase3 1000 Genomes panels^33^ (more details in online supplementary methods). SNP association analysis was performed using PLINK1.9 for the MalChip infant genotype data and SNPTEST2.5.2^34^ for the maternal genotype data imputed from the infant MalChip data, without reducing the maternal genotypes to an expected non-integer allele dosage. The score test in SNPTEST incorporates the maternal imputation at typed SNPs analogously to the uncertainty with SNP imputation. Because of the maternal genotype imputation, the initial genomewide scan was restricted to SNPs that were either directly genotyped in the infants or perfectly imputed with info=1 in both PROVIDE and CRYPTO to avoid double loss of information through untyped and maternal imputation. Selected SNPs of interest with info<1 were tested as described.

### Phenotypes and statistical models

The quantitative trait outcomes in these analyses were the 26 FA concentrations, expressed as %wt/wt, bounded in composition range [0,100%], summing to 100% per mother. Seven derived major summary fraction FA measures, computed by simple summation of the individual FAs of each class, were added as phenotypes: SFA (total saturated), PUFA3 (total *cis*-polyunsaturated omega-3), PUFA6 (total *cis*-polyunsaturated, omega-6), PUFA (total PUFA=PUFA6+PUFA3), PUFA6/PUFA3 ratio, MFA (total monounsaturated) and TFA (total trans). All were log transformed to stabilise variance, except PUFA6/PUFA3 (square root). Multiple linear regression models were run within each study using a score test to test the marginal additive association of each SNP with each FA phenotype, and the summary results were meta-analysed using a fixed-effects model using METAL.^35^ The minimal GWAS screening model included adjustments for maternal age, infant age at breast milk sample, infant sex and adjustment for study site (CRYPTO study only). Principal components were not included in the association screening model but were included in robustness tests of selected results (described further in online supplementary methods). Conditional models for AA and for the leading SNP also included log(%AA) or lead SNP as covariates. The compositional FA phenotypes had a range of intercorrelation coefficient magnitudes 0–0.998 resulting in 12.0 effective independent phenotypes calculated from the eigenvalues of the FA correlation matrix using the method of Li,^36^ yielding an experiment-wise Bonferroni-adjusted significance rate of 5×10^−8^/12.0=4.2×10^−9^.

## Results

### Demographic and clinical characteristics of the study populations

**Figure 1:**
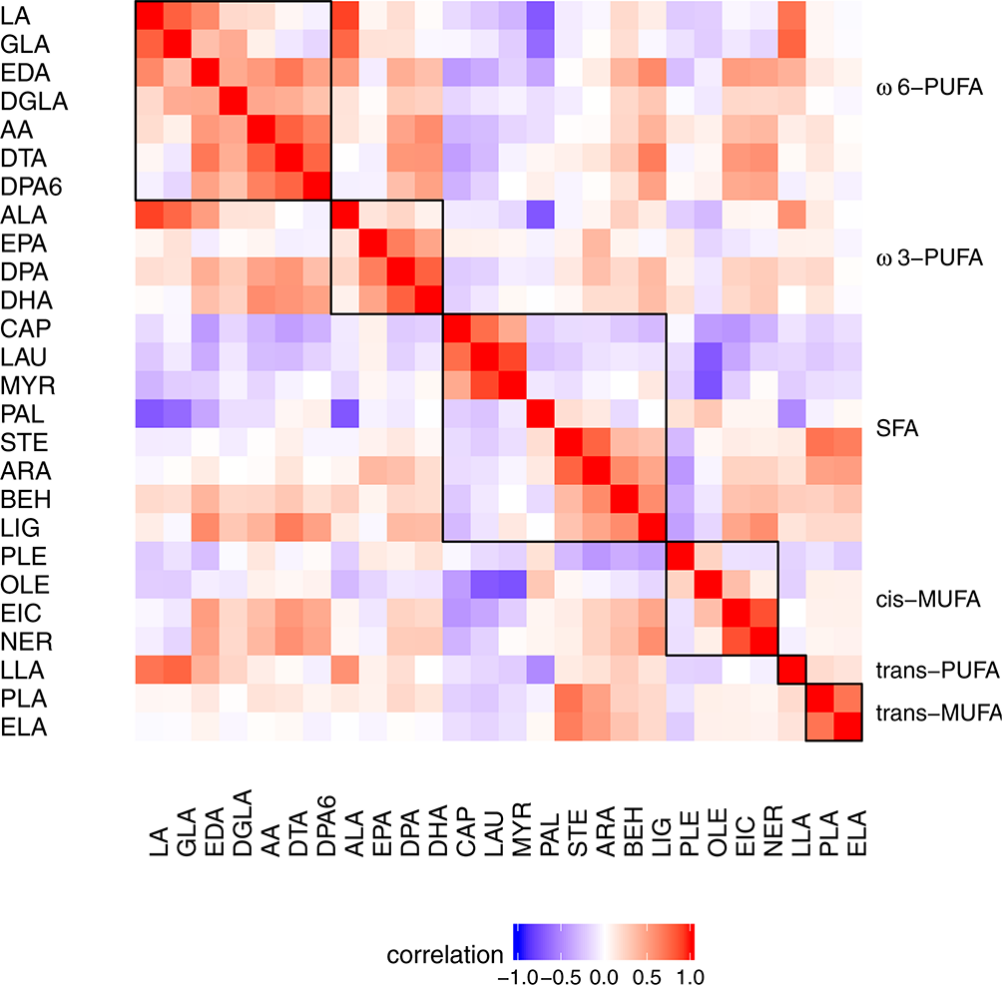
Correlation plot of the 26 assayed FAs ordered into major subfraction categories. Omega-6 and omega-3 PUFA, SFA, *cis*-MUFA, *trans*-PUFA and *trans*-MUFA. The abbreviations for the individual FAs are listed in table 1. The deepest red colour represents the most positively correlated FA compositions (Pearson correlation coefficient =+1.0) and the deepest blue colour, the most anticorrelated (Pearson correlation coefficient=−1.0). FA, fatty acid; MUFA, monounsaturated fatty acid; PUFA, polyunsaturated fatty acid; SFA, saturated fatty acid.

See online supplementary table S1 which summarises the demographic and clinical characteristics of the Bangladeshi families, and the participant/sample flow and loss to follow-up is shown in online supplementary figure S1. After study dropout and sample quality control (QC), n=532 (PROVIDE) and n=402 (CRYPTO) families from Mirpur and n=208 (CRYPTO) from Mirzapur were retained with both breast milk FA concentrations and infant GWA data for analysis. The Affymetrix replication ‘MalChip’ PROVIDE study cohort retained 616 families, because genotyping was conducted prior to the GWAS and total DNA collection from neonatal infants was limited. The breast milk samples were collected at mean days of lactation (infant age) of 5.8–10.9 days with range 3–43 days. A summary of the breast milk FA concentrations is shown in table 1 and the FA correlation structure in figure 1. There were significant differences between the cohorts in the breast milk FA composition, with higher PUFA6 and PUFA3 in the CRYPTO cohorts compared with PROVIDE, with lower SFA/PUFA ratio and higher concentration of AA and DHA.

10.1136/jmedgenet-2017-105134.supp3 Supplementary file 3

10.1136/jmedgenet-2017-105134.supp4 Supplementary file 4

**Table 1:** Breast milk composition in Bangladeshi mothers measured as the percentage of 26 individual FAs, with computed percentage of derived major fractions

FA	Formula	Abbrev*	PROVIDE MalChip n=616	PROVIDE GWAS n=532	CRYPTO Mirpur n=402	CRYPTO Mirzapur n=208
Saturated FA		SFA	48.29 (6.63)	48.16 (6.51)	44.61 (5.95)	43.62 (5.89)
Capric	C10:0	CAP	1.17 (0.52)	1.15 (0.52)	0.78 (0.44)	0.62 (0.4)
Lauric	C12:0	LAU	8.20 (3.01)	8.18 (2.99)	5.85 (2.32)	5.10 (2.41)
Myristic	C14:0	MYR	8.04 (3.06)	8.01 (3.04)	6.40 (2.62)	6.55 (2.81)
Palmitic	C16:0	PAL	26.67 (3.71)	26.64 (3.76)	26.6 (3.63)	26.79 (3.23)
Stearic	C18:0	STE	3.93 (0.82)	3.89 (0.77)	4.67 (0.96)	4.26 (0.78)
Arachidic	C20:0	ARA	0.14 (0.03)	0.14 (0.03)	0.15 (0.04)	0.14 (0.03)
Behenic	C22:0	BEH	0.07 (0.02)	0.07 (0.02)	0.07 (0.02)	0.06 (0.02)
Lignoceric	C24:0	LIG	0.08 (0.03)	0.08 (0.03)	0.08 (0.03)	0.10 (0.05)
Monounsaturated FA†		MUFA	36.69 (5)	36.76 (5.06)	36.45 (4.94)	38.10 (5.03)
Palmitoleic	C16:1n7	PLE	2.94 (1.09)	2.96 (1.1)	2.67 (0.91)	3.76 (1.14)
Oleic	C18:1n9	OLE	33.18 (4.59)	33.24 (4.67)	33.09 (4.53)	33.03 (4.37)
Eicosenoic	C20:1n9	EIC	0.41 (0.16)	0.41 (0.16)	0.50 (0.2)	0.82 (0.47)
Nervonic	C24:1n9	NER	0.15 (0.11)	0.15 (0.11)	0.19 (0.14)	0.48 (0.36)
Polyunsaturated FA-ω6†		PUFA-ω6	13.17 (5.3)	13.24 (5.36)	16.75 (5.45)	15.96 (4.29)
Linoleic	C18:2n6	LA	11.21 (5.03)	11.29 (5.09)	14.36 (5.15)	13.05 (4.08)
γ-Linolenic	C18:3n6	GLA	0.16 (0.11)	0.16 (0.11)	0.21 (0.12)	0.17 (0.1)
Eicosadienoic	C20:2n6	EDA	0.41 (0.17)	0.41 (0.18)	0.54 (0.2)	0.66 (0.2)
Dihomo-γ-linolenic	C20:3n6	DGLA	0.55 (0.17)	0.55 (0.17)	0.64 (0.2)	0.73 (0.22)
Arachidonic	C20:4n6	AA	0.53 (0.15)	0.53 (0.15)	0.65 (0.14)	0.81 (0.17)
Docosatetraenoic‡	C22:4n6	DTA	0.18 (0.1)	0.18 (0.1)	0.21 (0.1)	0.34 (0.18)
Docosapentaenoic-n6	C22:5n6	DPA6	0.12 (0.05)	0.12 (0.05)	0.13 (0.05)	0.2 (0.08)
Polyunsaturated FA-ω3†		PUFA-ω3	1.12 (0.5)	1.12 (0.5)	1.39 (0.59)	1.65 (0.45)
α-Linolenic	C18:3n3	ALA	0.53 (0.4)	0.54 (0.4)	0.78 (0.53)	0.82 (0.37)
Eicosapentaenoic	C20:5n3	EPA	0.06 (0.07)	0.06 (0.07)	0.05 (0.04)	0.05 (0.02)
Docosapentaenoic-n3	C22:5n3	DPA	0.14 (0.08)	0.14 (0.08)	0.15 (0.07)	0.22 (0.09)
Docosahexaenoic	C22:6n3	DHA	0.39 (0.14)	0.39 (0.14)	0.40 (0.14)	0.55 (0.14)
Total polyunsaturated FA†		PUFA	14.29 (5.69)	14.36 (5.75)	18.14 (5.92)	17.61 (4.59)
*Trans-*FA		TFA	0.74 (0.34)	0.73 (0.32)	0.81 (0.32)	0.67 (0.26)
Palmitelaidic	C16:1n7t	PLA	0.06 (0.03)	0.06 (0.03)	0.07 (0.03)	0.08 (0.03)
Elaidic	C18:1 t	ELA	0.36 (0.25)	0.35 (0.24)	0.41 (0.25)	0.32 (0.19)
Linoelaidic	C18:2n6t	LLA	0.32 (0.16)	0.32 (0.16)	0.33 (0.17)	0.27 (0.14)
Ratios						
SFA/PUFA			3.95 (1.69)	3.92 (1.66)	2.81 (1.24)	2.74 (1.21)
PUFA-ω6/PUFA-ω3		PUFA6/3	12.40 (3.25)	12.48 (3.28)	12.84 (3.14)	9.98 (2.15)

All FA compositions are expressed as %wt/wt. Values shown are mean (SD).

The columns show the clinical values for the subset of each total cohort with breast milk FAs measured and post-QC for genetic data, as per table 1.

*Abbrev is the abbreviation for the FA used throughout this manuscript.

†All monounsaturated and polyunsaturated FAs are cis-isomers.

‡Also known as adrenic acid.

CRYPTO, Cryptosporidiosis in Bangladesh; FA, fatty acid; GWAS, genome wide association studies; PROVIDE, Performance of Rotavirus and Oral Polio Vaccines in Developing Countries; QC, quality control.

### Genotyping results

The genotyping QC results are shown in online supplementary table S2, and more detailed discussion is in online supplementary results. For the custom Affymetrix 30K MalChip, post-QC, 626 (/640 total) PROVIDE samples and 20 908 SNPs (/33 588 total) remained. After merging the 159 HLA pseudo-SNPs, 16 688 (/21067=79.2%) were common in this population with minor allele frequency (MAF) >0.05. For the Illumina preproduction MEGA array used, 776 921 SNPs (52.8%) were polymorphic in PROVIDE and 970 928 SNPs (60.5%) in CRYPTO. Filtering the set of SNPs to those with info=1 in both study groups and MAF≥0.05 (SNP passed QC in both studies) or MAF>0.1 (SNP passed QC in only one study), there were approximately 932K SNPs available.

10.1136/jmedgenet-2017-105134.supp2 Supplementary file 2

10.1136/jmedgenet-2017-105134.supp5 Supplementary file 5

### Ancestry of the Bangladeshi population

**Figure 2:**
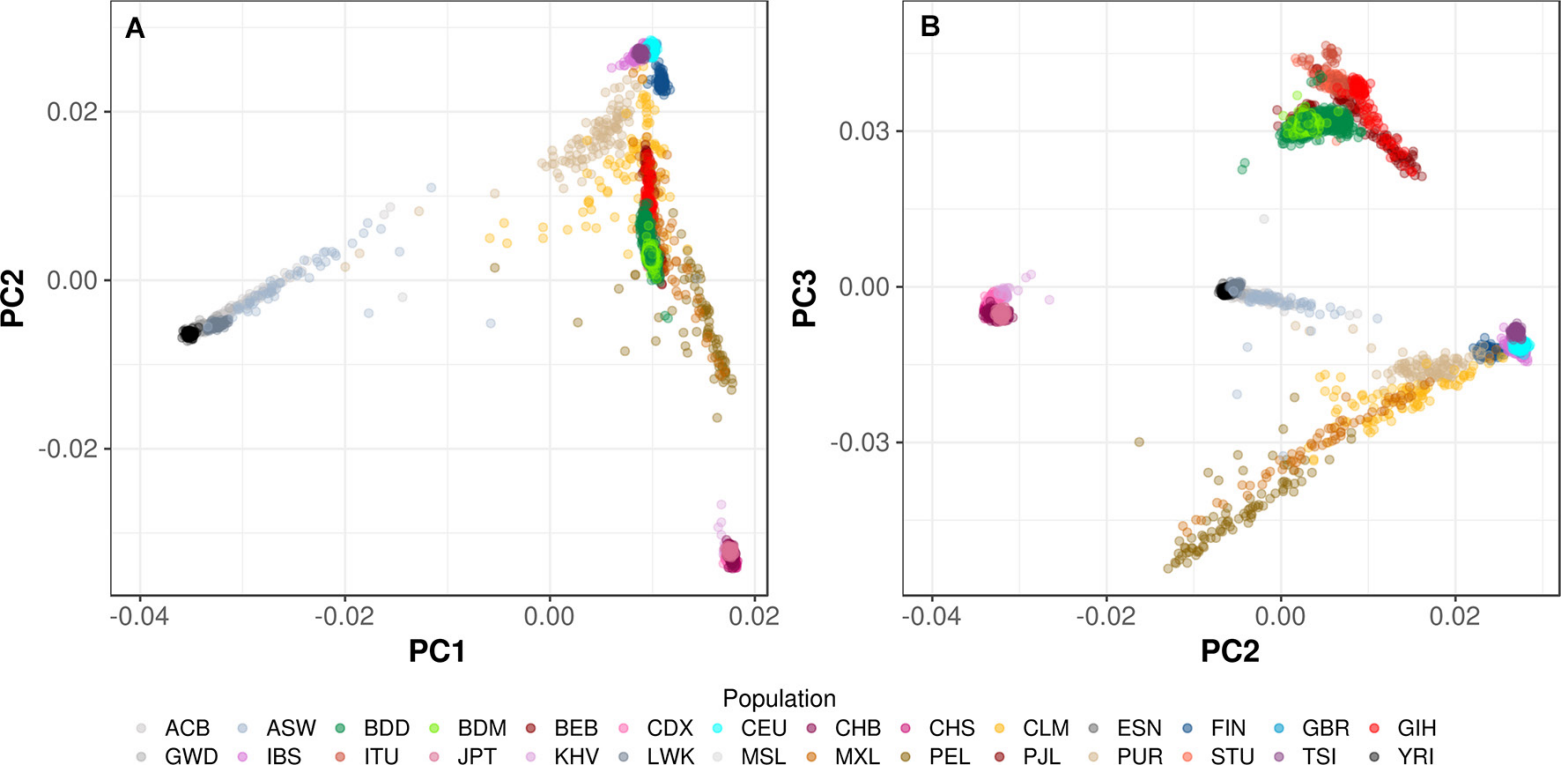
PC plots of PC1×PC2 (A) and PC2×PC3 (B) generated by projecting the Bangladeshi samples enrolled from two sites in this study (dark green glyphs, labelled as BDD: Bangladeshis in Dhaka; light green glyphs, labelled as BDM: Bangladeshis in Mirzapur) onto the axes of variation derived from the 2504 1000 genomes samples (20130502 phase 3 data release). The three-letter population codes in the legend are the usual codes used to refer to 1000 genomes populations and are listed in online supplementary table S3. PC, principal component.

Figure 2 shows the first three principal components of the PROVIDE and CRYPTO study samples, 541+404=945 Bangladeshis in Dhaka (dark green glyphs) and 226 Bangladeshis in Mirzapur (light green glyphs) projected onto components defined using the 1000 genomes populations (online supplementary table S3). The samples lie between the European and South/East Asian clusters and overlapped the 1000 genomes Indian subcontinent samples, particularly Bengalis in Bangladesh. The samples comprise a reasonably tight cluster which explained the low genomic control inflation and are genetically more similar to the European population (60%) than to East Asians (40%), stemming from ancient migration and admixture events.

10.1136/jmedgenet-2017-105134.supp6 Supplementary file 6

### Genomewide scans identified the *FADS1/2/3* region as a locus for breast milk AA and three other FA loci

The GWAS results are shown in table 2 and online supplementary table S4 for 1142 families with complete data, filtered from 932 k frequent SNPs (MAF>0.05 both studies; or MAF>0.1 present in one study) with info=1, that is, SNPs genotyped or perfectly imputed. We found four distinct loci that met the maternal experiment-wise significance threshold of 4.2×10^−9^ and another six that met the less stringent traditional threshold of 5×10^−8^. The chromosome 11 *FADS1/2/3* region was one of the four, but only for breast milk AA fraction, and 24 SNPs met the experiment-wise significance threshold consistent with the known strong LD through this region in Europeans. The *FADS1/2/3* locus results are discussed in more detail below. The top SNP association (rs7198595) was with the ratio of total PUFA6/PUFA3 and was located in intron 18 of the sorting nexin 29 (*SNX29*) gene. The second SNP (rs34440628), associated with eicosenoic acid (EIC, C20:1n9), was found in an intergenic region with no immediately obvious link to function. The fourth locus contained eight SNPs in perfect LD associated with capric acid (CAP, C10:0) levels. The SNP listed in table 2 (rs12583793) was within an intron of component of oligomeric Golgi complex 3 (*COG3*) intron and was closest or within epigenetic marks (H3K4Me1 and H3K27Ac). This SNP was found to be a cis-eQTL for *COG3* transcript in transformed fibroblast cells and tibial artery tissue, *SLC25A30* in transformed fibroblasts and *FAM194B* in subcutaneous adipose (Gene–Tissue Expression Project (GTEx) portal, https://www.gtexportal.org). Six other distinct loci were significant at the usual 5×10^−8^ significance level but not experiment-wise, five of which were located within a gene locus.

10.1136/jmedgenet-2017-105134.supp7 Supplementary file 7

**Table 2:** Association results for all 33 fatty acid phenotypes at common SNPs in the Illumina MEGA genomewide scan, in descending maternal test significance

FA	Gene	n (SNPs)	Lead SNP	Chr	Position	RefA	AltA	RAF	β	SE β	Dir	Mother P value	Infant P value
PUFA6/PUFA3*	***SNX29***	**1/1**	**rs7198595**	**16**	**12564907**	**a**	**g**	**0.07**	**−4.60**	**0.69**	**−−**	**4.5×10^−11^**	**5.1×10^−6^**
**EIC**	***Intergenic***	**1/1**	**rs34440628**	**2**	**222523887**	**a**	**g**	**0.09**	**0.64**	**1.08**	**++**	**1.2×10^−10^**	**9.1×10^−7^**
**AA**†	***FADS1***	**24/25**	**rs174556**	**11**	**61580635**	**t**	**c**	**0.17**	**0.85**	**1.03**	**−−**	**1.5×10^−10^**	**4.7×10^−10^**
**CAP**‡	***COG3***	**8/8**	**rs12583793**§	**13**	**46057286**	**a**	**g**	**0.06**	**0.41**	**1.15**	**−−**	**2.0×10^−10^**	**5.4×10^−6^**
LAU	*SFXN5*	0/1	rs11695051	2	73234432	t	c	0.94	1.84	1.11	++	4.4×10^−9^	1.1×10^−5^
OLE	*ZNF804B*	0/4	rs12535041	7	88573471	a	c	0.93	1.19	1.03	++	5.0×10^−9^	4.5×10^−6^
DPA6	*DIAPH3*	0/1	rs76065946	13	60373704	a	t	0.93	0.69	1.07	−−	5.2×10^−9^	4.9×10^−7^
PAL	*ATP8A2*	0/1	rs7335338	13	26242505	a	t	0.90	1.13	1.02	++	2.1×10^−8^	1.8×10^−6^
CAP	*Intergenic*	0/4	rs6986921	8	138865556	a	g	0.12	0.60	1.10	−−	2.4×10^−8^	3.7×10^−6^
MUFA	*ZNF804B*	0/4	rs12535041	7	88573471	a	c	0.93	1.18	1.03	++	4.9×10^−8^	3.2×10^−5^

SNPs shown are those with: MAF>0.05 (SNP present in both studies) or MAF>0.1 (present in only one study); info=1 in both studies and P value (HW) in infants >0.00001, with P value (association) <5×10^−8^, ranked by descending maternal test significance. RefA is the reference allele and AltA, the alternative allele. β and SE β are the exponentiated values from the log (FA fraction) model in mothers with the exception of the PUFA6/PUFA3. For all phenotypes except PUFA6/PUFA3, β measures the multiplicative change in the fractional composition of the FA per reference allele. All SNPs with annotated genes lie within the gene locus. n (SNPs) is the total number of SNPs significant at 4.2×10^−9^/5×10^−8^ at each locus. Sample size for all tests was 532 (PROVIDE)+610 (CRYPTO)=1142. Those results shown in bold are significant at the genomewide experiment-wise significance threshold of 4.2×10^−9^.

Individual FA abbreviations are also shown in table 1.

*PUFA6/PUFA3 was modelled as √(PUFA6/PUFA3), hence β is change in the ratio per allele at a standardised PUFA6/PUFA3=12.

†Twenty-four SNPs were significant at the experiment-wise threshold for AA, but only the top SNP is shown in this table.

‡All eight CAP SNPs were in perfect LD; the listed SNP lies within H3K27Ac/H3K4Me1 marks.

§From the GTEx portal, rs12583793 is a cis-eQTL for: *COG3* and *SLC25A30* transcripts in transformed fibroblast cells, *COG3* transcript in tibial artery and *FAM194B* (renamed *ERICH6B*) transcript in subcutaneous adipose tissue.

AA, arachidonic acid; CAP, capric acid; *COG3*, component of oligomeric Golgi complex 3; CRYPTO, Cryptosporidiosis in Bangladesh; DPA6, docosapentaenoic-n6; EIC, eicosenoic; FA, fatty acid; GTex, Gene–Tissue Expression Project; LAU, lauric acid; MAF, minor allele frequency; MUFA, monounsaturated fatty acid; OLE, oleic acid; PAL, palmitic acid; PROVIDE, Performance of Rotavirus and Oral Polio Vaccines in Developing Countries; RAF, Reference Allele Frequency; SNX29, sorting nexin 29.

### AA is the only FA unconditionally associated at *FADS1/2/3*

**Figure 3:**
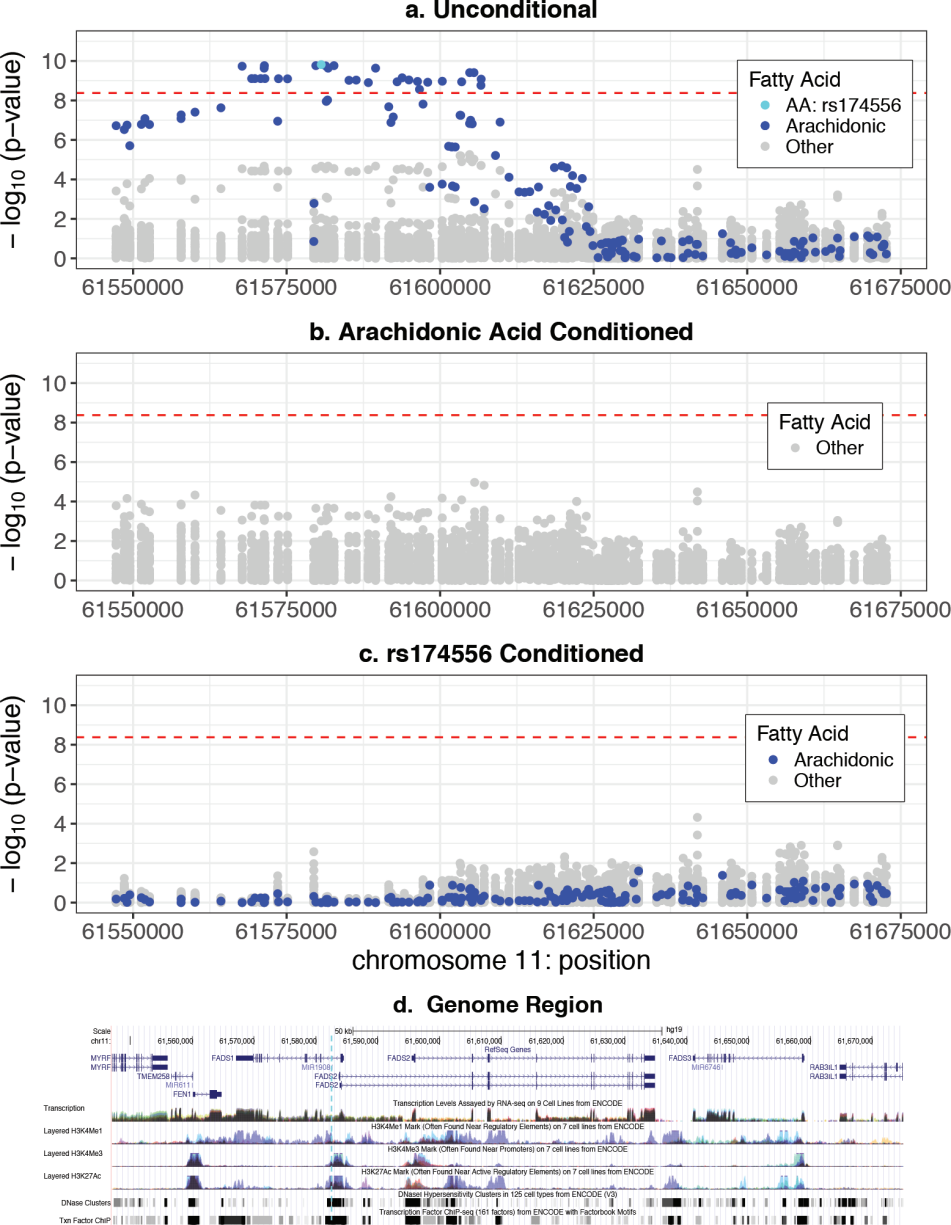
Regional genome association plots for the GWAS results of the *FADS1/2/3* region of chromosome 11. The horizontal red dashed line in each plot shows the −log 10 (experiment-wise significance rate). Panel A shows the unconditioned additive genetic model results for all tested SNPs (85)×all 33 breast milk fatty acid phenotypes in this region plotted as chromosome map position (bp) versus −log(P value) of the score test result from SNPTEST. Arachidonic acid phenotype results are highlighted in blue and the lead SNP in cyan. Panel B shows the same SNPs and genetic model, but with the addition of log(AA) as a covariate adjustment to condition on AA concentration in breast milk (AA phenotype results not shown). Panel C shows the same analyses but with the SNP genetic models conditioned on the lead SNP (rs174556) as a covariate. Panel D shows the hg19 local genome physical map with overlaid epigenetic tracks (UCSC browser). GWAS, genome wide association studies.

We performed a detailed search of the *FADS1/2/3* region using more permissive SNP search filters to capture a wider range of possible FA associations. We defined the region as hg19 chr11:61 547 000–61 673 000 encompassing the two previously identified LD blocks in Europeans.^13 37 38^ Expanding the SNP filter to info>0.9 in both studies and MAF>0.05 yielded 180 SNPs in the region versus only 85 used in the initial GWAS scan. The results are shown graphically in figure 3A, and the detailed top regional SNPs with maternal P value <1×10^−9^ are in table 3 (full *FADS1/2/3* region results are in online supplementary table S5). The clustering of the significant associated SNPs recapitulates the LD structure previously described in Europeans, seen here in the Bangladeshi populations. The top-ranked 58 SNPs were all associated with the AA phenotype, whereas the first non-AA SNP association was rank 59 for DPA6 (docosapentaenoic acid-6, C22:5n6), an omega-6 metabolite of AA after two-carbon elongation and desaturation, P value=5.5×10^−6^. These results suggested that only AA was associated at *FADS1/2/3* in unconditional univariate analyses. We replicated the association at *FADS1/2/3* in the PROVIDE study in the separate Affymetrix Axiom MalChip data set (45 SNPs in this region), online supplementary table S6 and supplementary results.

10.1136/jmedgenet-2017-105134.supp8 Supplementary file 8

10.1136/jmedgenet-2017-105134.supp9 Supplementary file 9

**Table 3:** Association results for all 33 fatty acid phenotypes and SNPs with MAF>0.01 at the chromosome 11 *FADS1/2/3* locus, ordered by descending maternal test significance with P value <1.0×10^−9^

Gene	SNP	Position	RefA	AltA	RAF	β*	SE β*	Maternal P value
*FADS1*	rs174556	61580635	t	c	0.17	0.85	1.025	1.5×10^−10^
*FADS1, MIR1908*	rs174561	61582708	t	c	0.83	1.17	1.025	1.7×10^−10^
*FADS1*	rs174549	61571382	a	g	0.17	0.85	1.025	1.7×10^−10^
*FADS1*	rs174555	61579760	t	c	0.83	1.17	1.025	1.7×10^−10^
*FADS1*	rs174557†	61581368	a	g	0.83	1.17	1.025	1.8×10^−10^
*FADS1*	rs174544†	61567753	a	c	0.17	0.85	1.025	1.8×10^−10^
*FADS2*	rs28456	61589481	a	g	0.83	1.17	1.025	2.3×10^−10^
*FADS1*	rs174560	61581764	t	c	0.83	1.17	1.025	2.3×10^−10^
*FADS1*	rs174548	61571348	c	g	0.83	1.17	1.025	2.3×10^−10^
*FADS2*	rs174578	61605499	a	t	0.19	0.86	1.025	3.9×10^−10^
*FADS2*	rs174577	61604814	a	c	0.19	0.86	1.025	3.9×10^−10^
*FADS2*	rs174568	61593816	t	c	0.18	0.86	1.025	7.1×10^−10^
*FADS1*	rs174550	61571478	t	c	0.82	1.16	1.025	7.8×10^−10^
*FADS1*	rs174546	61569830	t	c	0.18	0.86	1.025	7.8×10^−10^
*FADS1*	rs174545	61569306	c	g	0.82	1.16	1.025	7.8×10^−10^
*FADS1*	rs174547	61570783	t	c	0.82	1.16	1.025	7.8×10^−10^
*FADS1*	rs174551	61573684	t	c	0.82	1.16	1.025	7.9×10^−10^
*FADS1*	rs174553†	61575158	a	g	0.82	1.16	1.025	8.0×10^−10^
*FADS2*	rs174581	61606683	a	g	0.19	0.87	1.025	8.3×10^−10^
*FADS2*	rs774882452†	61594920	ct	c	0.82	1.16	1.025	9.2×10^−10^
*FADS2*	rs35473591	61586328	ct	c	0.18	0.86	1.025	9.3×10^−10^
*FADS2*	rs174562	61585144	a	g	0.82	1.16	1.025	9.5×10^−10^

The 22 highest ranked SNPs were all for log(AA) phenotype, but only those with P<1×10^−9^ are shown (22 in total).

All meta-analysis effect directions were consistent between the two studies for all SNPs with maternal P value <0.018.

*Exponentiated values from the log (FA fraction) model in mothers and hence the multiplicative change in the fractional composition of the FA per reference allele.

†SNPs were imputed with an information content >0.99 and <1; all others had info=1.

AA, arachidonic acid; FA, fatty acid; FADS2, fatty acid desaturase 2.

### No evidence for common variant association with other, non-AA FAs at *FADS1/2/3*

The results in table 3 and its extended version suggested that AA was the only FA associated with common variants in the *FADS1/2/3* region at experiment-wise or genomewide significance, but the AA association could have masked additional underlying independent FA associations. We repeated the *FADS1/2/3* regional analysis for the remaining 32 phenotypes with log(AA) fraction included as a conditioning covariate in the log-additive linear models. The results are shown in figure 3B. We found no evidence of an independent secondary common variant association with another FA since none of the FA-conditioned SNP tests reached experiment-wise or genomewide (5×10^−8^) significance, and the minimum P value was 1.1×10^−5^, for total PUFA3 derived phenotype. The DPA6 association (was rank 59) diminished to P value=0.0057, suggesting that intra-pathway correlation with AA accounted for much of the association and ranking.

### No evidence for a second independent common SNP association at *FADS1/2/3* with any FA fraction

We reran the *FADS1/2/3* analysis for all 33 phenotypes but conditioning on the lead SNP for AA, rs174556, in the log-additive linear models. The results are shown in figure 3C. We found no statistical evidence of a second independently associated locus for any of the 33 phenotypes at experiment-wise, genomewide or lesser (1×10^−5^) significance. There was no evidence of a secondary associated common variant locus for AA.

### rs174556 and the next eight SNPs are the leading *FADS1/2/3* SNPs associated with breast milk AA fraction

Our lead SNP, rs174556, lies within intron 2 of the *FADS1* gene, less than 100 bp 3′ to exon 2, within a region of H3K4Me1 and H3K27Ac marks. *FADS1* codes for the omega-6 (and omega-3) pathway enzyme with delta-5 desaturase activity that converts DGLA (20:3n6) to AA (20:4n6) and also catalyses distal down-pathway steps.^39^ The next eight SNPs ranked by P value after the top SNP had identical effect sizes and r^2^≥0.98 with rs174556, hence there was insufficient statistical evidence to choose the most likely causative among them by association. We estimated that the major allele of rs174556 led to a 17% increase in fractional AA content per allele and explained 9.6% of the variation in log(AA) (table 3), an extraordinary genetic effect size, but comparable to results seen previously for association of lipid traits in this region. rs174556 was in strong LD with rs174546 which was recently identified as a candidate selected SNP in a GWA of 230 ancient Eurasian genomes.^40^ SNP rs174556 was one of the 28 SNPs in high LD that discriminated derived and ancestral European haplotypes spanning 38.9 kb from *FADS1* through *FADS2.*^37^

### Literature-based replication from prior studies of selected SNPs with AA concentration

Despite extensive prior literature on the association of FADS region genetic variants with adult lipid and FA traits in blood plasma and erythrocyte membranes, we found only four candidate SNP papers testing the association of FADS genetic variation with breast milk FA composition, listed in online supplementary table S7. In all cases, AA was the most significantly associated omega-6 FA, all P values were <0.005 and all effect directions were entirely consistent with our results. In the Lattka study,^41^ there was no association with any of the five omega-3 FAs after correction for multiple testing. In the Morales study,^26^ the only other comparable P value to AA was for DHA at rs174602 (beta=−12%, P value=0.0006), but the SNP maternal genotyping rate was less than 95%.

10.1136/jmedgenet-2017-105134.supp10 Supplementary file 10

### rs174556 is a cis-eQTL for *FADS1* and *FADS2* gene expression

Searches of the Gene–Tissue Expression Project (GTEx) portal for significant cis-eQTLs at rs174556 (https://www.gtexportal.org, v6 data release) showed that it is locus for multiple gene transcripts in multiple tissues, including both *FADS1* and *FADS2*. Whole blood was the tissue with the most significant cis-eQTL for rs174556 in GTEx (by more than 10 orders of magnitude and one of the largest effect sizes), but 24 other tissues were significant (online supplementary table S8).

10.1136/jmedgenet-2017-105134.supp11 Supplementary file 11

## Discussion

We have conducted the first GWAS of breast milk composition using a comprehensive panel of 26 individual and 7 derived FA compositions to give tests across 33 correlated compositional phenotypes, which we ranked by SNP and FA in a hypothesis-neutral approach. With results from only 1142 families, we were able to establish experiment-wise significant results for a cluster of SNPs in the chromosome 11 *FADS1/2/3* region and that AA was the primary FA fraction in breast milk influenced by maternal genetic variation at this locus, specifically, non-coding SNP rs1746556 localised within the *FADS1* gene. A second analysis on a separate array platform recapitulated the signal with only 616 families. For breast milk AA composition, our results suggest that there is only a single independent common variant association signal at *FADS1/2/3*, and that the nine top SNPs, in near-perfect LD r^2^=0.98–1.0, are the primary candidates at this locus. This result was consistent with, and replicated, four previous *FADS1/2/3* candidate SNP breast milk composition studies, but our results emphasise that the primary breast milk fatty acid component under genetic influence is AA. Using recent GTEx cis-eQTL results, we showed that despite being physically located within the *FADS1* gene, the lead SNP rs1746556 was associated with increased *FADS2* and/or decreased *FADS1* gene expression in various tissues, and because of the very high r^2^ among these nine SNPs, the same eQTL results broadly apply to them as a cluster. Finally, we identified three other new significant loci for specific breast milk FA fractions, and six others that met the traditional GWAS significance threshold but not the adjusted multiplex phenotype testing threshold hence were considered suggestive. Of additional interest is the fact that this is one of few GWAS performed using samples and data from Bangladeshi populations.

This is the first GWAS of breast milk composition, and compared with other lipid traits and phenotypes, breast milk has been the subject of little previous genetic work. Genetic analysis of breast milk composition has lagged behind other plasma lipid traits because breast milk composition is not a standard diagnostic or risk factor entered into medical records and requires more complex and costly chromatographic analysis of samples from women during limited periods of lactation. The importance of the work is that the variants may modify infant growth and cognitive development during breastfeeding,^25^ particularly in comparing breast-fed versus formula-fed infants.^26 42^

We found three new loci in our multiplex GWAS scan, and there are prior published functional data that suggest a link to lipid metabolism. rs7198595, in an intron of *SNX29*, was associated with the ratio of total PUFA6/PUFA3. Fox found that an intronic SNP in this gene (rs1641895) was associated with subcutaneous (P value=0.003) and visceral (P value=0.01) adipose tissues in women^43^ although our lead *SNX29* SNP is not in LD with rs1641895. In a porcine model of mammary gland development during late gestation, *SNX29* was one of 68 genes that was upregulated more than twofold in the near-maturity gland (4 days prepartum) versus immature gland (>30 days prepartum).^44^ Also, in a GWAS of 13 bovine udder traits, SNPs in the coding region of *SNX29* were associated with rear udder height.^45^ The fourth most significant SNP (rs12583793) was associated with CAP (C10:0) and is located in an exon of *COG3.* Besides its energy content, CAP has bactericidal and antifungal properties and may help to protect the infant from infection from pathogens such as *Escherichia coli* and *Candida albicans* on mucosal and skin surfaces.^46–48^

Our association analyses with breast milk employed a relatively small sample by modern genetic association standards, only 1142 for Illumina GWAS analysis and 616 for our custom MalChip. But even using testing that properly allowed for the imputed loss of information in mothers from infant-genotyped SNPs (SNPTEST), we were still able to declare statistical significance at a more stringent alpha level than 5×10^−8^. This was due to the extraordinarily large effects on lipid concentrations and metabolites resulting from variation in the *FADS* genes, seen not just in this study, but also previously.^9 13^ The other new significant loci also have large per SNP variance explained but need to be validated in replication cohorts to adjust the winner’s curse inflation of effect sizes.

Previous work has shown that present-day humans have two common and distinct *FADS1/2* haplotypes consisting of 28 SNPs that are associated with levels of synthesis of LCPUFA, and several of the top nine SNPs associated with breast milk AA in the *FADS1/2/3* region were present in these haplotypes.^37^ There is evidence of positive selection of the haplotype that enhances the ability to produce AA and DHA in African and European populations.^38 49^ Our results for breast milk AA content extend the possible lipid-based phenotypes that may have been subject to ancient selection pressure, especially since AA levels directly influence growth and immune function at the very earliest ages, and AA is a critical structural component of brain and neuronal tissue. This could have had important consequences for individual fitness and survival through adulthood.

Our study has limitations. Absent maternal genotyping data we had to impute the maternal genotypes which led to a loss of power. It is highly likely that there are additional GWAS-significant loci for breast milk FA fractions that we would have been able to identify with maternal genotyping data. Breast milk composition adapts as the infant develops over the first few months, but we collected only a single specimen per mother so were not able to test the effect of genetic variation on longitudinal composition changes, and our results represent averaged association over colostrum, transitional and mature milk stages. Our sample size was limited, hence we were only able to identify FA fractions with relatively large variance explained by genetic variation. There are undoubtedly other loci with smaller effects waiting to be detected. Our study was conducted in cohorts where food insecurity and nutrition may be suboptimal for the infant, which may affect reproducibility of the findings in higher income cohorts, although the population mean %AA in both the PROVIDE and the CRYPTO cohorts was higher than published mean values from a global meta-analysis of 84 studies and was higher than several included studies from high-income countries.^50^ Finally, we used publicly available cis-eQTL data that were generated from adult tissue specimens not derived from lactating women.

In summary, we have performed the first GWAS on breast milk composition, identified FA fractions influenced by genetic variation and made some important observations about the effect of genetic variation at the critical *FADS1/2/3* locus. Our results confirm the idea that breast milk composition is influenced by maternal genetics as well as diet and body composition and extends the list of phenotypes possibly under selection at the *FADS1/2/3* locus.
