# Supplementary material for: Multiplex genomewide association analysis of breast milk fatty acid composition extends the phenotypic association and potential selection of FADS1 variants to arachidonic acid, a critical infant micronutrient
Source: J Med Genet. 2018 Mar 7;55(7):459–68. doi: 10.1136/jmedgenet-2017-105134 (PMC6047159; doi:10.1136/jmedgenet-2017-105134)
Supplement: Supplementary file 1 [file jmedgenet-2017-105134supp001.pdf]

SUPPLEMENTARY METHODS

DNA Extraction and Handling

Blood samples from 700 PROVIDE and 713 CRYPTO infants were drawn in the field clinics, and then transported to the ICDDR,B laboratory (Dhaka, Bangladesh) in insulated carriers with cold packs at 4degC. Approximately 200µL of whole blood was processed for DNA isolation using Qiagen DNeasy blood DNA extraction kit and stored at -20degC in labeled screw-cap tubes and shipped to the Center for Public Health Genomics, Genome Sciences Laboratory at University of Virginia for Affymetrix MalChip genotyping; or to Wellcome Trust Sanger Institute Genotyping Core Lab for Illumina GWAS genotyping. DNA purity was tested on a NanoDrop Spectrophotometer (260/280 absorbance ratio of 1.8). DNA sample tubes were stored at -20degC until genotyping.

HLA genotyping using next generation sequencing (Roche 454) – PROVIDE Study

Four-digit resolution HLA genotyping was performed on the 700 PROVIDE infant DNA samples at the Children's Hospital Oakland Research Institute (CHORI, Oakland, California), using multiplex bar-coded exon-specific PCR amplification and sequenced on the Roche 454 GS Junior System. The class II loci, HLA-DRB1, -DQA1, and -DQB1, were genotyped for exon 2 only, while the class I loci, HLA-A and -B, were genotyped for exons 2 and 3. The methods have been previously described.[1] The Conexio Genomics HLA ASSIGN ATF genotyping software was used to analyze the sequence files to derive HLA genotype calls.[2 3] Each distinct HLA allele was dichotomized into a pseudo-SNP, resulting in genotypes for 159 pseudo-SNPs and merged into the MalChip genotype file.

Custom Affymetrix Axiom 30K SNP 'MalChip' Array – PROVIDE Study

During May 2013, PubMed searches were run to identify SNPs described in prior GWAS studies of related phenotypes using key words "GWAS" OR "genome-wide association" OR "genetic association" AND either: 1) "growth phenotype" (where growth phenotype was one of: "anthropometry", "height", "BMI", "obesity"); 2) "infectious disease" or "pathogen" (where disease or pathogen one of the following: malaria, celiac, crohn's, IBD, rotavirus, cryptosporidium, HIV)"; 3) "micronutrient" (micronutrient was one of: iron, vitamin A, vitamin D, zinc)"; 4) "inflammation"; 5) "diabetes"; 6) "lipid phenotype" (where lipid phenotype was one of: dyslipidemia, fatty acid, PUFA, breast milk). This resulted in a list of approx. 11,500 unique SNPs which was supplemented with SNPs that tagged European and African common variation in 162 candidate genes. This overall list was forwarded to Affymetrix to check for probe coverage and designability and finally resulted in a SNP array that we called the Malnutrition Chip ('MalChip' for short), containing probes for targeted genotyping of approximately 33,588 SNPs. SNPs in the *FADS1/2/3* region were included on the MalChip based on published adult FA and lipid GWAS results prior to May 2013.

Affymetrix Axiom Genotyping and QC – PROVIDE Study

Six hundred and forty (640) infant DNA samples were genotyped on the MalChip at the Genomic Sciences Laboratory of the Center for Public Health Genomics, University of Virginia, Virginia, USA on an Affymetrix GeneTitan machine, and the resulting CEL files were processed using the standard vendor Axiom array quality control pipeline which checked the cluster

separation (Dish QC), sample and SNP call rates, and evidence of structural variation or allelic drop out at each SNP (SNP Polish R script, vendor supplied). The samples were then checked for sex misclassification and KING[4] was used to detect first or second-degree relationships. HLA Pseudo-SNPs were merged into the post-QC MalChip data set using PLINK 1.90.[5]

Imputation of maternal genotypes

From obligate Mendelian inheritance, assuming equal probability of diploid allele transmission and Hardy-Weinberg equilibrium, the probability that a maternal genotype G_m is 0, 1, 2 (AA, AB, BB) conditional on the genotype of the infant G_i is:

$$P = \begin{pmatrix} \Pr(G_m = 0,1,2 \mid G_i = 0) \\ \Pr(G_m = 0,1,2 \mid G_i = 1) \\ \Pr(G_m = 0,1,2 \mid G_i = 2) \end{pmatrix} = \begin{pmatrix} p & q & 0 \\ p/2 & 1/2 & q/2 \\ 0 & p & q \end{pmatrix} \quad (1)$$

where $p = 1 - q$ is the frequency of allele A for the SNP. Hence each known infant genotype results in a (1x3) probability vector $G_{m,j}$ for the jth maternal genotype (SNP subscript omitted for clarity). At an untyped SNP imputed using the infant genotype data, the jth infant genotypes were imputed with uncertainty and probabilities $W_j^T = (w_{0,j}, w_{1,j}, w_{2,j})$, hence the maternal genotype probabilities will be the convolution of the two sources of uncertainty:

$$\begin{pmatrix} \Pr(G_{m,j} = 0) \\ \Pr(G_{m,j} = 1) \\ \Pr(G_{m,j} = 2) \end{pmatrix} = P^T . W_j \quad (2)$$

with renormalization of total genotype probability to unity. The loss of information through these two sources means that properly-calibrated statistical inference from de novo ranking of maternal association results in imputed SNPs is not possible, so the ranked GWAS summary results were

filtered to include only genotyped or perfectly imputed SNPs. Relaxation of the perfect imputation requirement for specific locus analysis is described in the main text. A custom script converted the infant genotype data in Oxford Statistics ‘GEN’ format to the corresponding maternal genotype data in the same format, preserving the maternal imputation uncertainty.

SNP Imputation and GWAS Analysis

The PROVIDE MalChip and GWAS post-QC data sets were merged using PLINK 1.9 to check SNP genotype calls for SNPs common to both platforms, then the sample set was reduced back to the 541 with full GWAS data post-QC. Duplicate SNPs (2,742) and Illumina Indel SNPs (I/D alleles, 127) were dropped prior to phasing. The samples in both study data sets were separately phased using SHAPEIT v2.r837 with options:

‘--burn 10 --prune 10 --main 50 --states 300’

and then imputed using IMPUTE v2.3.2 and the Oct 2014 Phase 3 1000 Genomes panel with options:

‘-buffer 300 -Ne 20000 -k 120 -k_hap 800 -impute_excluded’.

After initial filtering imputed SNPs on the information score (info>0.3), there remained approximately 14M SNPs.

Phenotypes and statistical models

The breast milk composition data contained known key PUFA FAs in both the omega-6 (7 FAs) and omega-3 (4 FAs) pathways, as well as 8 SFA components, 4 monounsaturated, and 3 trans FAs, summing to 1.0 (100%) for each mother, also shown in main text Table 2. Since many were skewed, and Box-Cox and Akaike Information Criterion analysis of the null genetic model residuals confirmed that the log transformed variable resulted in a significantly better fit to the minimal screening model, the FA composition percentage was log transformed for all 26 FAs in the statistical models. All derived FA measures were also log transformed with the exception of PUFA6/PUFA3 ratio, which was found to be better stabilized by a square root (SQRT) transformation.

Missing sample data could be due to cohort drop-out, insufficient breast milk or infant DNA collected, poor infant DNA quality, or genotyping failure. The breast milk samples were collected before infant DNA at different study time points and hence the drop-out for the DNA collection was greater than breast milk sample. We did not attempt to model the drop-out process or DNA/genotyping failure mechanism and ignored the missing data. This is tantamount to assuming that the missing data was missing completely at random or was missing conditional on study variables that were independent of the fatty acid composition outcomes. While it is possible to conceive of mechanisms where the missing data process might be missing not at random, demonstrating an MNAR process is difficult in any study and requires a valid biological/clinical/socioeconomic model for the underlying processes. Post-hoc, since one of the top GWAS results was in the *FADS1/2/3* region with a very plausible fatty acid (AA) this is a positive control that gives confidence that the process is finding likely true positives, but does not indicate possible biases at the other positive loci.

The primary GWAS screening model for each fatty acid contained minimal adjustments as is standard in GWAS studies. This is based on the idea that a person's genotype is selected at random at conception, and should be uncorrelated with confounders. Confounding variables that are added may block latent causative pathways of genetic association, potentially reducing (and biasing) the magnitude of the effect size, although this depends on the outcome and the target population at risk. We included maternal age, infant age at breast milk sample, infant sex, and study site (CRYPTO) as the adjustments. The Bangladeshi samples formed quite tight clusters in our PCA plot and post-hoc, we found the genomic control inflation factors in the QQ Plots to be small. Therefore, we did not include PCs as adjustments (see below).

We used the Illumina MEGA genome-wide association test results to determine the possible effect of substructure and maternal imputation on the genome-wide tests of significance. We conservatively filtered SNP summary statistics on imputation information score (info) = 1, MAF > 0.05, and p-value for exact test of HWE > $1x10^{-5}$. From a quantile analysis of expected null vs observed p-values (QQ-analysis) for all 33 GWAS scans, we found that the genomic control lambda values of the 33 phenotypes ranged from 1.11 (max) to 0.98 (min), Supplementary Table S4. Of these 28/33 had lambda ≤1.05, and 7/33 < 1.0. Absent evidence of systematic, even moderate inflation we did not include principal components (PCs) in the association screening model since inclusion of PCs reduce power at the test SNP unless each PC specifically excludes the SNP or proximal contaminating SNPs in LD with the test SNP. Of the phenotypes that yielded significant SNPs in Table 3 either experiment-wise, or genome-wide, the QQ inflation factors were 0.98 (PAL); 0.99 (CAP, EIC); 1.0 (DPA6); 1.01 (AA, LAU); 1.03 (OLE, MUFA); 1.11 (PUFA6/PUFA3). We reran the single SNP association for SQRT(PUFA6/PUFA3) at rs7198595 including the first two principal components in the minimal screening model in both

studies. We found that the association became more significant and p-value $4.5x10^{-11}$ became $4.4x10^{-12}$.

SUPPLEMENTARY REFERENCES

1. Erlich HA, Valdes AM, McDevitt SL, et al. Next generation sequencing reveals the association of DRB3*02:02 with type 1 diabetes. *Diabetes* 2013;62(7):2618-22. doi: 10.2337/db12-1387

2. Bentley G, Higuchi R, Hoglund B, et al. High-resolution, high-throughput HLA genotyping by next-generation sequencing. *Tissue Antigens* 2009;74(5):393-403. doi: 10.1111/j.1399-0039.2009.01345.x

3. Holcomb CL, Hoglund B, Anderson MW, et al. A multi-site study using high-resolution HLA genotyping by next generation sequencing. *Tissue Antigens* 2011;77(3):206-17. doi: 10.1111/j.1399-0039.2010.01606.x

4. Manichaikul A, Mychaleckyj JC, Rich SS, et al. Robust relationship inference in genome-wide association studies. *Bioinformatics* 2010;26(22):2867-73. doi: 10.1093/bioinformatics/btq559

5. Purcell S, Neale B, Todd-Brown K, et al. PLINK: a tool set for whole-genome association and population-based linkage analyses. American Journal of Human Genetics 2007;81(3):559-75.
