# Supplementary figures and images for: Multiplex genomewide association analysis of breast milk fatty acid composition extends the phenotypic association and potential selection of FADS1 variants to arachidonic acid, a critical infant micronutrient
Source: J Med Genet. 2018 Mar 7;55(7):459–68. doi: 10.1136/jmedgenet-2017-105134 (PMC6047159; doi:10.1136/jmedgenet-2017-105134)

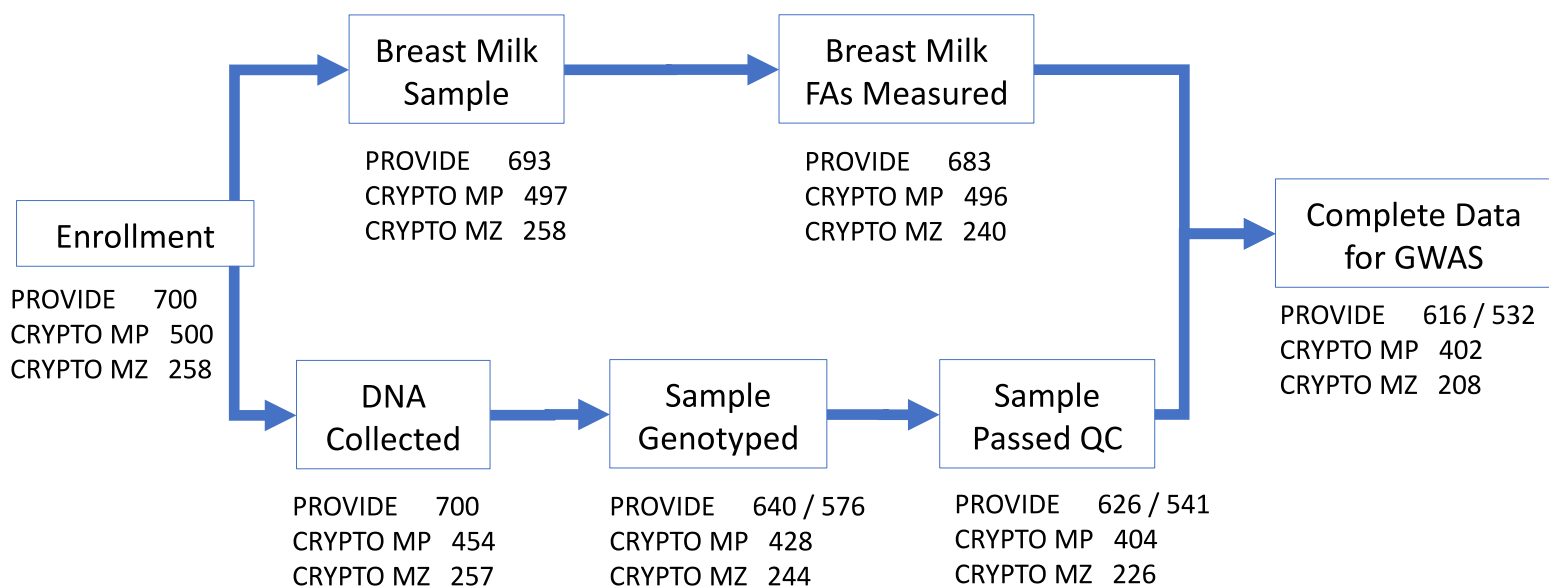

Legend CRYPTO MP: Mirpur site, Crypto Burden Study; CRYPTO MZ: Mirzapur site, Crypto Burden Study

Supplement: Supplementary file 3 [file jmedgenet-2017-105134supp003.pdf]
