# Supplementary material for: Multiplex genomewide association analysis of breast milk fatty acid composition extends the phenotypic association and potential selection of FADS1 variants to arachidonic acid, a critical infant micronutrient
Source: J Med Genet. 2018 Mar 7;55(7):459–68. doi: 10.1136/jmedgenet-2017-105134 (PMC6047159; doi:10.1136/jmedgenet-2017-105134)
Supplement: Supplementary file 4 [file jmedgenet-2017-105134supp004.pdf]

Supplementary Table S2

Results of the genotyping quality control for the MalChip and the Illumina Mega2 array.

	PROVIDE MalChip	PROVIDE GWAS	CRYPTO Mirpur GWAS	CRYPTO Mirzapur GWAS
**Samples Genotyped**	640	576	428	244
**Genotyping Array**	Affymetrix Axiom Custom	Illumina MEGA [a]	Illumina MEGA [a]	Illumina MEGA [a]
**Genotyping QC [b]**				
Dropped DQC < 0.82	0	N/A	N/A	N/A
Dropped Call Rate (< 98% or < 97%)	-12	-25	-16 [c]	
Dropped heterozygosity; mismatch sex	-2	-10	-26 [c]	
Dropped related up to degree 2	0	0	0 [d]	0 [d]
**Samples Post-Genotyping QC**	626	541	404	226
**SNP QC [e]**				
Total SNPs	33,588	1,471,759 [f]	1,603,098 [f]	1,603,098 [f]
SNPs dropped	-2,095 [g]	-112,553 [h]	-24,916 [h]	-24,916 [h]
Monomorphic SNPs	-12,678	-582,277	-607,254	-607,254
**SNPs Remaining**	**20,908**	**776,929**	**970,928**	**970,928**

Quality control for the CRYPTO Study was performed jointly on the samples from the two sites, Mirpur and Mirzapur.

[a] Pre-commercial versions of the Illumina multi-ethnic genotyping array (MEGA). The array version was different for the PROVIDE and CRYPTO Studies, hence the Total SNP counts differed also.

[b] The top half of the table shows the number of DNA samples genotyped by genotyping platform/chip and the number remaining after genotyping QC. Dish QC (DQC) < 0.82 is an Affymetrix Axiom vendor specific QC criterion. Sample call rate was set to 98% for Affymetrix MalChip and 97% for Illumina MEGA 2 array. Samples dropped for outlier heterozygosity; mismatch between inferred sex and study database sex.

[c] Cohort membership was not available to the genotyping lab and analysts who performed this QC.

[d] Since only one second degree relative pair was found within the CRYPTO Study, they were retained leading to a very minor bias.

[e] The bottom half of the table shows the initial total number of SNPs on the two platforms used and the number remaining after QC.

[f] Autosome only. Total inc. X, Y, Mitochondrial, in PROVIDE: 1,522,034; CRYPTO: 1,655,469
[g] Standard Affymetrix Axiom SNP QC was applied using SNPolish R script (Affymetrix) and included 2 control SNPs dropped.

[h] Call Rate <97%
