# Supplementary material for: Multiplex genomewide association analysis of breast milk fatty acid composition extends the phenotypic association and potential selection of FADS1 variants to arachidonic acid, a critical infant micronutrient
Source: J Med Genet. 2018 Mar 7;55(7):459–68. doi: 10.1136/jmedgenet-2017-105134 (PMC6047159; doi:10.1136/jmedgenet-2017-105134)
Supplement: Supplementary file 2 [file jmedgenet-2017-105134supp002.pdf]

SUPPLEMENTARY TABLES

Supplementary Table S1

Demographic and clinical characteristics of the PROVIDE Bangladeshi families.

Characteristic	PROVIDE Mirpur MalChip	PROVIDE Mirpur GWAS	CRYPTO Mirpur GWAS	CRYPTO Mirzapur GWAS
Demographic Area	Urban	Urban	Urban	Rural
Number of Families	616	532	402	208
Mother at Enrollment				
Age, years	24.8 (4.6)	24.9 (4.7)	24.4 (4.4)	23.8 (4.8)
Height, cm	150.4 (5.5)	150.6 (5.5)	149.8 (5.2)	150.7 (5.2)
Postpartum weight, kg	49.4 (9.4)	49.7 (9.5)	51.8 (9.3)	52.6 (8.9)
Postpartum BMI, kg/m^2	21.8 (3.6)	21.9 (3.7)	23.1 (3.9)	23.1 (3.4)
First live birth, n (%)	235 (38.1)	197 (37.0)	156 (38.8)	83 (39.9)
Monthly household income, Taka 000s [a]	13.0 (9.7)	12.9 (9.6)	17.2 (14.7)	20.1 (14.5)
Mother's Education level				
None, n (%)	174 (28.2)	154 (28.9)	86 (21.4)	8 (3.8)
Primary, n (%)	236 (38.3)	201 (37.8)	162 (40.39)	54 (26.0)
Secondary, n (%)	111 (18.0)	99 (18.6)	89 (22.1)	73 (35.1)
Higher, n (%)	95 (15.4)	78 (14.7)	65 (16.2)	73 (35.1)
Infant at Enrollment				
Male, n (%)	325 (52.8)	282 (53.0)	188 (46.8)	116 (55.8)
Age, days	5.0 (1.7)	5.0 (1.7)	3.5 (1.8)	0.5 (0.6)
Weight, kg	2.78 (0.37)	2.77 (0.37)	2.76 (0.38)	2.74 (0.40)

Height, cm	48.7 (1.8)	48.7 (1.7)	48.3 (1.7)	48.7 (2.1)
Length-for-age Z (LAZ)	-0.97 (0.93)	-0.92 (0.88)	-0.95 (0.89)	-0.87 (1.07)
Stunted, LAZ < -2, n (%)	63 (10.2)	53 (10.0)	55 (13.7)	31 (14.9)
Weight-for-age Z (WAZ)	-1.00 (0.89)	-1.30 (0.83)	-1.27 (0.87)	-1.38 (0.94)
Mother Breast Milk Sample				
Days of lactation, days [b]	10.6 (6.4)	10.9 (6.7)	9.5 (6.9)	5.8 (2.5)

Values shown are Mean (SD) for continuous variables or N (%) for dichotomous.

The columns show the clinical values for the subset of the total cohort with breast milk fatty acids measured and post-QC for genetic data.

[a] During the study periods 1 USD = approximately 80 Bangladesh Taka
[b] Days of lactation is equal to the age of the infant in days at breast milk sample time point
