# Supplementary material for: Multiplex genomewide association analysis of breast milk fatty acid composition extends the phenotypic association and potential selection of FADS1 variants to arachidonic acid, a critical infant micronutrient
Source: J Med Genet. 2018 Mar 7;55(7):459–68. doi: 10.1136/jmedgenet-2017-105134 (PMC6047159; doi:10.1136/jmedgenet-2017-105134)
Supplement: Supplementary file 5 [file jmedgenet-2017-105134supp005.pdf]

SUPPLEMENTARY RESULTS

Genotyping Results

The genotyping quality control results are shown in Supplementary Table S1. For the custom Affymetrix Axiom 30K MalChip, after the standard vendor QC process supplemented by tests of sample heterozygosity, mismatch sex, and relatedness up to degree 2, 626 PROVIDE samples remained of the original 640, and 20,908 SNPs of the original 33,588. The great majority of the SNPs dropped (12,680; 86%) were monomorphic since the content was designed to accommodate a second study with African ancestry. After dichotomizing each distinct HLA allele into a pseudo-SNP, we merged an additional 159 pseudo-SNPs into the post-QC MalChip data set giving 21,067 total SNPs. Of these 16,688 (/21067=79.2%) were common in this population with MAF>0.05. The Illumina pre-production MEGA version 2 array used for PROVIDE had 1,471,759 autosomal SNPs that were genotyped resulting in 1,359,206 post-QC (92.4%) and ultimately 776,921 that were also polymorphic (52.8% of the panel). A later version of the array used for the CRYPTO study samples (both sites genotyped and QC processed jointly) contained 1,603,098 autosomal SNPs resulting in 970,928 polymorphic SNPs (60.5%) post-QC. In both PROVIDE and CRYPTO, monomorphism resulted in the largest number of non-usable SNPs (~600K). After limiting the set of SNPs to those with information =1 in both study groups (for maternal GWAS testing, see Methods) and minor allele frequency (MAF) ≥ 0.05 (SNP passed QC and filters in both studies) or MAF>0.1 (SNP passed QC in only one study), there were approximately 932K SNPs available.

Arachidonic Acid is the only FA unconditionally associated at FADS1/2/3 – MalChip Replication

We confirmed the experiment-wise AA association in the *FADS1* gene with SNPs typed on the MalChip (rs174546, rs174547, rs174550). The top-ranked SNP rs174556 was not included in MalChip. These top-ranked MalChip SNPs ranked 14, 16, and 13 respectively in the Illumina pre-production MEGA v2 GWAS results in Table 2 and were in strong LD with the lead GWAS SNP.
