# Supplementary material for: Multiplex genomewide association analysis of breast milk fatty acid composition extends the phenotypic association and potential selection of FADS1 variants to arachidonic acid, a critical infant micronutrient
Source: J Med Genet. 2018 Mar 7;55(7):459–68. doi: 10.1136/jmedgenet-2017-105134 (PMC6047159; doi:10.1136/jmedgenet-2017-105134)
Supplement: Supplementary file 6 [file jmedgenet-2017-105134supp006.pdf]

Supplementary Table S3.
1000 Genomes Populations in the 2013/05/02 Release

**Population**	**Country of Origin**	**Code**
East Asian		
Chinese Dai in Xishuangbanna	China	CDX
Han Chinese in Bejing	China	CHB
Japanese in Tokyo	Japan	JPT
Kinh in Ho Chi Minh City	Vietnam	KHV
Southern Han Chinese	China	CHS
South Asian		
Bengali in Bangladesh	Bangladesh	BEB
Gujarati Indian in Houston, TX	USA	GIH
Indian Telugu in the UK	UK	ITU
Punjabi in Lahore, Pakistan	Pakistan	PJL
Sri Lankan Tamil in the UK	UK	STU
African		
African Ancestry in Southwest US	USA	ASW
African Caribbean in Barbados	Barbados	ACB
Esan in Nigeria	Nigeria	ESN
Gambian in Western Division	The Gambia	GWD
Luhya in Webuye	Kenya	LWK
Mende in Sierra Leone	Sierra Leone	MSL
Yoruba in Ibadan	Nigeria	YRI
European		
British in England and Scotland	UK	GBR
Finnish in Finland	Finland	FIN
Iberian populations in Spain	Spain	IBS
Toscani in Italy	Italy	TSI
Utah residents with Northern and Western European ancestry	USA	CEU
Americas		
Colombian in Medellin	Colombia	CLM
Mexican Ancestry in Los Angeles	USA	MXL
Peruvian in Lima	Peru	PEL
Puerto Rican in Puerto Rico	Puerto Rico	PUR
