# Supplementary material for: Multiplex genomewide association analysis of breast milk fatty acid composition extends the phenotypic association and potential selection of FADS1 variants to arachidonic acid, a critical infant micronutrient
Source: J Med Genet. 2018 Mar 7;55(7):459–68. doi: 10.1136/jmedgenet-2017-105134 (PMC6047159; doi:10.1136/jmedgenet-2017-105134)
Supplement: Supplementary file 7 [file jmedgenet-2017-105134supp007.pdf]

Supplementary Table S4.

Genomic control (Lambda) results for all 33 fatty acid (FA) phenotypes from genome-wide association meta-analysis of genotyped SNPs with MAF>0.05. All phenotypes were log transformed, except PUFA6/PUFA3 which was square root transformed. Fatty acid names corresponding to the abbreviations are available in main text Table 1.

FA	N(SNPs)	Lambda
AA	939115	1.01
ALA	938927	1.00
ARA	939472	1.01
BEH	939341	0.99
CAP	939705	0.99
DGLA	939213	1.01
DHA	939104	1.10
DPA	939265	1.07
DPA6	939047	1.00
DTA	939203	1.00
EDA	939019	1.00
EIC	940351	0.99
ELA	939022	1.04
EPA	939761	1.08
GLA	939026	1.00
LA	939011	1.00
LAU	939371	1.01
LIG	939116	1.00
LLA	939515	1.00
MYR	939009	1.02
NER	939336	1.00
OLE	938998	1.03
PAL	939160	0.98
PLA	939201	1.02
PLE	939020	1.06
STE	940018	0.99
PUFA6	939011	0.99
PUFA3	939020	1.02
PUFA	939013	0.99
TFA	938989	1.02
MUFA	939048	1.03
SFA	939093	1.00
PUFA6/PUFA3	939317	1.11
