# Supplementary material for: Multiplex genomewide association analysis of breast milk fatty acid composition extends the phenotypic association and potential selection of FADS1 variants to arachidonic acid, a critical infant micronutrient
Source: J Med Genet. 2018 Mar 7;55(7):459–68. doi: 10.1136/jmedgenet-2017-105134 (PMC6047159; doi:10.1136/jmedgenet-2017-105134)
Supplement: Supplementary file 8 [file jmedgenet-2017-105134supp008.pdf]

Supplementary Table S5.

Full results at the FADS1/2/3 locus for all FA x SNP combinations with p-value <0.1. Effect size and standard errors have NOT been transformed from the log() or sqroot() transformed phenotypes. Further details are in the legend below the table.

FA	Marker Name	rs#	Pos	Min Info	In Prov	In Cryp	A1	A2	Min Freq	Max Freq	Effect	StdErr	P-value	Dir
AA	11:61580635-C-T	rs174556	61580635	1	1	1	t	c	0.1697	0.1711	-0.1603	0.025	1.53E-10	--
AA	11:61582708-T-C	rs174561	61582708	1	1	1	t	c	0.827	0.8288	0.1584	0.0248	1.72E-10	++
AA	rs174549	rs174549	61571382	1	1	1	a	g	0.1712	0.173	-0.1584	0.0248	1.73E-10	--
AA	rs174555:61579760:T:C	rs174555	61579760	1	1	1	t	c	0.827	0.8288	0.1584	0.0248	1.73E-10	++
AA	rs174557:61581368:A:G	rs174557	61581368	0.999	1	1	a	g	0.827	0.8285	0.1581	0.0248	1.77E-10	++
AA	rs174544:61567753:C:A	rs174544	61567753	0.997	1	1	a	c	0.1712	0.1719	-0.1588	0.0249	1.84E-10	--
AA	rs28456:61589481:A:G	rs28456	61589481	1	1	1	a	g	0.827	0.8278	0.1569	0.0247	2.30E-10	++
AA	11:61581764-T-C	rs174560	61581764	1	1	1	t	c	0.827	0.8278	0.1569	0.0247	2.30E-10	++
AA	rs174548	rs174548	61571348	1	1	1	c	g	0.827	0.8278	0.1569	0.0247	2.30E-10	++
AA	11:61605499-T-A	rs174578	61605499	1	1	1	a	t	0.1844	0.192	-0.1475	0.0236	3.89E-10	--
AA	rs174577	rs174577	61604814	1	1	1	a	c	0.1844	0.192	-0.1475	0.0236	3.89E-10	--
AA	11:61593816-C-T	rs174568	61593816	1	1	1	t	c	0.1826	0.1827	-0.1498	0.0243	7.09E-10	--
AA	rs174550	rs174550	61571478	1	1	1	t	c	0.8154	0.8156	0.148	0.0241	7.80E-10	++
AA	rs174546	rs174546	61569830	1	1	1	t	c	0.1844	0.1846	-0.148	0.0241	7.80E-10	--
AA	11:61569306-C-G	rs174545	61569306	1	1	1	c	g	0.8154	0.8156	0.148	0.0241	7.80E-10	++
AA	rs174547	rs174547	61570783	1	1	1	t	c	0.8154	0.8156	0.148	0.0241	7.80E-10	++
AA	rs174551:61573684:T:C	rs174551	61573684	1	1	1	t	c	0.8153	0.8156	0.148	0.0241	7.93E-10	++
AA	rs174553:61575158:A:G	rs174553	61575158	0.999	1	1	a	g	0.8153	0.8156	0.1479	0.0241	7.99E-10	++
AA	11:61606683-G-A	rs174581	61606683	1	1	1	a	g	0.1866	0.1948	-0.1436	0.0234	8.32E-10	--
AA	rs3834458:61594920:CT:C	rs774882452	61594920	0.996	1	1	ct	c	0.8157	0.8165	0.1481	0.0242	9.19E-10	++
AA	rs35473591:61586328:C:CT	rs35473591	61586328	1	1	1	ct	c	0.1844	0.1854	-0.1473	0.0241	9.28E-10	--
AA	11:61585144-A-G	rs174562	61585144	1	1	1	a	g	0.8145	0.8156	0.1472	0.0241	9.47E-10	++

AA	rs174574	rs174574	61600342	1	1	1	a	c	0.1844	0.1874	-0.1454	0.0238	1.05E-09	--
AA	rs5792235:61596322:CA:C	-	61596322	0.998	1	1	ca	c	0.8131	0.8156	0.1459	0.0239	1.10E-09	++
AA	rs174567:61593005:A:G	rs174567	61593005	0.998	1	1	a	g	0.8151	0.8152	0.1461	0.024	1.14E-09	++
AA	rs174576	rs174576	61603510	1	1	1	a	c	0.1866	0.1976	-0.1431	0.0235	1.14E-09	--
AA	rs1535	rs1535	61597972	1	1	1	a	g	0.811	0.8156	0.1451	0.0239	1.20E-09	++
AA	rs174564:61588305:A:G	rs174564	61588305	1	1	1	a	g	0.8146	0.8152	0.1458	0.024	1.24E-09	++
AA	11:61606642-A-G	rs174580	61606642	1	1	1	a	g	0.8005	0.8108	0.1406	0.0233	1.72E-09	++
AA	rs99780	rs99780	61596633	1	1	1	t	c	0.1848	0.1885	-0.1432	0.0241	2.75E-09	--
AA	rs174559:61581656:G:A	rs174559	61581656	0.999	1	1	a	g	0.1566	0.1611	-0.1478	0.0258	9.52E-09	--
AA	rs7394579:61581450:A:G	rs7394579	61581450	0.984	1	1	a	g	0.8415	0.8467	0.1498	0.0262	1.12E-08	++
AA	rs174570	rs174570	61597212	1	1	1	t	c	0.1279	0.1324	-0.1622	0.0287	1.54E-08	--
AA	rs174565:61591636:C:G	rs174565	61591636	0.997	1	1	c	g	0.8668	0.8706	0.1594	0.0284	2.10E-08	++
AA	rs4246215	rs4246215	61564299	0.995	1	1	t	g	0.1899	0.1976	-0.1312	0.0235	2.36E-08	--
AA	rs174538	rs174538	61560081	0.998	1	1	a	g	0.1683	0.1741	-0.1368	0.0249	3.92E-08	--
AA	11:61557826-T-C	rs102274	61557826	1	1	1	t	c	0.7982	0.8035	0.1304	0.024	5.41E-08	++
AA	rs2727270:61603237:C:T	rs2727270	61603237	0.998	1	1	t	c	0.0765	0.1012	-0.172	0.0317	5.64E-08	--
AA	11:61603358-A-T	rs2727271	61603358	1	1	1	a	t	0.8983	0.9235	0.172	0.0317	5.68E-08	++
AA	11:61592362-A-G	rs174566	61592362	1	1	1	a	g	0.7774	0.7868	0.1203	0.0223	6.85E-08	++
AA	rs102275	rs102275	61557803	1	1	1	t	c	0.7974	0.803	0.1281	0.0239	8.30E-08	++
AA	rs174536	rs174536	61551927	1	1	1	a	c	0.7916	0.7994	0.1266	0.0236	8.31E-08	++
AA	11:61604967-C-T	rs2072113	61604967	1	1	1	t	c	0.0757	0.1017	-0.1679	0.0315	1.01E-07	--
AA	rs4564341:61573540:C:T	rs4564341	61573540	0.935	1	1	t	c	0.0606	0.0673	-0.2238	0.0422	1.13E-07	--
AA	rs174583	rs174583	61609750	1	1	1	t	c	0.2006	0.2089	-0.1193	0.0226	1.26E-07	--
AA	rs57668028:61591995:GAA:G	-	61591995	0.982	1	1	g	gaa	0.163	0.1686	-0.1338	0.0253	1.30E-07	--
AA	11:61604782-A-T	rs2524299	61604782	1	1	1	a	t	0.8992	0.9235	0.1655	0.0315	1.47E-07	++
AA	rs2072114	rs2072114	61605215	1	1	1	a	g	0.8992	0.9235	0.1653	0.0315	1.53E-07	++
AA	rs174535	rs174535	61551356	1	1	1	t	c	0.7936	0.803	0.1252	0.0239	1.64E-07	++
AA	rs174537	rs174537	61552680	1	1	1	t	g	0.194	0.2002	-0.1252	0.0239	1.67E-07	--
AA	rs174533:61549025:G:A	rs174533	61549025	0.986	1	1	a	g	0.1988	0.2053	-0.1247	0.0239	1.76E-07	--

AA	rs108499:61547237:C:T	rs108499	61547237	0.985	1	1	t	c	0.1734	0.1744	-0.131	0.0251	1.90E-07	--
AA	11:61548559-A-G	rs509360	61548559	1	1	1	a	g	0.7226	0.736	0.1077	0.021	3.00E-07	++
AA	11:61549458-A-G	rs174534	61549458	1	1	1	a	g	0.813	0.8152	0.1161	0.0244	1.97E-06	++
AA	rs2524296:61601378:C:T	rs2524296	61601378	0.997	1	1	t	c	0.0634	0.0773	-0.1739	0.0367	2.10E-06	--
AA	rs2845574:61601872:C:T	rs2845574	61601872	0.997	1	1	t	c	0.0634	0.0774	-0.1733	0.0366	2.24E-06	--
AA	rs2845573:61601908:A:G	rs2845573	61601908	0.997	1	1	a	g	0.9226	0.9366	0.1732	0.0366	2.26E-06	++
AA	rs138766446:61602458:CCCA:C	rs765564188	61602458	0.992	1	1	cc ca	c	0.9218	0.9365	0.1726	0.0365	2.29E-06	++
DPA6	11:61604782-A-T	rs2524299	61604782	1	1	1	a	t	0.8992	0.9235	0.2403	0.0529	5.53E-06	++
AA	rs2727265:61609004:C:A	rs2727265	61609004	0.989	1	1	a	c	0.0635	0.0764	-0.1649	0.0365	6.17E-06	--
DPA6	rs2727270:61603237:C:T	rs2727270	61603237	0.998	1	1	t	c	0.0765	0.1012	-0.2379	0.0527	6.32E-06	--
DPA6	11:61603358-A-T	rs2727271	61603358	1	1	1	a	t	0.8983	0.9235	0.2375	0.0527	6.49E-06	++
DPA6	11:61604967-C-T	rs2072113	61604967	1	1	1	t	c	0.0757	0.1017	-0.2336	0.0527	9.26E-06	--
DPA6	11:61605499-T-A	rs174578	61605499	1	1	1	a	t	0.1844	0.192	-0.166	0.0375	9.35E-06	--
DPA6	rs174577	rs174577	61604814	1	1	1	a	c	0.1844	0.192	-0.166	0.0375	9.35E-06	--
DPA6	rs174576	rs174576	61603510	1	1	1	a	c	0.1866	0.1976	-0.1617	0.037	1.27E-05	--
DPA6	rs2072114	rs2072114	61605215	1	1	1	a	g	0.8992	0.9235	0.2307	0.0529	1.30E-05	++
DPA6	11:61606683-G-A	rs174581	61606683	1	1	1	a	g	0.1866	0.1948	-0.1592	0.0373	1.96E-05	--
DPA6	11:61606642-A-G	rs174580	61606642	1	1	1	a	g	0.8005	0.8108	0.1573	0.0369	1.98E-05	++
AA	rs174594:61619829:C:A	rs174594	61619829	0.997	1	1	a	c	0.7473	0.7596	0.0895	0.021	2.08E-05	++
DPA6	rs28456:61589481:A:G	rs28456	61589481	1	1	1	a	g	0.827	0.8278	0.1673	0.0393	2.11E-05	++
DPA6	11:61581764-T-C	rs174560	61581764	1	1	1	t	c	0.827	0.8278	0.1673	0.0393	2.11E-05	++
DPA6	rs174548	rs174548	61571348	1	1	1	c	g	0.827	0.8278	0.1673	0.0393	2.11E-05	++
DPA6	11:61580635-C-T	rs174556	61580635	1	1	1	t	c	0.1697	0.1711	-0.1679	0.0395	2.15E-05	--
DPA6	rs99780	rs99780	61596633	1	1	1	t	c	0.1848	0.1885	-0.1604	0.0378	2.22E-05	--
DPA6	rs174574	rs174574	61600342	1	1	1	a	c	0.1844	0.1874	-0.1592	0.0376	2.29E-05	--
AA	11:61618608-A-G	rs174592	61618608	1	1	1	a	g	0.7478	0.7589	0.0886	0.021	2.53E-05	++
AA	rs143352979:61620628:AAACAAC:A	-	61620628	0.934	1	1	a	aaac aac	0.2703	0.2915	-0.0875	0.0208	2.59E-05	--
DPA6	11:61582708-T-C	rs174561	61582708	1	1	1	t	c	0.827	0.8288	0.1647	0.0393	2.83E-05	++

DPA6	rs174549	rs174549	61571382	1	1	1	a	g	0.1712	0.173	-0.1647	0.0393	2.84E-05	--
DPA6	rs174555:61579760:T:C	rs174555	61579760	1	1	1	t	c	0.827	0.8288	0.1647	0.0393	2.84E-05	++
DPA6	rs174544:61567753:C:A	rs174544	61567753	0.997	1	1	a	c	0.1712	0.1719	-0.1652	0.0395	2.87E-05	--
DPA6	rs174557:61581368:A:G	rs174557	61581368	0.999	1	1	a	g	0.827	0.8285	0.1644	0.0393	2.89E-05	++
DPA6	11:61593816-C-T	rs174568	61593816	1	1	1	t	c	0.1826	0.1827	-0.1607	0.0384	2.90E-05	--
SQRAT63	rs57535397:61641889:G:A	rs57535397	61641889	0.911	1	1	a	g	0.0607	0.0716	-0.3564	0.0856	3.13E-05	--
DPA6	rs174567:61593005:A:G	rs174567	61593005	0.998	1	1	a	g	0.8151	0.8152	0.1583	0.038	3.15E-05	++
DPA6	rs174564:61588305:A:G	rs174564	61588305	1	1	1	a	g	0.8146	0.8152	0.158	0.038	3.24E-05	++
DPA6	rs5792235:61596322:CA:C	-	61596322	0.998	1	1	ca	c	0.8131	0.8156	0.1576	0.038	3.41E-05	++
DPA6	rs3834458:61594920:CT:C	rs774882452	61594920	0.996	1	1	ct	c	0.8157	0.8165	0.1588	0.0384	3.50E-05	++
DPA6	rs174583	rs174583	61609750	1	1	1	t	c	0.2006	0.2089	-0.149	0.036	3.50E-05	--
DPA6	rs174550	rs174550	61571478	1	1	1	t	c	0.8154	0.8156	0.1575	0.0382	3.77E-05	++
DPA6	rs174546	rs174546	61569830	1	1	1	t	c	0.1844	0.1846	-0.1575	0.0382	3.77E-05	--
DPA6	11:61569306-C-G	rs174545	61569306	1	1	1	c	g	0.8154	0.8156	0.1575	0.0382	3.77E-05	++
DPA6	rs174547	rs174547	61570783	1	1	1	t	c	0.8154	0.8156	0.1575	0.0382	3.77E-05	++
DPA6	rs174551:61573684:T:C	rs174551	61573684	1	1	1	t	c	0.8153	0.8156	0.1575	0.0382	3.79E-05	++
DPA6	rs174553:61575158:A:G	rs174553	61575158	0.999	1	1	a	g	0.8153	0.8156	0.1574	0.0382	3.80E-05	++
DPA6	rs35473591:61586328:C:CT	rs35473591	61586328	1	1	1	ct	c	0.1844	0.1854	-0.1569	0.0382	3.95E-05	--
DPA6	11:61585144-A-G	rs174562	61585144	1	1	1	a	g	0.8145	0.8156	0.1568	0.0382	4.01E-05	++
DPA6	rs1535	rs1535	61597972	1	1	1	a	g	0.811	0.8156	0.1547	0.0379	4.43E-05	++
AA	11:61621556-G-C	rs174599	61621556	0.998	1	1	c	g	0.2431	0.2595	-0.0832	0.0208	6.45E-05	--
AA	rs2526680:61611216:C:T	rs2526680	61611216	0.969	1	1	t	c	0.0679	0.0804	-0.1372	0.0347	7.75E-05	--
DPA6	rs174537	rs174537	61552680	1	1	1	t	g	0.194	0.2002	-0.1503	0.0384	8.98E-05	--
AA	rs174601	rs174601	61623140	1	1	1	t	c	0.2455	0.265	-0.0811	0.0207	9.03E-05	--
DPA6	rs102275	rs102275	61557803	1	1	1	t	c	0.7974	0.803	0.1514	0.0387	9.14E-05	++
DPA6	11:61592362-A-G	rs174566	61592362	1	1	1	a	g	0.7774	0.7868	0.1371	0.0354	0.0001052	++
DPA6	11:61557826-T-C	rs102274	61557826	1	1	1	t	c	0.7982	0.8035	0.1503	0.0389	0.0001114	++
DPA6	rs174536	rs174536	61551927	1	1	1	a	c	0.7916	0.7994	0.1454	0.0383	0.0001443	++
DPA6	rs174533:61549025:G:A	rs174533	61549025	0.986	1	1	a	g	0.1988	0.2053	-0.1459	0.0387	0.0001655	--

AA	11:61600327-G-A	rs174573	61600327	1	1	1	a	g	0.1115	0.12	-0.1112	0.0296	0.0001737	--
AA	rs174575	rs174575	61602003	1	1	1	c	g	0.8792	0.8885	0.1093	0.0295	0.0002127	++
DHA	rs57535397:61641889:G:A	rs57535397	61641889	0.911	1	1	a	g	0.0607	0.0716	0.1916	0.0518	0.0002154	++
AA	11:61621194-G-A	rs174598	61621194	1	1	1	a	g	0.2131	0.2167	-0.0814	0.0221	0.000228	--
AA	rs149597144:61602460:CA:C	rs199985711	61602460	0.978	1	1	ca	c	0.8802	0.8863	0.1078	0.0294	0.0002442	++
AA	rs2851682	rs2851682	61616012	0.983	1	1	a	g	0.9162	0.9209	0.1182	0.0322	0.0002449	++
DPA6	rs174570	rs174570	61597212	1	1	1	t	c	0.1279	0.1324	-0.1585	0.0433	0.0002469	--
AA	rs174572	rs174572	61598288	1	1	1	t	c	0.1115	0.1208	-0.1086	0.0297	0.0002512	--
DPA6	rs4564341:61573540:C:T	rs4564341	61573540	0.935	1	1	t	c	0.0606	0.0673	-0.2496	0.0683	0.0002551	--
AA	11:61622227-T-C	rs174600	61622227	0.996	1	1	t	c	0.7814	0.7847	0.0797	0.022	0.000289	++
DPA6	rs4246215	rs4246215	61564299	0.995	1	1	t	g	0.1899	0.1976	-0.1374	0.0379	0.0002942	--
DPA6	rs174535	rs174535	61551356	1	1	1	t	c	0.7936	0.803	0.1403	0.0388	0.000303	++
DPA6	rs174565:61591636:C:G	rs174565	61591636	0.997	1	1	c	g	0.8668	0.8706	0.1548	0.0431	0.0003313	++
DPA6	rs108499:61547237:C:T	rs108499	61547237	0.985	1	1	t	c	0.1734	0.1744	-0.1439	0.0405	0.0003854	--
AA	rs2845572:61614595:G:A	rs2845572	61614595	0.98	1	1	a	g	0.0783	0.084	-0.1133	0.0321	0.0004178	--
AA	rs174587:61612830:C:T	rs174587	61612830	0.961	1	1	t	c	0.0976	0.1113	-0.1143	0.0325	0.0004308	--
AA	rs93923:61613745:C:T	rs93923	61613745	0.961	1	1	t	c	0.0971	0.1119	-0.1138	0.0324	0.0004521	--
DPA6	rs174594:61619829:C:A	rs174594	61619829	0.997	1	1	a	c	0.7473	0.7596	0.1164	0.0332	0.0004553	++
DPA6	11:61618608-A-G	rs174592	61618608	1	1	1	a	g	0.7478	0.7589	0.1161	0.0332	0.0004722	++
DPA6	rs2727265:61609004:C:A	rs2727265	61609004	0.989	1	1	a	c	0.0635	0.0764	-0.2089	0.0604	0.0005435	--
DHA	rs76095489:61664711:A:T	rs76095489	61664711	0.907	1	1	a	t	0.9297	0.9365	-0.1693	0.0494	0.0006038	--
DPA6	rs7394579:61581450:A:G	rs7394579	61581450	0.984	1	1	a	g	0.8415	0.8467	0.1376	0.041	0.0007892	++
SQRAT63	rs76095489:61664711:A:T	rs76095489	61664711	0.907	1	1	a	t	0.9297	0.9365	0.2632	0.0787	0.0008307	++
DPA6	11:61621556-G-C	rs174599	61621556	0.998	1	1	c	g	0.2431	0.2595	-0.1092	0.0327	0.0008348	--
DPA6	rs2524296:61601378:C:T	rs2524296	61601378	0.997	1	1	t	c	0.0634	0.0773	-0.1974	0.0593	0.0008686	--
DPA6	rs174559:61581656:G:A	rs174559	61581656	0.999	1	1	a	g	0.1566	0.1611	-0.1336	0.0402	0.0008937	--
DPA6	rs2845574:61601872:C:T	rs2845574	61601872	0.997	1	1	t	c	0.0634	0.0774	-0.1965	0.0592	0.0008999	--
DPA6	rs2845573:61601908:A:G	rs2845573	61601908	0.997	1	1	a	g	0.9226	0.9366	0.1964	0.0592	0.0009031	++

DPA6	rs143352979:61620628:AAACAAC:A	-	61620628	0.934	1	1	a	aaacaac	0.2703	0.2915	-0.1084	0.0327	0.0009128	--
DPA6	rs138766446:61602458:CCCA:C	rs765564188	61602458	0.992	1	1	ccca	c	0.9218	0.9365	0.1943	0.059	0.0009833	++
DPA6	rs174538	rs174538	61560081	0.998	1	1	a	g	0.1683	0.1741	-0.1293	0.0393	0.001011	--
DPA6	11:61548559-A-G	rs509360	61548559	1	1	1	a	g	0.7226	0.736	0.1077	0.0332	0.00119	++
AA	rs174579	rs174579	61605613	0.998	1	1	t	c	0.0912	0.1069	-0.1024	0.032	0.001348	--
SQRAT63	rs72643557:61579427:C:T	rs72643557	61579427	0.936	1	1	t	c	0.0607	0.0734	-0.2562	0.0803	0.001417	--
DPA6	rs57668028:61591995:GAA:G	-	61591995	0.982	1	1	g	gaa	0.163	0.1686	-0.1256	0.0397	0.001567	--
DPA6	rs174601	rs174601	61623140	1	1	1	t	c	0.2455	0.265	-0.1031	0.0327	0.001644	--
AA	11:61579463-A-G	rs174554	61579463	1	1	1	a	g	0.8156	0.8156	0.0935	0.0297	0.001649	?+
STE	rs174465:61658800:C:T	rs174465	61658800	0.98	1	1	t	c	0.6222	0.628	-0.0487	0.0157	0.001888	--
DPA6	rs2526680:61611216:C:T	rs2526680	61611216	0.969	1	1	t	c	0.0679	0.0804	-0.1809	0.0582	0.001892	--
DHA	11:61620274-T-C	rs72643559	61620274	1	1	1	t	c	0.9312	0.9413	-0.1541	0.0498	0.001962	--
AA	rs174591	rs174591	61617676	0.988	1	1	a	t	0.1468	0.1536	-0.082	0.0267	0.002147	--
STE	rs174458:61656995:C:T	rs174458	61656995	1	1	1	t	c	0.6214	0.6259	-0.0474	0.0155	0.002214	--
DPA6	11:61549458-A-G	rs174534	61549458	1	1	1	a	g	0.813	0.8152	0.1196	0.0391	0.002223	++
AA	11:61624181-T-C	rs97384	61624181	1	1	1	t	c	0.2561	0.2744	-0.0606	0.02	0.00245	--
SQRAT63	11:61620274-T-C	rs72643559	61620274	1	1	1	t	c	0.9312	0.9413	0.2478	0.0823	0.002609	++
STE	11:61657110-C-T	rs174460	61657110	1	1	1	t	c	0.6207	0.6262	-0.0466	0.0155	0.002679	--
AA	rs174582	rs174582	61607168	1	1	1	a	g	0.8891	0.9021	0.0924	0.0312	0.003048	++
AA	rs174593	rs174593	61618831	1	1	1	t	c	0.8414	0.852	0.077	0.0264	0.003521	++
STE	rs174463:61657838:G:A	rs174463	61657838	0.988	1	1	a	g	0.6465	0.6513	-0.0455	0.0156	0.003566	--
STE	rs1000778	rs1000778	61655305	1	1	1	a	g	0.3391	0.3483	0.0465	0.016	0.003786	++
STE	rs174461:61657401:A:C	rs174461	61657401	0.993	1	1	a	c	0.3485	0.3535	0.0451	0.0156	0.003876	++
STE	rs174457:61656975:T:C	rs174457	61656975	0.998	1	1	t	c	0.3469	0.3528	0.0448	0.0156	0.004181	++
DPA	rs57535397:61641889:G:A	rs57535397	61641889	0.911	1	1	a	g	0.0607	0.0716	0.2314	0.0808	0.004187	++
STE	rs174459:61657050:C:T	rs174459	61657050	0.998	1	1	t	c	0.6472	0.6527	-0.0448	0.0156	0.004204	--
EDA	rs2526678:61623793:G:A	rs2526678	61623793	0.95	1	1	a	g	0.0868	0.0871	0.138	0.0482	0.004214	++
PLA	rs174448	rs174448	61639573	1	1	1	a	g	0.6442	0.6511	-0.1173	0.041	0.00423	--

STE	rs1295988:61656658:A:C	rs1295988	61656658	0.998	1	1	a	c	0.3467	0.3528	0.0448	0.0157	0.004245	++
STE	rs5792240:61656541:A:AT	rs5792240	61656541	0.998	1	1	a	at	0.3467	0.3528	0.0448	0.0157	0.004272	++
STE	rs36047269:61656254:T:TC	rs36047269	61656254	0.997	1	1	t	tc	0.3466	0.3528	0.0447	0.0157	0.004332	++
STE	rs174456:61656182:C:A	rs174456	61656182	0.997	1	1	a	c	0.6472	0.6535	-0.0447	0.0157	0.004351	--
DHA	rs72643557:61579427:C:T	rs72643557	61579427	0.936	1	1	t	c	0.0607	0.0734	0.1403	0.0493	0.004467	++
STE	rs174464_61657926	rs174464	61657926	0.987	1	1	a	g	0.3487	0.358	0.0447	0.0157	0.004471	++
AA	rs174589:61615803:C:G	rs174589	61615803	0.973	1	1	c	g	0.8774	0.8859	0.0844	0.0297	0.004555	++
DGLA	rs174582	rs174582	61607168	1	1	1	a	g	0.8891	0.9021	-0.1239	0.0439	0.0048	--
STE	rs174466:61659296:A:G	rs174466	61659296	0.966	1	1	a	g	0.359	0.3611	0.0451	0.016	0.004815	++
STE	rs1151144:61656585:T:C	rs1151144	61656585	0.998	1	1	t	c	0.3474	0.3528	0.0441	0.0157	0.004923	++
DHA	rs174472:61671956:A:G	rs174472	61671956	0.975	1	1	a	g	0.2874	0.2929	-0.0771	0.0276	0.005203	--
STE	JHU_11.61657592	rs68112215	61657593	1	1	1	t	c	0.6485	0.6546	-0.0437	0.0157	0.005315	--
DPA6	11:61621194-G-A	rs174598	61621194	1	1	1	a	g	0.2131	0.2167	-0.0966	0.0348	0.005454	--
STE	rs2727263:61618012:C:T	rs2727263	61618012	0.975	1	1	t	c	0.086	0.0874	-0.0701	0.0253	0.005704	--
DPA6	rs174593	rs174593	61618831	1	1	1	t	c	0.8414	0.852	0.1088	0.0395	0.005839	++
AA	rs174590:61616977:G:C	rs174590	61616977	0.981	1	1	c	g	0.1133	0.122	-0.0816	0.0296	0.005859	--
DPA6	rs174591	rs174591	61617676	0.988	1	1	a	t	0.1468	0.1536	-0.1094	0.04	0.006238	--
EDA	rs2727263:61618012:C:T	rs2727263	61618012	0.975	1	1	t	c	0.086	0.0874	0.1311	0.0481	0.00643	++
SQRAT63	rs174458:61656995:C:T	rs174458	61656995	1	1	1	t	c	0.6214	0.6259	-0.0998	0.0369	0.006858	--
SQRAT63	rs174465:61658800:C:T	rs174465	61658800	0.98	1	1	t	c	0.6222	0.628	-0.1009	0.0373	0.006903	--
ARA	rs2727263:61618012:C:T	rs2727263	61618012	0.975	1	1	t	c	0.086	0.0874	-0.0881	0.0328	0.007256	--
DPA6	rs2851682	rs2851682	61616012	0.983	1	1	a	g	0.9162	0.9209	0.148	0.0552	0.007347	++
DPA6	rs174596:61620734:T:C	rs174596	61620734	0.937	1	1	t	c	0.8593	0.8649	0.1158	0.0432	0.007399	++
DPA	rs174472:61671956:A:G	rs174472	61671956	0.975	1	1	a	g	0.2874	0.2929	-0.109	0.0407	0.007483	--
DPA6	11:61622227-T-C	rs174600	61622227	0.996	1	1	t	c	0.7814	0.7847	0.0924	0.0348	0.007883	++
STE	rs526126	rs526126	61624885	1	1	1	c	g	0.8841	0.895	0.0627	0.0236	0.007985	++
SQRAT63	11:61657110-C-T	rs174460	61657110	1	1	1	t	c	0.6207	0.6262	-0.0977	0.037	0.008219	--
PLA	rs174449	rs174449	61640379	1	1	1	a	g	0.5785	0.5921	-0.1038	0.0395	0.00855	--
SQRAT63	rs174455	rs174455	61656117	1	1	1	a	g	0.5959	0.6002	-0.0984	0.0375	0.008683	--

DGLA	rs174579	rs174579	61605613	0.998	1	1	t	c	0.0912	0.1069	0.1172	0.0447	0.008739	++
DTA	11:61580635-C-T	rs174556	61580635	1	1	1	t	c	0.1697	0.1711	-0.1063	0.0408	0.009112	--
DPA6	rs149597144:61602460:CA:C	rs199985711	61602460	0.978	1	1	ca	c	0.8802	0.8863	0.1167	0.0448	0.009128	++
BEH	11:61548559-A-G	rs509360	61548559	1	1	1	a	g	0.7226	0.736	-0.0656	0.0253	0.009432	--
ARA	rs2845572:61614595:G:A	rs2845572	61614595	0.98	1	1	a	g	0.0783	0.084	-0.0851	0.0328	0.009438	--
DTA	11:61605499-T-A	rs174578	61605499	1	1	1	a	t	0.1844	0.192	-0.1014	0.0392	0.00976	--
DTA	rs174577	rs174577	61604814	1	1	1	a	c	0.1844	0.192	-0.1014	0.0392	0.00976	--
STE	rs2526678:61623793:G:A	rs2526678	61623793	0.95	1	1	a	g	0.0868	0.0871	-0.0651	0.0253	0.01002	--
LIG	11:61548559-A-G	rs509360	61548559	1	1	1	a	g	0.7226	0.736	-0.0818	0.0318	0.01012	--
SQRAT63	rs1000778	rs1000778	61655305	1	1	1	a	g	0.3391	0.3483	0.0996	0.0388	0.0103	++
DPA6	rs174575	rs174575	61602003	1	1	1	c	g	0.8792	0.8885	0.1141	0.0445	0.01033	++
DTA	11:61604967-C-T	rs2072113	61604967	1	1	1	t	c	0.0757	0.1017	-0.1306	0.051	0.0105	--
ALA	rs3168072	rs3168072	61631510	1	1	1	a	t	0.9312	0.9364	-0.2569	0.1004	0.01052	--
ARA	rs2526678:61623793:G:A	rs2526678	61623793	0.95	1	1	a	g	0.0868	0.0871	-0.0842	0.033	0.01064	--
DTA	11:61603358-A-T	rs2727271	61603358	1	1	1	a	t	0.8983	0.9235	0.1312	0.0514	0.01066	++
DTA	rs2727270:61603237:C:T	rs2727270	61603237	0.998	1	1	t	c	0.0765	0.1012	-0.1312	0.0514	0.01071	--
PAL	11:61629122-G-A	rs174616	61629122	1	1	1	a	g	0.3808	0.387	-0.0314	0.0123	0.01082	--
DTA	rs174544:61567753:C:A	rs174544	61567753	0.997	1	1	a	c	0.1712	0.1719	-0.1036	0.0407	0.01104	--
DPA6	rs2845572:61614595:G:A	rs2845572	61614595	0.98	1	1	a	g	0.0783	0.084	-0.1392	0.0549	0.01124	--
DTA	11:61582708-T-C	rs174561	61582708	1	1	1	t	c	0.827	0.8288	0.103	0.0406	0.01125	++
DTA	rs174549	rs174549	61571382	1	1	1	a	g	0.1712	0.173	-0.103	0.0406	0.01128	--
DTA	rs174555:61579760:T:C	rs174555	61579760	1	1	1	t	c	0.827	0.8288	0.103	0.0406	0.01128	++
DTA	rs174557:61581368:A:G	rs174557	61581368	0.999	1	1	a	g	0.827	0.8285	0.1027	0.0406	0.01134	++
AA	rs174595:61619893:C:T	rs174595	61619893	0.996	1	1	t	c	0.1167	0.1239	-0.0739	0.0292	0.01136	--
STE	rs693672:61658629:T:C	rs693672	61658629	0.979	1	1	t	c	0.2617	0.2653	0.0445	0.0176	0.01146	++
STE	rs174635:61647427:T:G	rs174635	61647427	0.969	1	1	t	g	0.355	0.3658	0.0402	0.0159	0.01159	++
PLE	rs174450:61641542:G:T	rs174450	61641542	0.959	1	1	t	g	0.5806	0.5931	0.0821	0.0326	0.01166	++
STE	rs2845572:61614595:G:A	rs2845572	61614595	0.98	1	1	a	g	0.0783	0.084	-0.065	0.0258	0.01168	--
ARA	rs2851682	rs2851682	61616012	0.983	1	1	a	g	0.9162	0.9209	0.0822	0.0326	0.01171	++

DTA	11:61606683-G-A	rs174581	61606683	1	1	1	a	g	0.1866	0.1948	-0.0983	0.039	0.01174	--
STE	rs174634:61647387:G:C	rs174634	61647387	0.97	1	1	c	g	0.6341	0.6449	-0.0401	0.0159	0.01177	--
AA	rs2727263:61618012:C:T	rs2727263	61618012	0.975	1	1	t	c	0.086	0.0874	-0.0784	0.0312	0.01203	--
DPA6	11:61600327-G-A	rs174573	61600327	1	1	1	a	g	0.1115	0.12	-0.1118	0.0447	0.01236	--
SQRAT63	rs526126	rs526126	61624885	1	1	1	c	g	0.8841	0.895	-0.1441	0.0577	0.01249	--
ALA	11:61629122-G-A	rs174616	61629122	1	1	1	a	g	0.3808	0.387	0.1352	0.0543	0.01279	++
DTA	rs174576	rs174576	61603510	1	1	1	a	c	0.1866	0.1976	-0.0968	0.0389	0.01284	--
PLA	rs422249	rs422249	61639488	0.976	1	1	t	c	0.1873	0.1953	0.1184	0.0476	0.01289	++
PUFA	rs174582	rs174582	61607168	1	1	1	a	g	0.8891	0.9021	-0.1312	0.0529	0.01313	--
DTA	11:61604782-A-T	rs2524299	61604782	1	1	1	a	t	0.8992	0.9235	0.1267	0.0511	0.01321	++
DTA	rs28456:61589481:A:G	rs28456	61589481	1	1	1	a	g	0.827	0.8278	0.1005	0.0406	0.01331	++
DTA	11:61581764-T-C	rs174560	61581764	1	1	1	t	c	0.827	0.8278	0.1005	0.0406	0.01331	++
DTA	rs174548	rs174548	61571348	1	1	1	c	g	0.827	0.8278	0.1005	0.0406	0.01331	++
PAL	11:61629166-A-G	rs174617	61629166	1	1	1	a	g	0.6101	0.6155	0.0305	0.0123	0.01331	++
ALA	rs174582	rs174582	61607168	1	1	1	a	g	0.8891	0.9021	-0.2398	0.097	0.01338	--
STE	rs2851682	rs2851682	61616012	0.983	1	1	a	g	0.9162	0.9209	0.0634	0.0256	0.01344	++
STE	11:61620274-T-C	rs72643559	61620274	1	1	1	t	c	0.9312	0.9413	0.0705	0.0286	0.01381	++
ARA	rs2526680:61611216:C:T	rs2526680	61611216	0.969	1	1	t	c	0.0679	0.0804	-0.0838	0.034	0.01384	--
STE	rs174455	rs174455	61656117	1	1	1	a	g	0.5959	0.6002	-0.0379	0.0154	0.01401	--
DTA	rs2072114	rs2072114	61605215	1	1	1	a	g	0.8992	0.9235	0.1259	0.0513	0.01406	++
DTA	11:61606642-A-G	rs174580	61606642	1	1	1	a	g	0.8005	0.8108	0.0951	0.0387	0.0141	++
ARA	rs174464_61657926	rs174464	61657926	0.987	1	1	a	g	0.3487	0.358	0.0497	0.0203	0.01412	++
DTA	11:61593816-C-T	rs174568	61593816	1	1	1	t	c	0.1826	0.1827	-0.098	0.04	0.0142	--
ALA	rs12577276:61632310:A:G	rs12577276	61632310	0.968	1	1	a	g	0.857	0.8684	-0.1908	0.0781	0.01461	--
LA	rs174582	rs174582	61607168	1	1	1	a	g	0.8891	0.9021	-0.1448	0.0594	0.01479	--
GLA	rs174582	rs174582	61607168	1	1	1	a	g	0.8891	0.9021	-0.2278	0.0936	0.01496	--
PUFA6	rs174582	rs174582	61607168	1	1	1	a	g	0.8891	0.9021	-0.1299	0.0534	0.01498	--
DTA	rs3834458:61594920:CT:C	rs774882452	61594920	0.996	1	1	ct	c	0.8157	0.8165	0.0964	0.0399	0.01575	++
PAL	rs35622765:61629656:A:ACT	rs35622765	61629656	0.999	1	1	a	act	0.6108	0.6165	0.0297	0.0123	0.0161	++

DHA	11:61647288-G-A	rs7942717	61647288	1	1	1	a	g	0.9008	0.9077	0.1061	0.0442	0.01633	++
SQRAT63	rs422249	rs422249	61639488	0.976	1	1	t	c	0.1873	0.1953	0.1153	0.048	0.01633	++
STE	rs2526680:61611216:C:T	rs2526680	61611216	0.969	1	1	t	c	0.0679	0.0804	-0.0637	0.0265	0.01635	--
EIC9	rs11605884:61630133:T:C	rs11605884	61630133	0.968	1	1	t	c	0.904	0.9089	-0.1197	0.0499	0.01646	--
PLA	11:61657110-C-T	rs174460	61657110	1	1	1	t	c	0.6207	0.6262	-0.0945	0.0394	0.01646	--
PLA	rs174458:61656995:C:T	rs174458	61656995	1	1	1	t	c	0.6214	0.6259	-0.0945	0.0394	0.01651	--
LA	rs174533:61549025:G:A	rs174533	61549025	0.986	1	1	a	g	0.1988	0.2053	0.0948	0.0395	0.01654	++
DGLA	rs174575	rs174575	61602003	1	1	1	c	g	0.8792	0.8885	-0.0974	0.0408	0.01701	+-
LIG	rs102275	rs102275	61557803	1	1	1	t	c	0.7974	0.803	-0.0849	0.0356	0.01714	--
DTA	rs5792235:61596322:CA:C	-	61596322	0.998	1	1	ca	c	0.8131	0.8156	0.0944	0.0396	0.01725	++
DTA	rs174550	rs174550	61571478	1	1	1	t	c	0.8154	0.8156	0.0947	0.0398	0.01735	++
DTA	rs174546	rs174546	61569830	1	1	1	t	c	0.1844	0.1846	-0.0947	0.0398	0.01735	--
DTA	11:61569306-C-G	rs174545	61569306	1	1	1	c	g	0.8154	0.8156	0.0947	0.0398	0.01735	++
DTA	rs174547	rs174547	61570783	1	1	1	t	c	0.8154	0.8156	0.0947	0.0398	0.01735	++
PAL	11:61629666-A-G	rs174619	61629666	1	1	1	a	g	0.3844	0.3893	-0.0293	0.0123	0.01751	--
DTA	rs174551:61573684:T:C	rs174551	61573684	1	1	1	t	c	0.8153	0.8156	0.0945	0.0398	0.0176	++
DPA6	rs174572	rs174572	61598288	1	1	1	t	c	0.1115	0.1208	-0.1071	0.0451	0.01762	--
DHA	rs422249	rs422249	61639488	0.976	1	1	t	c	0.1873	0.1953	-0.0766	0.0323	0.01767	--
DTA	rs174553:61575158:A:G	rs174553	61575158	0.999	1	1	a	g	0.8153	0.8156	0.0944	0.0398	0.01782	++
ARA	rs174466:61659296:A:G	rs174466	61659296	0.966	1	1	a	g	0.359	0.3611	0.0481	0.0204	0.01814	++
DPA	rs76095489:61664711:A:T	rs76095489	61664711	0.907	1	1	a	t	0.9297	0.9365	-0.1824	0.0775	0.01867	--
PAL	rs174615:61628964:T:A	rs174615	61628964	0.998	1	1	a	t	0.3788	0.386	-0.0292	0.0124	0.0187	--
PLE	11:61620274-T-C	rs72643559	61620274	1	1	1	t	c	0.9312	0.9413	-0.1374	0.0585	0.01888	--
PAL	rs174614:61628915:T:C	rs174614	61628915	0.998	1	1	t	c	0.6139	0.6213	0.0292	0.0124	0.01891	++
PLA	rs174465:61658800:C:T	rs174465	61658800	0.98	1	1	t	c	0.6222	0.628	-0.0933	0.0398	0.01891	--
PUFA3	rs3168072	rs3168072	61631510	1	1	1	a	t	0.9312	0.9364	-0.1534	0.0654	0.01901	--
EDA	11:61621194-G-A	rs174598	61621194	1	1	1	a	g	0.2131	0.2167	0.0775	0.0332	0.01951	++
DTA	rs99780	rs99780	61596633	1	1	1	t	c	0.1848	0.1885	-0.092	0.0394	0.01959	--
DTA	rs1535	rs1535	61597972	1	1	1	a	g	0.811	0.8156	0.0924	0.0396	0.01971	++

SQRAT63	rs174635:61647427:T:G	rs174635	61647427	0.969	1	1	t	g	0.355	0.3658	0.0901	0.0387	0.01982	++
SQRAT63	rs174634:61647387:G:C	rs174634	61647387	0.97	1	1	c	g	0.6341	0.6449	-0.0899	0.0386	0.02	--
ELA	rs174448	rs174448	61639573	1	1	1	a	g	0.6442	0.6511	-0.1123	0.0483	0.02007	--
DGLA	rs174572	rs174572	61598288	1	1	1	t	c	0.1115	0.1208	0.096	0.0414	0.02033	-+
EDA	rs57668028:61591995:GAA:G	-	61591995	0.982	1	1	g	gaa	0.163	0.1686	0.0848	0.0367	0.02068	++
PAL	rs174613:61628492:G:A	rs174613	61628492	0.997	1	1	a	g	0.3785	0.3872	-0.0287	0.0124	0.02077	--
ARA	rs1000778	rs1000778	61655305	1	1	1	a	g	0.3391	0.3483	0.0484	0.021	0.02109	++
DTA	rs35473591:61586328:C:CT	rs35473591	61586328	1	1	1	ct	c	0.1844	0.1854	-0.0919	0.0399	0.02122	--
PLE	rs76095489:61664711:A:T	rs76095489	61664711	0.907	1	1	a	t	0.9297	0.9365	-0.132	0.0574	0.0214	--
DTA	11:61585144-A-G	rs174562	61585144	1	1	1	a	g	0.8145	0.8156	0.0917	0.0399	0.02149	++
LIG	11:61557826-T-C	rs102274	61557826	1	1	1	t	c	0.7982	0.8035	-0.0824	0.0358	0.02151	--
LIG	rs174535	rs174535	61551356	1	1	1	t	c	0.7936	0.803	-0.0816	0.0355	0.02159	--
PLE	rs57535397:61641889:G:A	rs57535397	61641889	0.911	1	1	a	g	0.0607	0.0716	0.136	0.0592	0.02173	++
LA	rs57668028:61591995:GAA:G	-	61591995	0.982	1	1	g	gaa	0.163	0.1686	0.0987	0.0431	0.02211	++
EDA	11:61622227-T-C	rs174600	61622227	0.996	1	1	t	c	0.7814	0.7847	-0.0757	0.0331	0.02223	--
DPA6	11:61624181-T-C	rs97384	61624181	1	1	1	t	c	0.2561	0.2744	-0.0729	0.0319	0.02234	--
SQRAT63	rs74771917:61627960:C:T	rs74771917	61627960	0.99	1	1	t	c	0.097	0.0999	-0.1477	0.0647	0.02242	--
ALA	rs174613:61628492:G:A	rs174613	61628492	0.997	1	1	a	g	0.3785	0.3872	0.125	0.0548	0.02252	++
ARA	rs174463:61657838:G:A	rs174463	61657838	0.988	1	1	a	g	0.6465	0.6513	-0.0459	0.0201	0.02254	--
ALA	11:61627811-T-C	rs174609	61627811	1	1	1	t	c	0.6119	0.6219	-0.125	0.0548	0.02263	--
DHA	11:61672645-G-C	rs174475	61672645	1	1	1	c	g	0.1967	0.2125	-0.0715	0.0313	0.02263	+-
ALA	rs174614:61628915:T:C	rs174614	61628915	0.998	1	1	t	c	0.6139	0.6213	-0.1247	0.0547	0.02266	--
ALA	rs174615:61628964:T:A	rs174615	61628964	0.998	1	1	a	t	0.3788	0.386	0.1247	0.0547	0.02268	++
DTA	rs174574	rs174574	61600342	1	1	1	a	c	0.1844	0.1874	-0.0901	0.0395	0.02268	--
DTA	rs174567:61593005:A:G	rs174567	61593005	0.998	1	1	a	g	0.8151	0.8152	0.0905	0.0397	0.02275	++
ALA	11:61629166-A-G	rs174617	61629166	1	1	1	a	g	0.6101	0.6155	-0.1241	0.0545	0.02277	--
EDA	rs174533:61549025:G:A	rs174533	61549025	0.986	1	1	a	g	0.1988	0.2053	0.0788	0.0347	0.02314	++
EIC9	11:61647288-G-A	rs7942717	61647288	1	1	1	a	g	0.9008	0.9077	0.1033	0.0455	0.0232	++
DGLA	11:61600327-G-A	rs174573	61600327	1	1	1	a	g	0.1115	0.12	0.094	0.0415	0.0234	-+

STE	rs11353704:61648227:AT:A	-	61648227	0.941	1	1	a	at	0.6375	0.6466	-0.0363	0.016	0.02355	--
LA	11:61548559-A-G	rs509360	61548559	1	1	1	a	g	0.7226	0.736	-0.0837	0.037	0.02369	--
MUFA	11:61629166-A-G	rs174617	61629166	1	1	1	a	g	0.6101	0.6155	0.0272	0.012	0.0237	++
LIG	rs174533:61549025:G:A	rs174533	61549025	0.986	1	1	a	g	0.1988	0.2053	0.0793	0.0351	0.02383	++
PAL	rs174612:61628266:C:G	rs174612	61628266	0.997	1	1	c	g	0.6079	0.6132	0.0278	0.0123	0.02398	++
LA	rs174537	rs174537	61552680	1	1	1	t	g	0.194	0.2002	0.0912	0.0405	0.02428	++
ARA	rs174461:61657401:A:C	rs174461	61657401	0.993	1	1	a	c	0.3485	0.3535	0.0453	0.0201	0.02446	++
DPA6	rs174595:61619893:C:T	rs174595	61619893	0.996	1	1	t	c	0.1167	0.1239	-0.0973	0.0433	0.02458	--
DPA6	rs174589:61615803:C:G	rs174589	61615803	0.973	1	1	c	g	0.8774	0.8859	0.1	0.0445	0.02466	++
AA	rs2526678:61623793:G:A	rs2526678	61623793	0.95	1	1	a	g	0.0868	0.0871	-0.0705	0.0314	0.02467	--
ALA	rs35622765:61629656:A:ACT	rs35622765	61629656	0.999	1	1	a	act	0.6108	0.6165	-0.1229	0.0547	0.02471	--
ALA	11:61629666-A-G	rs174619	61629666	1	1	1	a	g	0.3844	0.3893	0.1227	0.0547	0.0248	++
OLE	11:61629166-A-G	rs174617	61629166	1	1	1	a	g	0.6101	0.6155	0.0274	0.0122	0.02497	++
PAL	11:61627811-T-C	rs174609	61627811	1	1	1	t	c	0.6119	0.6219	0.0278	0.0124	0.025	++
DTA	rs174564:61588305:A:G	rs174564	61588305	1	1	1	a	g	0.8146	0.8152	0.0891	0.0398	0.02509	++
MUFA	rs11407273:61627411:C:CT	rs11407273	61627411	0.988	1	1	ct	c	0.6062	0.6108	0.0269	0.012	0.02509	++
DHA	rs28456:61589481:A:G	rs28456	61589481	1	1	1	a	g	0.827	0.8278	0.0708	0.0316	0.02516	++
DHA	11:61581764-T-C	rs174560	61581764	1	1	1	t	c	0.827	0.8278	0.0708	0.0316	0.02516	++
DHA	rs174548	rs174548	61571348	1	1	1	c	g	0.827	0.8278	0.0708	0.0316	0.02516	++
EPA	rs74771917:61627960:C:T	rs74771917	61627960	0.99	1	1	t	c	0.097	0.0999	0.1862	0.0833	0.02541	++
OLE	rs11407273:61627411:C:CT	rs11407273	61627411	0.988	1	1	ct	c	0.6062	0.6108	0.0273	0.0122	0.02543	++
PUFA6	rs174533:61549025:G:A	rs174533	61549025	0.986	1	1	a	g	0.1988	0.2053	0.0797	0.0357	0.02566	++
LA	11:61621194-G-A	rs174598	61621194	1	1	1	a	g	0.2131	0.2167	0.0843	0.0378	0.0257	++
ARA	JHU_11.61657592	rs68112215	61657593	1	1	1	t	c	0.6485	0.6546	-0.045	0.0202	0.02572	--
PLA	rs174455	rs174455	61656117	1	1	1	a	g	0.5959	0.6002	-0.0869	0.039	0.02591	--
ARA	rs36047269:61656254:T:TC	rs36047269	61656254	0.997	1	1	t	tc	0.3466	0.3528	0.0449	0.0202	0.02613	++
ARA	rs174456:61656182:C:A	rs174456	61656182	0.997	1	1	a	c	0.6472	0.6535	-0.0449	0.0202	0.02613	--
ARA	rs5792240:61656541:A:AT	rs5792240	61656541	0.998	1	1	a	at	0.3467	0.3528	0.0448	0.0202	0.02623	++
ARA	rs1295988:61656658:A:C	rs1295988	61656658	0.998	1	1	a	c	0.3467	0.3528	0.0448	0.0202	0.02625	++

CAP	rs1151139:61671156:C:T	rs1151139	61671156	0.986	1	1	t	c	0.2938	0.3011	-0.1211	0.0545	0.02634	--
ARA	rs174457:61656975:T:C	rs174457	61656975	0.998	1	1	t	c	0.3469	0.3528	0.0448	0.0202	0.02636	++
LA	11:61622227-T-C	rs174600	61622227	0.996	1	1	t	c	0.7814	0.7847	-0.0835	0.0376	0.0265	--
MUFA	11:61627833-A-G	rs174610	61627833	1	1	1	a	g	0.6067	0.6137	0.0265	0.012	0.02691	++
PAL	11:61627833-A-G	rs174610	61627833	1	1	1	a	g	0.6067	0.6137	0.0272	0.0123	0.02712	++
MUFA	rs174612:61628266:C:G	rs174612	61628266	0.997	1	1	c	g	0.6079	0.6132	0.0264	0.012	0.02747	++
ARA	rs174459:61657050:C:T	rs174459	61657050	0.998	1	1	t	c	0.6472	0.6527	-0.0444	0.0202	0.02758	--
MUFA	11:61629122-G-A	rs174616	61629122	1	1	1	a	g	0.3808	0.387	-0.0265	0.012	0.02768	--
EDA	rs174565:61591636:C:G	rs174565	61591636	0.997	1	1	c	g	0.8668	0.8706	-0.0837	0.038	0.02775	--
OLE	11:61629122-G-A	rs174616	61629122	1	1	1	a	g	0.3808	0.387	-0.0269	0.0122	0.02777	--
MUFA	11:61627811-T-C	rs174609	61627811	1	1	1	t	c	0.6119	0.6219	0.0264	0.012	0.02828	++
PUFA3	rs174582	rs174582	61607168	1	1	1	a	g	0.8891	0.9021	-0.1293	0.059	0.02833	--
ARA	rs2727265:61609004:C:A	rs2727265	61609004	0.989	1	1	a	c	0.0635	0.0764	-0.0748	0.0341	0.02843	--
DPA6	rs174597:61621040:G:C	rs174597	61621040	0.986	1	1	c	g	0.1246	0.128	-0.0932	0.0427	0.02897	--
OLE	11:61627833-A-G	rs174610	61627833	1	1	1	a	g	0.6067	0.6137	0.0266	0.0122	0.02903	++
GLA	rs2235093:61665122:A:G	rs2235093	61665122	0.975	1	1	a	g	0.2414	0.2581	0.1238	0.0568	0.02933	++
SQRAT63	rs174450:61641542:G:T	rs174450	61641542	0.959	1	1	t	g	0.5806	0.5931	-0.0834	0.0383	0.02943	--
MUFA	rs174613:61628492:G:A	rs174613	61628492	0.997	1	1	a	g	0.3785	0.3872	-0.0262	0.012	0.02949	--
SQRAT63	rs174456:61656182:C:A	rs174456	61656182	0.997	1	1	a	c	0.6472	0.6535	-0.0826	0.038	0.02954	--
SQRAT63	rs36047269:61656254:T:TC	rs36047269	61656254	0.997	1	1	t	tc	0.3466	0.3528	0.0826	0.038	0.0297	++
ARA	rs1151144:61656585:T:C	rs1151144	61656585	0.998	1	1	t	c	0.3474	0.3528	0.0439	0.0202	0.02971	++
OLE	rs174612:61628266:C:G	rs174612	61628266	0.997	1	1	c	g	0.6079	0.6132	0.0265	0.0122	0.02974	++
PLE	rs72643557:61579427:C:T	rs72643557	61579427	0.936	1	1	t	c	0.0607	0.0734	0.1264	0.0582	0.02989	++
MUFA	rs174606:61626973:G:T	rs174606	61626973	0.977	1	1	t	g	0.3876	0.3941	-0.0264	0.0121	0.02991	--
OLE	11:61627811-T-C	rs174609	61627811	1	1	1	t	c	0.6119	0.6219	0.0266	0.0123	0.02994	++
ALA	rs174589:61615803:C:G	rs174589	61615803	0.973	1	1	c	g	0.8774	0.8859	-0.1885	0.0869	0.03	--
PUFA3	rs174597:61621040:G:C	rs174597	61621040	0.986	1	1	c	g	0.1246	0.128	0.1113	0.0513	0.03012	++
MUFA	rs174614:61628915:T:C	rs174614	61628915	0.998	1	1	t	c	0.6139	0.6213	0.0261	0.012	0.03022	++
EDA	rs174570	rs174570	61597212	1	1	1	t	c	0.1279	0.1324	0.083	0.0383	0.03025	++

MUFA	rs174615:61628964:T:A	rs174615	61628964	0.998	1	1	a	t	0.3788	0.386	-0.0261	0.012	0.0303	--
PUFA	rs174533:61549025:G:A	rs174533	61549025	0.986	1	1	a	g	0.1988	0.2053	0.0768	0.0355	0.03035	++
SQRAT63	rs5792240:61656541:A:AT	rs5792240	61656541	0.998	1	1	a	at	0.3467	0.3528	0.0822	0.038	0.03039	++
PUFA3	rs12577276:61632310:A:G	rs12577276	61632310	0.968	1	1	a	g	0.857	0.8684	-0.1101	0.0508	0.03041	--
CAP	rs11407273:61627411:C:CT	rs11407273	61627411	0.988	1	1	ct	c	0.6062	0.6108	-0.1082	0.05	0.03054	--
PUFA3	rs528285	rs528285	61660704	0.955	1	1	a	g	0.3479	0.3508	0.0724	0.0335	0.03061	++
SQRAT63	rs1295988:61656658:A:C	rs1295988	61656658	0.998	1	1	a	c	0.3467	0.3528	0.082	0.038	0.03067	++
CAP	11:61629166-A-G	rs174617	61629166	1	1	1	a	g	0.6101	0.6155	-0.1083	0.0502	0.03093	--
DPA6	rs174590:61616977:G:C	rs174590	61616977	0.981	1	1	c	g	0.1133	0.122	-0.0945	0.0438	0.031	--
PLA	rs174635:61647427:T:G	rs174635	61647427	0.969	1	1	t	g	0.355	0.3658	0.0889	0.0413	0.03112	++
PLA	rs174634:61647387:G:C	rs174634	61647387	0.97	1	1	c	g	0.6341	0.6449	-0.0888	0.0412	0.03132	--
MUFA	11:61629666-A-G	rs174619	61629666	1	1	1	a	g	0.3844	0.3893	-0.026	0.0121	0.03139	--
SQRAT63	rs174457:61656975:T:C	rs174457	61656975	0.998	1	1	t	c	0.3469	0.3528	0.0816	0.0379	0.03143	++
OLE	rs174613:61628492:G:A	rs174613	61628492	0.997	1	1	a	g	0.3785	0.3872	-0.0264	0.0123	0.03148	--
SQRAT63	rs174459:61657050:C:T	rs174459	61657050	0.998	1	1	t	c	0.6472	0.6527	-0.0816	0.038	0.03151	--
TFA	11:61548559-A-G	rs509360	61548559	1	1	1	a	g	0.7226	0.736	-0.0823	0.0383	0.03168	--
LIG	rs174536	rs174536	61551927	1	1	1	a	c	0.7916	0.7994	-0.0756	0.0352	0.03169	--
EPA	rs57535397:61641889:G:A	rs57535397	61641889	0.911	1	1	a	g	0.0607	0.0716	0.2223	0.1036	0.03197	++
OLE	rs174606:61626973:G:T	rs174606	61626973	0.977	1	1	t	g	0.3876	0.3941	-0.0265	0.0124	0.03198	--
GLA	rs174472:61671956:A:G	rs174472	61671956	0.975	1	1	a	g	0.2874	0.2929	0.119	0.0555	0.03218	++
PAL	rs174606:61626973:G:T	rs174606	61626973	0.977	1	1	t	g	0.3876	0.3941	-0.0266	0.0124	0.03221	--
OLE	rs174614:61628915:T:C	rs174614	61628915	0.998	1	1	t	c	0.6139	0.6213	0.0262	0.0123	0.03239	++
DHA	11:61582708-T-C	rs174561	61582708	1	1	1	t	c	0.827	0.8288	0.0675	0.0316	0.0324	++
DHA	rs174549	rs174549	61571382	1	1	1	a	g	0.1712	0.173	-0.0675	0.0316	0.03243	--
DHA	rs174555:61579760:T:C	rs174555	61579760	1	1	1	t	c	0.827	0.8288	0.0675	0.0316	0.03243	++
DHA	rs174557:61581368:A:G	rs174557	61581368	0.999	1	1	a	g	0.827	0.8285	0.0674	0.0315	0.03244	++
OLE	rs174615:61628964:T:A	rs174615	61628964	0.998	1	1	a	t	0.3788	0.386	-0.0262	0.0123	0.0325	--
OLE	11:61629666-A-G	rs174619	61629666	1	1	1	a	g	0.3844	0.3893	-0.0263	0.0123	0.03255	--
LAU	11:61629166-A-G	rs174617	61629166	1	1	1	a	g	0.6101	0.6155	-0.0814	0.0381	0.0326	--

STE	rs72643557:61579427:C:T	rs72643557	61579427	0.936	1	1	t	c	0.0607	0.0734	-0.0616	0.0289	0.03279	--
EPA	rs72643557:61579427:C:T	rs72643557	61579427	0.936	1	1	t	c	0.0607	0.0734	0.2057	0.0965	0.03305	++
PUFA3	rs174589:61615803:C:G	rs174589	61615803	0.973	1	1	c	g	0.8774	0.8859	-0.1128	0.053	0.03324	--
EDA	rs174535	rs174535	61551356	1	1	1	t	c	0.7936	0.803	-0.0741	0.0349	0.0334	--
CAP	rs174612:61628266:C:G	rs174612	61628266	0.997	1	1	c	g	0.6079	0.6132	-0.1064	0.0501	0.03348	--
MUFA	rs35622765:61629656:A:ACT	rs35622765	61629656	0.999	1	1	a	act	0.6108	0.6165	0.0257	0.0121	0.03383	++
ARA	rs174589:61615803:C:G	rs174589	61615803	0.973	1	1	c	g	0.8774	0.8859	-0.0664	0.0313	0.03397	--
DGLA	rs2727270:61603237:C:T	rs2727270	61603237	0.998	1	1	t	c	0.0765	0.1012	-0.0822	0.0388	0.03404	--
SQRAT63	rs1151144:61656585:T:C	rs1151144	61656585	0.998	1	1	t	c	0.3474	0.3528	0.0805	0.038	0.03405	++
PUFA6	rs57668028:61591995:GAA:G	-	61591995	0.982	1	1	g	gaa	0.163	0.1686	0.0823	0.0388	0.03406	++
ELA	rs174458:61656995:C:T	rs174458	61656995	1	1	1	t	c	0.6214	0.6259	-0.1015	0.0479	0.03415	--
PLA	rs76656467	rs76656467	61649030	1	1	1	c	g	0.0599	0.0693	0.1632	0.0771	0.03423	-+
EDA	rs108499:61547237:C:T	rs108499	61547237	0.985	1	1	t	c	0.1734	0.1744	0.0772	0.0365	0.03433	++
PUFA3	rs57535397:61641889:G:A	rs57535397	61641889	0.911	1	1	a	g	0.0607	0.0716	0.1414	0.0668	0.03435	++
SQRAT63	rs174466:61659296:A:G	rs174466	61659296	0.966	1	1	a	g	0.359	0.3611	0.0816	0.0386	0.03445	++
DGLA	11:61603358-A-T	rs2727271	61603358	1	1	1	a	t	0.8983	0.9235	0.082	0.0388	0.03459	++
LAU	rs2727265:61609004:C:A	rs2727265	61609004	0.989	1	1	a	c	0.0635	0.0764	0.1287	0.0609	0.03468	++
STE	rs174449	rs174449	61640379	1	1	1	a	g	0.5785	0.5921	-0.0319	0.0151	0.03473	--
CAP	rs174615:61628964:T:A	rs174615	61628964	0.998	1	1	a	t	0.3788	0.386	0.1063	0.0504	0.03489	++
DPA	11:61621194-G-A	rs174598	61621194	1	1	1	a	g	0.2131	0.2167	0.0879	0.0417	0.03494	++
ELA	11:61657110-C-T	rs174460	61657110	1	1	1	t	c	0.6207	0.6262	-0.101	0.0479	0.03494	--
SQRAT63	rs528285	rs528285	61660704	0.955	1	1	a	g	0.3479	0.3508	-0.0768	0.0364	0.03498	--
CAP	rs174614:61628915:T:C	rs174614	61628915	0.998	1	1	t	c	0.6139	0.6213	-0.1062	0.0504	0.03512	--
OLE	11:61579463-A-G	rs174554	61579463	1	1	1	a	g	0.8156	0.8156	0.0388	0.0184	0.03514	?+
EPA	rs174472:61671956:A:G	rs174472	61671956	0.975	1	1	a	g	0.2874	0.2929	-0.1066	0.0506	0.03528	--
SQRAT63	rs174463:61657838:G:A	rs174463	61657838	0.988	1	1	a	g	0.6465	0.6513	-0.08	0.0381	0.03551	--
SQRAT63	rs11353704:61648227:AT:A	-	61648227	0.941	1	1	a	at	0.6375	0.6466	-0.0828	0.0394	0.03554	--
ELA	rs174465:61658800:C:T	rs174465	61658800	0.98	1	1	t	c	0.6222	0.628	-0.1017	0.0484	0.03558	--
EIC9	rs174594:61619829:C:A	rs174594	61619829	0.997	1	1	a	c	0.7473	0.7596	0.0622	0.0296	0.0356	++

LA	11:61549458-A-G	rs174534	61549458	1	1	1	a	g	0.813	0.8152	-0.0859	0.0409	0.03569	--
DGLA	rs149597144:61602460:CA:C	rs199985711	61602460	0.978	1	1	ca	c	0.8802	0.8863	-0.0853	0.0406	0.0357	+-
OLE	rs35622765:61629656:A:ACT	rs35622765	61629656	0.999	1	1	a	act	0.6108	0.6165	0.0258	0.0123	0.03584	++
ARA	rs174465:61658800:C:T	rs174465	61658800	0.98	1	1	t	c	0.6222	0.628	-0.0419	0.02	0.03587	--
LA	11:61557826-T-C	rs102274	61557826	1	1	1	t	c	0.7982	0.8035	-0.0848	0.0404	0.0359	--
PAL	rs174620:61629747:A:G	rs174620	61629747	0.996	1	1	a	g	0.7131	0.7207	0.0279	0.0133	0.0359	++
LAU	rs11407273:61627411:C:CT	rs11407273	61627411	0.988	1	1	ct	c	0.6062	0.6108	-0.0793	0.0378	0.03603	--
CAP	11:61627833-A-G	rs174610	61627833	1	1	1	a	g	0.6067	0.6137	-0.1046	0.05	0.03635	--
ELA	rs174449	rs174449	61640379	1	1	1	a	g	0.5785	0.5921	-0.0975	0.0466	0.03654	--
ALA	rs174597:61621040:G:C	rs174597	61621040	0.986	1	1	c	g	0.1246	0.128	0.1753	0.084	0.03688	+-
CAP	rs174613:61628492:G:A	rs174613	61628492	0.997	1	1	a	g	0.3785	0.3872	0.1049	0.0503	0.03722	++
EIC9	11:61618608-A-G	rs174592	61618608	1	1	1	a	g	0.7478	0.7589	0.0617	0.0296	0.03722	++
DPA	11:61622227-T-C	rs174600	61622227	0.996	1	1	t	c	0.7814	0.7847	-0.0865	0.0415	0.03733	--
CAP	rs174606:61626973:G:T	rs174606	61626973	0.977	1	1	t	g	0.3876	0.3941	0.1049	0.0504	0.03745	++
SQRAT63	rs174461:61657401:A:C	rs174461	61657401	0.993	1	1	a	c	0.3485	0.3535	0.0791	0.0381	0.03752	++
ARA	rs2524296:61601378:C:T	rs2524296	61601378	0.997	1	1	t	c	0.0634	0.0773	-0.0704	0.0339	0.03768	--
DHA	rs2727270:61603237:C:T	rs2727270	61603237	0.998	1	1	t	c	0.0765	0.1012	-0.0833	0.0401	0.0378	--
PUFA	11:61621194-G-A	rs174598	61621194	1	1	1	a	g	0.2131	0.2167	0.0701	0.0337	0.03784	++
SQRAT63	rs174454:61650747:G:A	rs174454	61650747	0.963	1	1	a	g	0.6633	0.6834	-0.0832	0.0401	0.03784	--
PUFA6	11:61548559-A-G	rs509360	61548559	1	1	1	a	g	0.7226	0.736	-0.069	0.0333	0.03789	--
ARA	rs693672:61658629:T:C	rs693672	61658629	0.979	1	1	t	c	0.2617	0.2653	0.0459	0.0221	0.03798	++
DHA	rs174544:61567753:C:A	rs174544	61567753	0.997	1	1	a	c	0.1712	0.1719	-0.0659	0.0318	0.03804	--
BEH	11:61621194-G-A	rs174598	61621194	1	1	1	a	g	0.2131	0.2167	0.0561	0.027	0.03806	++
DHA	11:61603358-A-T	rs2727271	61603358	1	1	1	a	t	0.8983	0.9235	0.0832	0.0401	0.03808	++
BEH	11:61620274-T-C	rs72643559	61620274	1	1	1	t	c	0.9312	0.9413	0.1102	0.0531	0.03811	++
PUFA6	11:61621194-G-A	rs174598	61621194	1	1	1	a	g	0.2131	0.2167	0.0707	0.0341	0.03821	++
DTA	rs4564341:61573540:C:T	rs4564341	61573540	0.935	1	1	t	c	0.0606	0.0673	-0.13	0.0627	0.03825	--
PLA	rs11353704:61648227:AT:A	-	61648227	0.941	1	1	a	at	0.6375	0.6466	-0.087	0.042	0.03837	--
CAP	11:61629666-A-G	rs174619	61629666	1	1	1	a	g	0.3844	0.3893	0.1042	0.0504	0.03845	++

CAP	rs35622765:61629656:A:ACT	rs35622765	61629656	0.999	1	1	a	act	0.6108	0.6165	-0.1042	0.0504	0.03868	--
ARA	rs2845574:61601872:C:T	rs2845574	61601872	0.997	1	1	t	c	0.0634	0.0774	-0.0699	0.0338	0.03887	--
DGLA	rs174611	rs174611	61627881	0.972	1	1	t	c	0.8956	0.9069	-0.0819	0.0397	0.03892	--
DHA	11:61604782-A-T	rs2524299	61604782	1	1	1	a	t	0.8992	0.9235	0.0831	0.0403	0.03894	++
ARA	rs2845573:61601908:A:G	rs2845573	61601908	0.997	1	1	a	g	0.9226	0.9366	0.0698	0.0338	0.03897	++
PUFA6	rs174537	rs174537	61552680	1	1	1	t	g	0.194	0.2002	0.0754	0.0365	0.03907	++
ALA	rs174590:61616977:G:C	rs174590	61616977	0.981	1	1	c	g	0.1133	0.122	0.1785	0.0865	0.03914	++
PAL	rs174618	rs174618	61629322	1	1	1	t	c	0.714	0.72	0.0272	0.0132	0.0392	++
EDA	rs102275	rs102275	61557803	1	1	1	t	c	0.7974	0.803	-0.072	0.0349	0.03928	--
PUFA	11:61622227-T-C	rs174600	61622227	0.996	1	1	t	c	0.7814	0.7847	-0.0691	0.0336	0.03966	--
DHA	11:61580635-C-T	rs174556	61580635	1	1	1	t	c	0.1697	0.1711	-0.0656	0.0319	0.03967	--
LAU	rs174612:61628266:C:G	rs174612	61628266	0.997	1	1	c	g	0.6079	0.6132	-0.0779	0.0379	0.03973	--
DGLA	rs174605:61626921:G:T	rs174605	61626921	0.96	1	1	t	g	0.091	0.1025	0.083	0.0405	0.04011	++
MYR	rs2845573:61601908:A:G	rs2845573	61601908	0.997	1	1	a	g	0.9226	0.9366	-0.1261	0.0615	0.04021	--
PUFA6	11:61622227-T-C	rs174600	61622227	0.996	1	1	t	c	0.7814	0.7847	-0.0697	0.034	0.04023	--
BEH	11:61622227-T-C	rs174600	61622227	0.996	1	1	t	c	0.7814	0.7847	-0.0553	0.027	0.04025	--
LA	rs108499:61547237:C:T	rs108499	61547237	0.985	1	1	t	c	0.1734	0.1744	0.088	0.0429	0.04027	++
MYR	rs2845574:61601872:C:T	rs2845574	61601872	0.997	1	1	t	c	0.0634	0.0774	0.1261	0.0615	0.0403	++
LAU	11:61629666-A-G	rs174619	61629666	1	1	1	a	g	0.3844	0.3893	0.0784	0.0382	0.04043	++
ALA	rs174612:61628266:C:G	rs174612	61628266	0.997	1	1	c	g	0.6079	0.6132	-0.1119	0.0546	0.04047	--
SQRAT63	JHU_11.61657592	rs68112215	61657593	1	1	1	t	c	0.6485	0.6546	-0.0778	0.038	0.04071	--
LAU	rs2845573:61601908:A:G	rs2845573	61601908	0.997	1	1	a	g	0.9226	0.9366	-0.1233	0.0603	0.04075	--
ALA	11:61627833-A-G	rs174610	61627833	1	1	1	a	g	0.6067	0.6137	-0.1115	0.0545	0.04084	--
ARA	rs138766446:61602458:CCCA:C	rs765564188	61602458	0.992	1	1	ccca	c	0.9218	0.9365	0.069	0.0338	0.04085	++
LAU	rs2845574:61601872:C:T	rs2845574	61601872	0.997	1	1	t	c	0.0634	0.0774	0.1232	0.0603	0.04089	++
PUFA3	rs174590:61616977:G:C	rs174590	61616977	0.981	1	1	c	g	0.1133	0.122	0.1076	0.0527	0.04105	++
PUFA	rs57668028:61591995:GAA:G	-	61591995	0.982	1	1	g	gaa	0.163	0.1686	0.0784	0.0384	0.04108	++
SQRAT63	rs174464_61657926	rs174464	61657926	0.987	1	1	a	g	0.3487	0.358	0.0782	0.0383	0.04113	++

DGLA	rs174608:61627484:A:G	rs174608	61627484	0.968	1	1	a	g	0.8957	0.9068	-0.0813	0.0399	0.04127	--
CAP	11:61629122-G-A	rs174616	61629122	1	1	1	a	g	0.3808	0.387	0.1021	0.0501	0.04129	++
MYR	rs2524296:61601378:C:T	rs2524296	61601378	0.997	1	1	t	c	0.0634	0.0773	0.1255	0.0615	0.04142	++
LA	rs174538	rs174538	61560081	0.998	1	1	a	g	0.1683	0.1741	0.087	0.0427	0.04145	++
ALA	11:61622227-T-C	rs174600	61622227	0.996	1	1	t	c	0.7814	0.7847	-0.1269	0.0623	0.04156	--
OLE	rs528285	rs528285	61660704	0.955	1	1	a	g	0.3479	0.3508	-0.0244	0.012	0.04161	--
CAP	11:61627811-T-C	rs174609	61627811	1	1	1	t	c	0.6119	0.6219	-0.1024	0.0503	0.04168	--
EDA	rs174538	rs174538	61560081	0.998	1	1	a	g	0.1683	0.1741	0.0736	0.0362	0.04182	++
EDA	11:61557826-T-C	rs102274	61557826	1	1	1	t	c	0.7982	0.8035	-0.0713	0.0351	0.04187	--
STE	rs6591668:61661113:T:C	rs6591668	61661113	0.976	1	1	t	c	0.0547	0.0589	0.0657	0.0324	0.0422	++
ARA	11:61604967-C-T	rs2072113	61604967	1	1	1	t	c	0.0757	0.1017	-0.0632	0.0312	0.0426	--
LAU	rs2524296:61601378:C:T	rs2524296	61601378	0.997	1	1	t	c	0.0634	0.0773	0.1222	0.0603	0.04266	++
MYR	rs2727265:61609004:C:A	rs2727265	61609004	0.989	1	1	a	c	0.0635	0.0764	0.1265	0.0625	0.04284	++
LAU	rs138766446:61602458:CCCA:C	rs765564188	61602458	0.992	1	1	cc ca	c	0.9218	0.9365	-0.1217	0.0601	0.04296	--
AA	rs174597:61621040:G:C	rs174597	61621040	0.986	1	1	c	g	0.1246	0.128	-0.0578	0.0285	0.04298	--
TFA	rs13966	rs13966	61664992	1	1	1	t	c	0.2541	0.2783	0.075	0.0371	0.04308	++
PUFA	11:61548559-A-G	rs509360	61548559	1	1	1	a	g	0.7226	0.736	-0.0669	0.0331	0.04315	--
CAP	rs174470:61669608:G:A	rs174470	61669608	0.993	1	1	a	g	0.2961	0.3038	-0.1095	0.0542	0.04319	--
LAU	rs174606:61626973:G:T	rs174606	61626973	0.977	1	1	t	g	0.3876	0.3941	0.0776	0.0384	0.04322	++
ARA	rs174590:61616977:G:C	rs174590	61616977	0.981	1	1	c	g	0.1133	0.122	0.0637	0.0315	0.04343	++
NER	rs11605884:61630133:T:C	rs11605884	61630133	0.968	1	1	t	c	0.904	0.9089	-0.1676	0.083	0.04346	--
ALA	11:61621194-G-A	rs174598	61621194	1	1	1	a	g	0.2131	0.2167	0.1259	0.0625	0.04378	++
DTA	rs174559:61581656:G:A	rs174559	61581656	0.999	1	1	a	g	0.1566	0.1611	-0.084	0.0417	0.0438	--
LAU	rs35622765:61629656:A:ACT	rs35622765	61629656	0.999	1	1	a	act	0.6108	0.6165	-0.0771	0.0383	0.0438	--
AA	rs174602	rs174602	61624414	1	1	1	t	c	0.7776	0.7822	0.043	0.0213	0.04384	++
MYR	rs138766446:61602458:CCCA:C	rs765564188	61602458	0.992	1	1	cc ca	c	0.9218	0.9365	-0.1237	0.0614	0.04397	--
EDA	rs174537	rs174537	61552680	1	1	1	t	g	0.194	0.2002	0.0699	0.0347	0.04416	++
LLA	11:61548559-A-G	rs509360	61548559	1	1	1	a	g	0.7226	0.736	-0.0874	0.0434	0.04428	--

DGLA	rs526126	rs526126	61624885	1	1	1	c	g	0.8841	0.895	-0.0774	0.0385	0.04438	--
LAU	11:61627833-A-G	rs174610	61627833	1	1	1	a	g	0.6067	0.6137	-0.076	0.0378	0.04446	--
EDA	rs174559:61581656:G:A	rs174559	61581656	0.999	1	1	a	g	0.1566	0.1611	0.075	0.0373	0.04452	++
STE	rs7113281:61662780:G:A	rs7113281	61662780	0.984	1	1	a	g	0.94	0.945	-0.0642	0.032	0.04455	--
LA	rs102275	rs102275	61557803	1	1	1	t	c	0.7974	0.803	-0.0808	0.0402	0.04465	--
DGLA	11:61604967-C-T	rs2072113	61604967	1	1	1	t	c	0.0757	0.1017	-0.0778	0.0387	0.04474	--
DPA	11:61672645-G-C	rs174475	61672645	1	1	1	c	g	0.1967	0.2125	-0.0912	0.0455	0.04489	+-
DPA6	11:61579463-A-G	rs174554	61579463	1	1	1	a	g	0.8156	0.8156	0.1095	0.0546	0.04503	?+
PAL	rs11407273:61627411:C:CT	rs11407273	61627411	0.988	1	1	ct	c	0.6062	0.6108	0.0249	0.0124	0.04515	++
PLE	rs174465:61658800:C:T	rs174465	61658800	0.98	1	1	t	c	0.6222	0.628	0.0639	0.0319	0.04517	++
LIG	rs174537	rs174537	61552680	1	1	1	t	g	0.194	0.2002	0.0707	0.0353	0.04534	++
BEH	rs174589:61615803:C:G	rs174589	61615803	0.973	1	1	c	g	0.8774	0.8859	-0.0685	0.0342	0.04547	--
PLE	rs174626	rs174626	61637057	0.982	1	1	a	g	0.5323	0.5363	0.0597	0.0299	0.04583	++
PUFA	rs174537	rs174537	61552680	1	1	1	t	g	0.194	0.2002	0.0722	0.0362	0.04588	++
EPA	rs3168072	rs3168072	61631510	1	1	1	a	t	0.9312	0.9364	-0.1936	0.0971	0.04614	--
DTA	rs7394579:61581450:A:G	rs7394579	61581450	0.984	1	1	a	g	0.8415	0.8467	0.0842	0.0422	0.04616	++
ARA	rs174458:61656995:C:T	rs174458	61656995	1	1	1	t	c	0.6214	0.6259	-0.0395	0.0198	0.04621	--
STE	rs2727265:61609004:C:A	rs2727265	61609004	0.989	1	1	a	c	0.0635	0.0764	-0.0529	0.0265	0.04624	--
PLA	rs116672159:61648113:C:T	rs116672159	61648113	0.968	1	1	t	c	0.0635	0.0721	0.1523	0.0764	0.04626	-+
DTA	rs174583	rs174583	61609750	1	1	1	t	c	0.2006	0.2089	-0.0751	0.0377	0.04654	--
BEH	rs174618	rs174618	61629322	1	1	1	t	c	0.714	0.72	-0.0498	0.025	0.04658	--
DHA	rs174574	rs174574	61600342	1	1	1	a	c	0.1844	0.1874	-0.0613	0.0308	0.04663	--
STE	rs57535397:61641889:G:A	rs57535397	61641889	0.911	1	1	a	g	0.0607	0.0716	-0.0582	0.0293	0.04667	--
DTA	rs174570	rs174570	61597212	1	1	1	t	c	0.1279	0.1324	-0.0913	0.0459	0.0467	-+
DPA6	rs93923:61613745:C:T	rs93923	61613745	0.961	1	1	t	c	0.0971	0.1119	-0.0935	0.047	0.04681	--
ARA	rs2072114	rs2072114	61605215	1	1	1	a	g	0.8992	0.9235	0.0619	0.0311	0.04696	++
EIC9	rs174601	rs174601	61623140	1	1	1	t	c	0.2455	0.265	-0.0581	0.0293	0.04702	--
LA	rs174535	rs174535	61551356	1	1	1	t	c	0.7936	0.803	-0.0796	0.0401	0.04718	--
SQRAT63	rs3168072	rs3168072	61631510	1	1	1	a	t	0.9312	0.9364	0.1546	0.0779	0.0472	++

LA	rs174536	rs174536	61551927	1	1	1	a	c	0.7916	0.7994	-0.0788	0.0397	0.04733	--
EDA	11:61548559-A-G	rs509360	61548559	1	1	1	a	g	0.7226	0.736	-0.0624	0.0315	0.04734	--
EIC9	11:61621556-G-C	rs174599	61621556	0.998	1	1	c	g	0.2431	0.2595	-0.0583	0.0295	0.04783	--
DGLA	rs2072114	rs2072114	61605215	1	1	1	a	g	0.8992	0.9235	0.0766	0.0387	0.04786	++
PUFA3	11:61622227-T-C	rs174600	61622227	0.996	1	1	t	c	0.7814	0.7847	-0.0764	0.0386	0.04807	--
ARA	rs4564341:61573540:C:T	rs4564341	61573540	0.935	1	1	t	c	0.0606	0.0673	-0.0765	0.0387	0.04809	--
PUFA3	11:61621194-G-A	rs174598	61621194	1	1	1	a	g	0.2131	0.2167	0.0764	0.0388	0.04892	++
DHA	rs174465:61658800:C:T	rs174465	61658800	0.98	1	1	t	c	0.6222	0.628	0.0517	0.0263	0.04924	++
PLA	rs6591668:61661113:T:C	rs6591668	61661113	0.976	1	1	t	c	0.0547	0.0589	0.169	0.086	0.04934	++
EDA	11:61549458-A-G	rs174534	61549458	1	1	1	a	g	0.813	0.8152	-0.0696	0.0354	0.04957	--
PLA	rs7113281:61662780:G:A	rs7113281	61662780	0.984	1	1	a	g	0.94	0.945	-0.1672	0.0851	0.04957	--
ALA	rs174579	rs174579	61605613	0.998	1	1	t	c	0.0912	0.1069	0.1901	0.0969	0.04981	++
PLA	rs174463:61657838:G:A	rs174463	61657838	0.988	1	1	a	g	0.6465	0.6513	-0.0789	0.0403	0.04997	--
PUFA	rs174579	rs174579	61605613	0.998	1	1	t	c	0.0912	0.1069	0.1036	0.0529	0.05005	++
GLA	rs13966	rs13966	61664992	1	1	1	t	c	0.2541	0.2783	0.1095	0.0559	0.05024	++
PAL	rs2235093:61665122:A:G	rs2235093	61665122	0.975	1	1	a	g	0.2414	0.2581	-0.0264	0.0135	0.05077	--
BEH	rs174623:61635294:G:A	rs174623	61635294	0.964	1	1	a	g	0.5268	0.5298	-0.0443	0.0227	0.05078	--
BEH	rs174620:61629747:A:G	rs174620	61629747	0.996	1	1	a	g	0.7131	0.7207	-0.0491	0.0251	0.05084	--
PLA	rs174461:61657401:A:C	rs174461	61657401	0.993	1	1	a	c	0.3485	0.3535	0.0784	0.0402	0.05112	++
EIC9	11:61622227-T-C	rs174600	61622227	0.996	1	1	t	c	0.7814	0.7847	0.0615	0.0315	0.05113	++
STE	rs2072114	rs2072114	61605215	1	1	1	a	g	0.8992	0.9235	0.0498	0.0255	0.05117	++
PLE	rs174455	rs174455	61656117	1	1	1	a	g	0.5959	0.6002	0.0616	0.0316	0.05131	++
EIC9	rs143352979:61620628:AAACAAC:A	-	61620628	0.934	1	1	a	aaacaac	0.2703	0.2915	-0.0568	0.0292	0.0514	--
MUFA	rs528285	rs528285	61660704	0.955	1	1	a	g	0.3479	0.3508	-0.0231	0.0119	0.0516	--
OLE	rs174537	rs174537	61552680	1	1	1	t	g	0.194	0.2002	-0.0278	0.0143	0.05166	--
MUFA	rs174537	rs174537	61552680	1	1	1	t	g	0.194	0.2002	-0.0277	0.0142	0.05168	--
STE	11:61604967-C-T	rs2072113	61604967	1	1	1	t	c	0.0757	0.1017	-0.0496	0.0255	0.05199	--
EPA	11:61672645-G-C	rs174475	61672645	1	1	1	c	g	0.1967	0.2125	-0.1101	0.0567	0.05226	+-

DTA	rs174537	rs174537	61552680	1	1	1	t	g	0.194	0.2002	-0.0766	0.0395	0.05232	-+
PLE	rs174458:61656995:C:T	rs174458	61656995	1	1	1	t	c	0.6214	0.6259	0.0615	0.0317	0.05233	++
PLA	rs174459:61657050:C:T	rs174459	61657050	0.998	1	1	t	c	0.6472	0.6527	-0.0778	0.0401	0.05246	--
ARA	11:61620274-T-C	rs72643559	61620274	1	1	1	t	c	0.9312	0.9413	0.0759	0.0392	0.05285	+-
ARA	11:61657110-C-T	rs174460	61657110	1	1	1	t	c	0.6207	0.6262	-0.0384	0.0198	0.0529	--
DGLA	rs174472:61671956:A:G	rs174472	61671956	0.975	1	1	a	g	0.2874	0.2929	0.0531	0.0275	0.05327	++
PLA	rs1151144:61656585:T:C	rs1151144	61656585	0.998	1	1	t	c	0.3474	0.3528	0.0775	0.0401	0.05338	++
PLA	rs174457:61656975:T:C	rs174457	61656975	0.998	1	1	t	c	0.3469	0.3528	0.0774	0.0401	0.0535	++
ARA	11:61603358-A-T	rs2727271	61603358	1	1	1	a	t	0.8983	0.9235	0.0599	0.0311	0.05353	++
PLA	rs1295988:61656658:A:C	rs1295988	61656658	0.998	1	1	a	c	0.3467	0.3528	0.0775	0.0401	0.05353	++
PLA	rs36047269:61656254:T:TC	rs36047269	61656254	0.997	1	1	t	tc	0.3466	0.3528	0.0775	0.0402	0.05356	++
PLA	rs5792240:61656541:A:AT	rs5792240	61656541	0.998	1	1	a	at	0.3467	0.3528	0.0775	0.0401	0.05356	++
DGLA	rs116672159:61648113:C:T	rs116672159	61648113	0.968	1	1	t	c	0.0635	0.0721	-0.1036	0.0537	0.05358	--
LAU	11:61604967-C-T	rs2072113	61604967	1	1	1	t	c	0.0757	0.1017	0.1064	0.0551	0.05358	++
PLA	rs174456:61656182:C:A	rs174456	61656182	0.997	1	1	a	c	0.6472	0.6535	-0.0775	0.0402	0.05359	--
LA	rs174579	rs174579	61605613	0.998	1	1	t	c	0.0912	0.1069	0.115	0.0596	0.05366	++
DPA	rs528285	rs528285	61660704	0.955	1	1	a	g	0.3479	0.3508	0.0715	0.0371	0.05372	++
ARA	rs2727270:61603237:C:T	rs2727270	61603237	0.998	1	1	t	c	0.0765	0.1012	-0.0599	0.0311	0.05377	--
DHA	11:61604967-C-T	rs2072113	61604967	1	1	1	t	c	0.0757	0.1017	-0.077	0.04	0.05427	--
PUFA6	11:61557826-T-C	rs102274	61557826	1	1	1	t	c	0.7982	0.8035	-0.0703	0.0365	0.05441	--
ALA	11:61548559-A-G	rs509360	61548559	1	1	1	a	g	0.7226	0.736	-0.1213	0.0631	0.05452	--
OLE	rs143352979:61620628:AAACAAC:A	-	61620628	0.934	1	1	a	aaacaac	0.2703	0.2915	-0.025	0.013	0.05453	--
ARA	rs174597:61621040:G:C	rs174597	61621040	0.986	1	1	c	g	0.1246	0.128	0.0585	0.0304	0.05458	++
PLA	JHU_11.61657592	rs68112215	61657593	1	1	1	t	c	0.6485	0.6546	-0.077	0.0401	0.05464	--
GLA	rs174468	rs174468	61663691	1	1	1	a	g	0.1639	0.187	0.1232	0.0642	0.05482	++
EPA	rs528285	rs528285	61660704	0.955	1	1	a	g	0.3479	0.3508	0.0925	0.0481	0.05483	-+
BEH	rs174582	rs174582	61607168	1	1	1	a	g	0.8891	0.9021	-0.0692	0.0361	0.05501	--
DHA	rs174564:61588305:A:G	rs174564	61588305	1	1	1	a	g	0.8146	0.8152	0.0594	0.0309	0.05506	++

PUFA6	11:61549458-A-G	rs174534	61549458	1	1	1	a	g	0.813	0.8152	-0.0708	0.0369	0.0552	--
DHA	rs174625:61636013:A:T	rs174625	61636013	0.98	1	1	a	t	0.2476	0.2629	-0.0543	0.0283	0.05541	--
PLE	rs174623:61635294:G:A	rs174623	61635294	0.964	1	1	a	g	0.5268	0.5298	0.0578	0.0302	0.05551	++
PUFA6	rs174579	rs174579	61605613	0.998	1	1	t	c	0.0912	0.1069	0.1024	0.0535	0.05554	++
DHA	rs174455	rs174455	61656117	1	1	1	a	g	0.5959	0.6002	0.0499	0.0261	0.05562	++
LA	rs174559:61581656:G:A	rs174559	61581656	0.999	1	1	a	g	0.1566	0.1611	0.0827	0.0432	0.05573	++
LIG	rs57668028:61591995:GAA:G	-	61591995	0.982	1	1	g	gaa	0.163	0.1686	0.0714	0.0373	0.05579	++
PLA	rs1000778	rs1000778	61655305	1	1	1	a	g	0.3391	0.3483	0.0777	0.0407	0.0559	++
PAL	rs174623:61635294:G:A	rs174623	61635294	0.964	1	1	a	g	0.5268	0.5298	0.0224	0.0117	0.05595	++
ALA	rs174606:61626973:G:T	rs174606	61626973	0.977	1	1	t	g	0.3876	0.3941	0.1053	0.0551	0.05597	++
PLA	rs116139751:61653164:C:T	rs116139751	61653164	0.987	1	1	t	c	0.0597	0.0682	0.1474	0.0772	0.05625	-+
AA	rs174453:61645967:C:G	rs174453	61645967	0.953	1	1	c	g	0.7435	0.759	-0.0439	0.023	0.05626	--
EPA	11:61620274-T-C	rs72643559	61620274	1	1	1	t	c	0.9312	0.9413	-0.1853	0.0971	0.0563	--
ALA	rs74771917:61627960:C:T	rs74771917	61627960	0.99	1	1	t	c	0.097	0.0999	0.1697	0.0889	0.05637	++
DHA	rs174567:61593005:A:G	rs174567	61593005	0.998	1	1	a	g	0.8151	0.8152	0.059	0.0309	0.05645	++
DPA6	rs174587:61612830:C:T	rs174587	61612830	0.961	1	1	t	c	0.0976	0.1113	-0.0898	0.0471	0.05651	--
DHA	rs174458:61656995:C:T	rs174458	61656995	1	1	1	t	c	0.6214	0.6259	0.0494	0.0259	0.05653	++
EIC9	11:61624181-T-C	rs97384	61624181	1	1	1	t	c	0.2561	0.2744	-0.0543	0.0285	0.0566	--
OLE	rs57668028:61591995:GAA:G	-	61591995	0.982	1	1	g	gaa	0.163	0.1686	-0.0286	0.015	0.05668	--
DHA	rs99780	rs99780	61596633	1	1	1	t	c	0.1848	0.1885	-0.0587	0.0308	0.05689	--
PAL	rs174625:61636013:A:T	rs174625	61636013	0.98	1	1	a	t	0.2476	0.2629	-0.0263	0.0138	0.05702	--
STE	rs174473:61672235:T:C	rs174473	61672235	0.984	1	1	t	c	0.4917	0.5	-0.0278	0.0146	0.05722	--
EDA	rs174536	rs174536	61551927	1	1	1	a	c	0.7916	0.7994	-0.0659	0.0347	0.05741	--
NER	rs3168072	rs3168072	61631510	1	1	1	a	t	0.9312	0.9364	-0.1817	0.0957	0.05751	--
ARA	rs526126	rs526126	61624885	1	1	1	c	g	0.8841	0.895	0.0555	0.0293	0.05793	++
EDA	rs528285	rs528285	61660704	0.955	1	1	a	g	0.3479	0.3508	0.0528	0.0279	0.05806	++
PLE	11:61657110-C-T	rs174460	61657110	1	1	1	t	c	0.6207	0.6262	0.0599	0.0317	0.05876	++
DGLA	rs174474:61672265:T:C	rs174474	61672265	0.989	1	1	t	c	0.499	0.5144	0.0457	0.0242	0.05886	++
OLE	rs174583	rs174583	61609750	1	1	1	t	c	0.2006	0.2089	-0.0263	0.0139	0.059	--

DHA	11:61657110-C-T	rs174460	61657110	1	1	1	t	c	0.6207	0.6262	0.0489	0.0259	0.05915	++
EIC9	11:61579463-A-G	rs174554	61579463	1	1	1	a	g	0.8156	0.8156	0.0899	0.0477	0.05924	?+
GLA	rs174579	rs174579	61605613	0.998	1	1	t	c	0.0912	0.1069	0.1752	0.0931	0.05975	++
MUFA	11:61557826-T-C	rs102274	61557826	1	1	1	t	c	0.7982	0.8035	0.0267	0.0142	0.05977	++
EIC9	rs174451:61642763:C:T	rs174451	61642763	0.983	1	1	t	c	0.1266	0.1271	-0.0754	0.0401	0.05987	--
STE	rs76095489:61664711:A:T	rs76095489	61664711	0.907	1	1	a	t	0.9297	0.9365	0.0517	0.0275	0.05989	++
DTA	11:61557826-T-C	rs102274	61557826	1	1	1	t	c	0.7982	0.8035	0.0756	0.0402	0.05993	+-
DTA	rs174565:61591636:C:G	rs174565	61591636	0.997	1	1	c	g	0.8668	0.8706	0.0863	0.0459	0.06018	+-
DTA	rs174536	rs174536	61551927	1	1	1	a	c	0.7916	0.7994	0.0743	0.0396	0.06075	++
LLA	rs174602	rs174602	61624414	1	1	1	t	c	0.7776	0.7822	0.0764	0.0407	0.0608	++
BEH	rs174626	rs174626	61637057	0.982	1	1	a	g	0.5323	0.5363	-0.0418	0.0223	0.06125	--
LAU	rs143352979:61620628:AAACAAC:A	-	61620628	0.934	1	1	a	aaacaac	0.2703	0.2915	0.0707	0.0378	0.06129	++
ARA	11:61604782-A-T	rs2524299	61604782	1	1	1	a	t	0.8992	0.9235	0.0587	0.0314	0.0613	++
DTA	rs174538	rs174538	61560081	0.998	1	1	a	g	0.1683	0.1741	-0.0761	0.0407	0.06156	-+
GLA	rs2727270:61603237:C:T	rs2727270	61603237	0.998	1	1	t	c	0.0765	0.1012	-0.1565	0.0837	0.06164	--
BEH	rs174590:61616977:G:C	rs174590	61616977	0.981	1	1	c	g	0.1133	0.122	0.0641	0.0343	0.06181	++
OLE	11:61557826-T-C	rs102274	61557826	1	1	1	t	c	0.7982	0.8035	0.0266	0.0143	0.06181	++
PLA	rs174464_61657926	rs174464	61657926	0.987	1	1	a	g	0.3487	0.358	0.0756	0.0405	0.06194	++
SFA	rs13966	rs13966	61664992	1	1	1	t	c	0.2541	0.2783	-0.0228	0.0122	0.06205	--
PUFA	11:61549458-A-G	rs174534	61549458	1	1	1	a	g	0.813	0.8152	-0.0683	0.0366	0.06218	--
GLA	11:61603358-A-T	rs2727271	61603358	1	1	1	a	t	0.8983	0.9235	0.156	0.0837	0.06244	++
PLE	rs6591668:61661113:T:C	rs6591668	61661113	0.976	1	1	t	c	0.0547	0.0589	-0.135	0.0726	0.06302	--
LAU	11:61604782-A-T	rs2524299	61604782	1	1	1	a	t	0.8992	0.9235	-0.1023	0.055	0.06312	--
DTA	11:61592362-A-G	rs174566	61592362	1	1	1	a	g	0.7774	0.7868	0.0688	0.037	0.06322	+-
CAP	rs3815045	rs3815045	61669946	1	1	1	a	g	0.2697	0.2727	-0.1047	0.0564	0.06328	--
ALA	rs57668028:61591995:GAA:G	-	61591995	0.982	1	1	g	gaa	0.163	0.1686	0.1315	0.0709	0.06355	++
PUFA3	rs74771917:61627960:C:T	rs74771917	61627960	0.99	1	1	t	c	0.097	0.0999	0.1054	0.0568	0.06362	++
DGLA	rs174473:61672235:T:C	rs174473	61672235	0.984	1	1	t	c	0.4917	0.5	0.0449	0.0242	0.06367	++

PUFA6	rs108499:61547237:C:T	rs108499	61547237	0.985	1	1	t	c	0.1734	0.1744	0.0717	0.0386	0.06369	++
PAL	11:61621194-G-A	rs174598	61621194	1	1	1	a	g	0.2131	0.2167	-0.0268	0.0145	0.06412	--
PUFA6	rs102275	rs102275	61557803	1	1	1	t	c	0.7974	0.803	-0.0673	0.0364	0.06412	--
PUFA	11:61557826-T-C	rs102274	61557826	1	1	1	t	c	0.7982	0.8035	-0.0671	0.0362	0.06421	--
PUFA	rs174597:61621040:G:C	rs174597	61621040	0.986	1	1	c	g	0.1246	0.128	0.0841	0.0454	0.06421	++
BEH	rs422249	rs422249	61639488	0.976	1	1	t	c	0.1873	0.1953	0.0562	0.0303	0.06423	++
DGLA	11:61604782-A-T	rs2524299	61604782	1	1	1	a	t	0.8992	0.9235	0.0724	0.0392	0.0644	++
ALA	rs528285	rs528285	61660704	0.955	1	1	a	g	0.3479	0.3508	0.0991	0.0536	0.06447	++
STE	11:61604782-A-T	rs2524299	61604782	1	1	1	a	t	0.8992	0.9235	0.0471	0.0255	0.06454	++
MUFA	rs102275	rs102275	61557803	1	1	1	t	c	0.7974	0.803	0.0261	0.0141	0.06488	++
PLE	rs7113281:61662780:G:A	rs7113281	61662780	0.984	1	1	a	g	0.94	0.945	0.1327	0.0719	0.06498	++
PAL	rs174472:61671956:A:G	rs174472	61671956	0.975	1	1	a	g	0.2874	0.2929	-0.0246	0.0134	0.06529	--
ARA	rs174587:61612830:C:T	rs174587	61612830	0.961	1	1	t	c	0.0976	0.1113	0.0616	0.0334	0.06537	++
BEH	rs174597:61621040:G:C	rs174597	61621040	0.986	1	1	c	g	0.1246	0.128	0.061	0.0331	0.0654	++
PUFA6	rs174538	rs174538	61560081	0.998	1	1	a	g	0.1683	0.1741	0.0709	0.0385	0.06547	++
BEH	rs6591668:61661113:T:C	rs6591668	61661113	0.976	1	1	t	c	0.0547	0.0589	0.0933	0.0508	0.06597	++
EIC9	11:61621194-G-A	rs174598	61621194	1	1	1	a	g	0.2131	0.2167	-0.058	0.0316	0.06599	--
DHA	rs35473591:61586328:C:CT	rs35473591	61586328	1	1	1	ct	c	0.1844	0.1854	-0.0572	0.0311	0.06604	--
DHA	11:61585144-A-G	rs174562	61585144	1	1	1	a	g	0.8145	0.8156	0.0572	0.0311	0.06608	++
LAU	rs2526680:61611216:C:T	rs2526680	61611216	0.969	1	1	t	c	0.0679	0.0804	0.1078	0.0587	0.06621	++
BEH	rs102275	rs102275	61557803	1	1	1	t	c	0.7974	0.803	-0.0523	0.0285	0.06631	--
LAU	11:61629122-G-A	rs174616	61629122	1	1	1	a	g	0.3808	0.387	0.0696	0.0379	0.06633	++
LAU	rs2072114	rs2072114	61605215	1	1	1	a	g	0.8992	0.9235	-0.1011	0.055	0.06633	--
DGLA	rs174597:61621040:G:C	rs174597	61621040	0.986	1	1	c	g	0.1246	0.128	0.0735	0.04	0.06635	++
EDA	rs7394579:61581450:A:G	rs7394579	61581450	0.984	1	1	a	g	0.8415	0.8467	-0.0694	0.0378	0.0664	--
MUFA	rs57668028:61591995:GAA:G	-	61591995	0.982	1	1	g	gaa	0.163	0.1686	-0.0273	0.0149	0.06644	--
STE	rs174474:61672265:T:C	rs174474	61672265	0.989	1	1	t	c	0.499	0.5144	-0.0266	0.0145	0.06683	--
DGLA	rs174468	rs174468	61663691	1	1	1	a	g	0.1639	0.187	0.0565	0.0308	0.06688	++
CAP	rs174607:61627321:G:C	rs174607	61627321	0.964	1	1	c	g	0.0715	0.0808	-0.184	0.1005	0.06706	+-

DHA	rs2072114	rs2072114	61605215	1	1	1	a	g	0.8992	0.9235	0.0736	0.0402	0.06707	++
LAU	rs174615:61628964:T:A	rs174615	61628964	0.998	1	1	a	t	0.3788	0.386	0.07	0.0383	0.0671	++
DHA	rs5792235:61596322:CA:C	-	61596322	0.998	1	1	ca	c	0.8131	0.8156	0.0569	0.0311	0.06716	++
OLE	rs174538	rs174538	61560081	0.998	1	1	a	g	0.1683	0.1741	-0.0273	0.0149	0.06718	--
LAU	rs174614:61628915:T:C	rs174614	61628915	0.998	1	1	t	c	0.6139	0.6213	-0.0699	0.0383	0.06764	--
OLE	11:61622227-T-C	rs174600	61622227	0.996	1	1	t	c	0.7814	0.7847	0.0247	0.0135	0.06781	++
PUFA3	rs174579	rs174579	61605613	0.998	1	1	t	c	0.0912	0.1069	0.1083	0.0593	0.06786	++
DTA	11:61548559-A-G	rs509360	61548559	1	1	1	a	g	0.7226	0.736	0.064	0.0351	0.06789	++
DPA	rs174597:61621040:G:C	rs174597	61621040	0.986	1	1	c	g	0.1246	0.128	0.0948	0.052	0.06839	++
PAL	rs174626	rs174626	61637057	0.982	1	1	a	g	0.5323	0.5363	0.0209	0.0114	0.06852	++
PUFA6	rs174535	rs174535	61551356	1	1	1	t	c	0.7936	0.803	-0.066	0.0362	0.0686	--
MYR	11:61604967-C-T	rs2072113	61604967	1	1	1	t	c	0.0757	0.1017	0.1013	0.0556	0.06874	++
PLA	rs174450:61641542:G:T	rs174450	61641542	0.959	1	1	t	g	0.5806	0.5931	-0.0744	0.0409	0.06882	--
LA	rs174597:61621040:G:C	rs174597	61621040	0.986	1	1	c	g	0.1246	0.128	0.0924	0.0508	0.06897	+-
SQRAT63	rs174451:61642763:C:T	rs174451	61642763	0.983	1	1	t	c	0.1266	0.1271	0.1068	0.0588	0.06921	++
DHA	rs174553:61575158:A:G	rs174553	61575158	0.999	1	1	a	g	0.8153	0.8156	0.0566	0.0312	0.06929	++
OLE	rs102275	rs102275	61557803	1	1	1	t	c	0.7974	0.803	0.0258	0.0142	0.06943	++
SFA	rs2235093:61665122:A:G	rs2235093	61665122	0.975	1	1	a	g	0.2414	0.2581	-0.023	0.0127	0.06952	--
MUFA	11:61579463-A-G	rs174554	61579463	1	1	1	a	g	0.8156	0.8156	0.0335	0.0184	0.06953	?+
DHA	rs174551:61573684:T:C	rs174551	61573684	1	1	1	t	c	0.8153	0.8156	0.0565	0.0312	0.06966	++
DPA6	rs174474:61672265:T:C	rs174474	61672265	0.989	1	1	t	c	0.499	0.5144	-0.0545	0.03	0.06969	--
DHA	rs174550	rs174550	61571478	1	1	1	t	c	0.8154	0.8156	0.0565	0.0312	0.06983	++
DHA	rs174546	rs174546	61569830	1	1	1	t	c	0.1844	0.1846	-0.0565	0.0312	0.06983	--
DHA	11:61569306-C-G	rs174545	61569306	1	1	1	c	g	0.8154	0.8156	0.0565	0.0312	0.06983	++
DHA	rs174547	rs174547	61570783	1	1	1	t	c	0.8154	0.8156	0.0565	0.0312	0.06983	++
LIG	11:61620274-T-C	rs72643559	61620274	1	1	1	t	c	0.9312	0.9413	0.1184	0.0654	0.06997	++
PLA	rs35867597:61670459:AC:A	-	61670459	0.956	1	1	a	ac	0.0757	0.0829	0.133	0.0734	0.07004	-+
ALA	rs11407273:61627411:C:CT	rs11407273	61627411	0.988	1	1	ct	c	0.6062	0.6108	-0.0992	0.0548	0.07011	--
BEH	rs7113281:61662780:G:A	rs7113281	61662780	0.984	1	1	a	g	0.94	0.945	-0.0907	0.0502	0.07048	--

SQRAT63	11:61647288-G-A	rs7942717	61647288	1	1	1	a	g	0.9008	0.9077	-0.1192	0.066	0.07091	--
DHA	rs1535	rs1535	61597972	1	1	1	a	g	0.811	0.8156	0.056	0.031	0.07093	++
MUFA	rs174618	rs174618	61629322	1	1	1	t	c	0.714	0.72	0.0235	0.013	0.07119	++
MYR	rs4564341:61573540:C:T	rs4564341	61573540	0.935	1	1	t	c	0.0606	0.0673	0.124	0.0688	0.07124	++
PLE	11:61618608-A-G	rs174592	61618608	1	1	1	a	g	0.7478	0.7589	-0.0623	0.0346	0.07135	--
ELA	rs422249	rs422249	61639488	0.976	1	1	t	c	0.1873	0.1953	0.105	0.0583	0.07154	-+
AA	rs174470:61669608:G:A	rs174470	61669608	0.993	1	1	a	g	0.2961	0.3038	0.0371	0.0206	0.07168	-+
LAU	11:61603358-A-T	rs2727271	61603358	1	1	1	a	t	0.8983	0.9235	-0.099	0.055	0.07175	--
LAU	rs2727270:61603237:C:T	rs2727270	61603237	0.998	1	1	t	c	0.0765	0.1012	0.0989	0.055	0.07217	++
PLE	11:61647288-G-A	rs7942717	61647288	1	1	1	a	g	0.9008	0.9077	0.1049	0.0584	0.07226	-+
GLA	11:61604782-A-T	rs2524299	61604782	1	1	1	a	t	0.8992	0.9235	0.149	0.0829	0.07242	++
DTA	rs4246215	rs4246215	61564299	0.995	1	1	t	g	0.1899	0.1976	-0.0713	0.0397	0.07248	-+
LAU	rs174613:61628492:G:A	rs174613	61628492	0.997	1	1	a	g	0.3785	0.3872	0.0686	0.0382	0.07261	++
CAP	rs174469:61667443:C:T	rs174469	61667443	0.987	1	1	t	c	0.2978	0.3066	-0.0976	0.0544	0.07269	--
ELA	rs174455	rs174455	61656117	1	1	1	a	g	0.5959	0.6002	-0.0854	0.0476	0.07276	--
PUFA6	rs174536	rs174536	61551927	1	1	1	a	c	0.7916	0.7994	-0.0644	0.0359	0.07293	--
EPA	rs422249	rs422249	61639488	0.976	1	1	t	c	0.1873	0.1953	-0.1128	0.0629	0.07295	--
SQRAT63	11:61604782-A-T	rs2524299	61604782	1	1	1	a	t	0.8992	0.9235	-0.1014	0.0567	0.07339	--
PUFA	rs108499:61547237:C:T	rs108499	61547237	0.985	1	1	t	c	0.1734	0.1744	0.0684	0.0382	0.07349	++
PLA	rs174466:61659296:A:G	rs174466	61659296	0.966	1	1	a	g	0.359	0.3611	0.072	0.0402	0.07356	++
ALA	11:61549458-A-G	rs174534	61549458	1	1	1	a	g	0.813	0.8152	-0.1222	0.0683	0.07376	--
OLE	11:61621194-G-A	rs174598	61621194	1	1	1	a	g	0.2131	0.2167	-0.0242	0.0136	0.07376	--
ALA	rs108499:61547237:C:T	rs108499	61547237	0.985	1	1	t	c	0.1734	0.1744	0.1259	0.0704	0.07382	++
LA	11:61629122-G-A	rs174616	61629122	1	1	1	a	g	0.3808	0.387	0.0575	0.0322	0.0742	++
MYR	rs2727263:61618012:C:T	rs2727263	61618012	0.975	1	1	t	c	0.086	0.0874	0.0978	0.0548	0.07423	++
PAL	11:61548559-A-G	rs509360	61548559	1	1	1	a	g	0.7226	0.736	0.0245	0.0137	0.07423	++
DPA6	rs174602	rs174602	61624414	1	1	1	t	c	0.7776	0.7822	0.061	0.0343	0.07532	++
PLE	rs174594:61619829:C:A	rs174594	61619829	0.997	1	1	a	c	0.7473	0.7596	-0.0614	0.0346	0.0756	--
DHA	11:61606642-A-G	rs174580	61606642	1	1	1	a	g	0.8005	0.8108	0.0543	0.0306	0.07573	++

PAL	11:61622227-T-C	rs174600	61622227	0.996	1	1	t	c	0.7814	0.7847	0.0257	0.0145	0.07574	++
PLE	rs174449	rs174449	61640379	1	1	1	a	g	0.5785	0.5921	0.0556	0.0313	0.07576	++
LIG	11:61592362-A-G	rs174566	61592362	1	1	1	a	g	0.7774	0.7868	-0.0602	0.0339	0.07585	--
EPA	rs174549	rs174549	61571382	1	1	1	a	g	0.1712	0.173	-0.1133	0.0638	0.07589	--
EPA	rs174555:61579760:T:C	rs174555	61579760	1	1	1	t	c	0.827	0.8288	0.1133	0.0638	0.07589	++
SQRAT63	11:61603358-A-T	rs2727271	61603358	1	1	1	a	t	0.8983	0.9235	-0.1001	0.0564	0.07592	--
EPA	rs174557:61581368:A:G	rs174557	61581368	0.999	1	1	a	g	0.827	0.8285	0.1131	0.0637	0.07593	++
EPA	11:61582708-T-C	rs174561	61582708	1	1	1	t	c	0.827	0.8288	0.1132	0.0638	0.07604	++
SQRAT63	rs2727270:61603237:C:T	rs2727270	61603237	0.998	1	1	t	c	0.0765	0.1012	0.1	0.0564	0.0761	++
ARA	rs174595:61619893:C:T	rs174595	61619893	0.996	1	1	t	c	0.1167	0.1239	0.055	0.031	0.07616	++
GLA	11:61604967-C-T	rs2072113	61604967	1	1	1	t	c	0.0757	0.1017	-0.1469	0.0829	0.07623	--
BEH	rs174595:61619893:C:T	rs174595	61619893	0.996	1	1	t	c	0.1167	0.1239	0.0602	0.0339	0.07629	++
PUFA	rs102275	rs102275	61557803	1	1	1	t	c	0.7974	0.803	-0.0639	0.0361	0.07638	--
LAU	rs2727263:61618012:C:T	rs2727263	61618012	0.975	1	1	t	c	0.086	0.0874	0.0974	0.0551	0.07699	++
PUFA6	rs174597:61621040:G:C	rs174597	61621040	0.986	1	1	c	g	0.1246	0.128	0.0811	0.0459	0.07722	+-
LAU	rs174473:61672235:T:C	rs174473	61672235	0.984	1	1	t	c	0.4917	0.5	0.0614	0.0348	0.07731	++
STE	rs174589:61615803:C:G	rs174589	61615803	0.973	1	1	c	g	0.8774	0.8859	-0.0429	0.0243	0.07746	--
ALA	rs174537	rs174537	61552680	1	1	1	t	g	0.194	0.2002	0.118	0.0669	0.07781	++
DTA	rs102275	rs102275	61557803	1	1	1	t	c	0.7974	0.803	0.0706	0.0401	0.0779	+-
MYR	rs2526680:61611216:C:T	rs2526680	61611216	0.969	1	1	t	c	0.0679	0.0804	0.1059	0.0601	0.07795	++
OLE	rs174565:61591636:C:G	rs174565	61591636	0.997	1	1	c	g	0.8668	0.8706	0.0292	0.0166	0.07795	++
BEH	rs108499:61547237:C:T	rs108499	61547237	0.985	1	1	t	c	0.1734	0.1744	0.0539	0.0306	0.07797	++
PUFA	rs174589:61615803:C:G	rs174589	61615803	0.973	1	1	c	g	0.8774	0.8859	-0.0833	0.0473	0.078	--
LA	rs174589:61615803:C:G	rs174589	61615803	0.973	1	1	c	g	0.8774	0.8859	-0.0933	0.053	0.07814	--
NER	11:61647288-G-A	rs7942717	61647288	1	1	1	a	g	0.9008	0.9077	0.1329	0.0755	0.07837	++
PUFA	rs174538	rs174538	61560081	0.998	1	1	a	g	0.1683	0.1741	0.0669	0.0381	0.0785	++
LAU	rs2851682	rs2851682	61616012	0.983	1	1	a	g	0.9162	0.9209	-0.0968	0.0551	0.07876	--
LA	rs174570	rs174570	61597212	1	1	1	t	c	0.1279	0.1324	0.0799	0.0455	0.07909	++
ALA	rs174595:61619893:C:T	rs174595	61619893	0.996	1	1	t	c	0.1167	0.1239	0.1513	0.0862	0.0792	+-

STE	rs2524296:61601378:C:T	rs2524296	61601378	0.997	1	1	t	c	0.0634	0.0773	-0.0466	0.0266	0.07947	--
MYR	rs143352979:61620628:AAACA AC:A	-	61620628	0.934	1	1	a	aaac aac	0.2703	0.2915	0.065	0.037	0.07951	++
DPA6	rs174473:61672235:T:C	rs174473	61672235	0.984	1	1	t	c	0.4917	0.5	-0.0534	0.0304	0.07952	--
DHA	rs1000778	rs1000778	61655305	1	1	1	a	g	0.3391	0.3483	-0.047	0.0268	0.07954	--
LIG	11:61579463-A-G	rs174554	61579463	1	1	1	a	g	0.8156	0.8156	-0.0972	0.0555	0.07977	?-
ALA	rs174533:61549025:G:A	rs174533	61549025	0.986	1	1	a	g	0.1988	0.2053	0.1161	0.0664	0.08011	++
CAP	rs74771917:61627960:C:T	rs74771917	61627960	0.99	1	1	t	c	0.097	0.0999	0.1343	0.0768	0.08026	++
EPA	rs76095489:61664711:A:T	rs76095489	61664711	0.907	1	1	a	t	0.9297	0.9365	-0.1697	0.097	0.08039	--
MUFA	rs174533:61549025:G:A	rs174533	61549025	0.986	1	1	a	g	0.1988	0.2053	-0.0245	0.014	0.08065	--
AA	rs174469:61667443:C:T	rs174469	61667443	0.987	1	1	t	c	0.2978	0.3066	0.0361	0.0207	0.0807	-+
PLE	rs116139751:61653164:C:T	rs116139751	61653164	0.987	1	1	t	c	0.0597	0.0682	-0.1218	0.0697	0.0807	+-
MUFA	rs174538	rs174538	61560081	0.998	1	1	a	g	0.1683	0.1741	-0.0258	0.0148	0.08085	--
PLE	rs35867597:61670459:AC:A	-	61670459	0.956	1	1	a	ac	0.0757	0.0829	-0.1065	0.061	0.08088	--
LA	rs7394579:61581450:A:G	rs7394579	61581450	0.984	1	1	a	g	0.8415	0.8467	-0.0763	0.0438	0.08096	--
DHA	rs174576	rs174576	61603510	1	1	1	a	c	0.1866	0.1976	-0.0539	0.0309	0.0811	--
MYR	11:61604782-A-T	rs2524299	61604782	1	1	1	a	t	0.8992	0.9235	-0.0967	0.0555	0.08113	--
BEH	11:61557826-T-C	rs102274	61557826	1	1	1	t	c	0.7982	0.8035	-0.0502	0.0288	0.08114	--
PUFA	rs174535	rs174535	61551356	1	1	1	t	c	0.7936	0.803	-0.0626	0.0359	0.08124	--
AA	rs1151139:61671156:C:T	rs1151139	61671156	0.986	1	1	t	c	0.2938	0.3011	0.0358	0.0206	0.08194	-+
LIG	11:61549458-A-G	rs174534	61549458	1	1	1	a	g	0.813	0.8152	-0.0619	0.0356	0.0822	--
BEH	rs57668028:61591995:GAA:G	-	61591995	0.982	1	1	g	gaa	0.163	0.1686	0.054	0.0311	0.08222	++
SQRAT63	rs174449	rs174449	61640379	1	1	1	a	g	0.5785	0.5921	-0.0646	0.0372	0.0826	--
DTA	rs108499:61547237:C:T	rs108499	61547237	0.985	1	1	t	c	0.1734	0.1744	-0.0712	0.0411	0.08278	-+
LAU	11:61627811-T-C	rs174609	61627811	1	1	1	t	c	0.6119	0.6219	-0.0661	0.0381	0.08291	--
STE	rs2845574:61601872:C:T	rs2845574	61601872	0.997	1	1	t	c	0.0634	0.0774	-0.046	0.0265	0.08291	--
PLE	rs174464_61657926	rs174464	61657926	0.987	1	1	a	g	0.3487	0.358	-0.0561	0.0324	0.08292	--
LAU	rs1151139:61671156:C:T	rs1151139	61671156	0.986	1	1	t	c	0.2938	0.3011	-0.0653	0.0377	0.08299	--
BEH	rs35867597:61670459:AC:A	-	61670459	0.956	1	1	a	ac	0.0757	0.0829	0.0735	0.0424	0.08303	++

PUFA3	rs174595:61619893:C:T	rs174595	61619893	0.996	1	1	t	c	0.1167	0.1239	0.0911	0.0526	0.0831	++
STE	rs2845573:61601908:A:G	rs2845573	61601908	0.997	1	1	a	g	0.9226	0.9366	0.046	0.0265	0.08318	++
OLE	rs174550	rs174550	61571478	1	1	1	t	c	0.8154	0.8156	0.0251	0.0145	0.08319	++
OLE	rs174546	rs174546	61569830	1	1	1	t	c	0.1844	0.1846	-0.0251	0.0145	0.08319	--
OLE	11:61569306-C-G	rs174545	61569306	1	1	1	c	g	0.8154	0.8156	0.0251	0.0145	0.08319	++
OLE	rs174547	rs174547	61570783	1	1	1	t	c	0.8154	0.8156	0.0251	0.0145	0.08319	++
LAU	rs2845572:61614595:G:A	rs2845572	61614595	0.98	1	1	a	g	0.0783	0.084	0.0958	0.0553	0.0833	++
EPA	rs174544:61567753:C:A	rs174544	61567753	0.997	1	1	a	c	0.1712	0.1719	-0.111	0.0641	0.08331	--
OLE	rs174551:61573684:T:C	rs174551	61573684	1	1	1	t	c	0.8153	0.8156	0.025	0.0145	0.08377	++
DHA	11:61593816-C-T	rs174568	61593816	1	1	1	t	c	0.1826	0.1827	-0.0545	0.0315	0.08384	--
DHA	rs174559:61581656:G:A	rs174559	61581656	0.999	1	1	a	g	0.1566	0.1611	-0.0564	0.0326	0.08394	--
OLE	rs174570	rs174570	61597212	1	1	1	t	c	0.1279	0.1324	-0.0291	0.0168	0.08401	--
EPA	rs28456:61589481:A:G	rs28456	61589481	1	1	1	a	g	0.827	0.8278	0.11	0.0637	0.08408	++
EPA	11:61581764-T-C	rs174560	61581764	1	1	1	t	c	0.827	0.8278	0.11	0.0637	0.08409	++
EPA	rs174548	rs174548	61571348	1	1	1	c	g	0.827	0.8278	0.11	0.0637	0.08409	++
SQRAT63	11:61604967-C-T	rs2072113	61604967	1	1	1	t	c	0.0757	0.1017	0.0976	0.0565	0.08417	++
DTA	rs174533:61549025:G:A	rs174533	61549025	0.986	1	1	a	g	0.1988	0.2053	-0.0693	0.0402	0.08425	-+
OLE	rs174553:61575158:A:G	rs174553	61575158	0.999	1	1	a	g	0.8153	0.8156	0.025	0.0145	0.08425	++
EPA	11:61580635-C-T	rs174556	61580635	1	1	1	t	c	0.1697	0.1711	-0.1116	0.0646	0.08428	--
PUFA6	11:61629122-G-A	rs174616	61629122	1	1	1	a	g	0.3808	0.387	0.0499	0.0289	0.08437	++
ALA	rs174587:61612830:C:T	rs174587	61612830	0.961	1	1	t	c	0.0976	0.1113	0.1592	0.0924	0.08477	++
PUFA	rs174536	rs174536	61551927	1	1	1	a	c	0.7916	0.7994	-0.0613	0.0356	0.08532	--
LA	rs174565:61591636:C:G	rs174565	61591636	0.997	1	1	c	g	0.8668	0.8706	-0.0775	0.0451	0.08574	--
GLA	rs174597:61621040:G:C	rs174597	61621040	0.986	1	1	c	g	0.1246	0.128	0.1359	0.0792	0.08591	++
OLE	rs3834458:61594920:CT:C	rs774882452	61594920	0.996	1	1	ct	c	0.8157	0.8165	0.025	0.0145	0.08609	++
EPA	rs174559:61581656:G:A	rs174559	61581656	0.999	1	1	a	g	0.1566	0.1611	-0.1114	0.0649	0.08614	--
EPA	rs7394579:61581450:A:G	rs7394579	61581450	0.984	1	1	a	g	0.8415	0.8467	0.1129	0.0659	0.08651	++
LIG	rs4246215	rs4246215	61564299	0.995	1	1	t	g	0.1899	0.1976	0.0618	0.036	0.08654	++
EIC9	rs3168072	rs3168072	61631510	1	1	1	a	t	0.9312	0.9364	-0.0967	0.0564	0.08658	-+

PUFA6	rs174559:61581656:G:A	rs174559	61581656	0.999	1	1	a	g	0.1566	0.1611	0.0669	0.039	0.08659	++
OLE	rs174533:61549025:G:A	rs174533	61549025	0.986	1	1	a	g	0.1988	0.2053	-0.0242	0.0141	0.08663	--
STE	rs138766446:61602458:CCCA:C	rs765564188	61602458	0.992	1	1	ccca	c	0.9218	0.9365	0.0454	0.0265	0.08678	++
CAP	rs174604:61626270:C:G	rs174604	61626270	0.954	1	1	c	g	0.6202	0.6275	-0.0884	0.0516	0.08687	--
LA	rs3834458:61594920:CT:C	rs774882452	61594920	0.996	1	1	ct	c	0.8157	0.8165	-0.0702	0.0411	0.08712	--
OLE	11:61606683-G-A	rs174581	61606683	1	1	1	a	g	0.1866	0.1948	-0.0246	0.0144	0.08717	--
DTA	rs2727265:61609004:C:A	rs2727265	61609004	0.989	1	1	a	c	0.0635	0.0764	-0.0965	0.0565	0.08724	--
AA	11:61620274-T-C	rs72643559	61620274	1	1	1	t	c	0.9312	0.9413	-0.0572	0.0334	0.08742	--
LA	11:61593816-C-T	rs174568	61593816	1	1	1	t	c	0.1826	0.1827	0.0706	0.0413	0.08759	++
STE	rs2727270:61603237:C:T	rs2727270	61603237	0.998	1	1	t	c	0.0765	0.1012	-0.0437	0.0256	0.08794	--
STE	11:61603358-A-T	rs2727271	61603358	1	1	1	a	t	0.8983	0.9235	0.0437	0.0256	0.08798	++
MUFA	rs174535	rs174535	61551356	1	1	1	t	c	0.7936	0.803	0.0242	0.0142	0.08805	++
AA	rs3815045	rs3815045	61669946	1	1	1	a	g	0.2697	0.2727	0.0361	0.0212	0.08837	-+
DHA	rs35867597:61670459:AC:A	-	61670459	0.956	1	1	a	ac	0.0757	0.0829	-0.0829	0.0487	0.08859	--
PLA	rs13966	rs13966	61664992	1	1	1	t	c	0.2541	0.2783	0.0734	0.0431	0.08859	++
ARA	rs93923:61613745:C:T	rs93923	61613745	0.961	1	1	t	c	0.0971	0.1119	0.0573	0.0337	0.08885	++
STE	rs174590:61616977:G:C	rs174590	61616977	0.981	1	1	c	g	0.1133	0.122	0.0416	0.0245	0.08909	++
PUFA	11:61629122-G-A	rs174616	61629122	1	1	1	a	g	0.3808	0.387	0.0488	0.0287	0.08944	++
PAL	rs174474:61672265:T:C	rs174474	61672265	0.989	1	1	t	c	0.499	0.5144	-0.0195	0.0115	0.08972	--
LAU	rs174604:61626270:C:G	rs174604	61626270	0.954	1	1	c	g	0.6202	0.6275	-0.0668	0.0394	0.09003	--
MYR	rs2072114	rs2072114	61605215	1	1	1	a	g	0.8992	0.9235	-0.094	0.0555	0.09009	--
OLE	11:61606642-A-G	rs174580	61606642	1	1	1	a	g	0.8005	0.8108	0.0243	0.0144	0.09011	++
PLE	rs1000778	rs1000778	61655305	1	1	1	a	g	0.3391	0.3483	-0.0552	0.0326	0.09041	--
OLE	rs35473591:61586328:C:CT	rs35473591	61586328	1	1	1	ct	c	0.1844	0.1854	-0.0245	0.0145	0.09079	--
AA	rs528285	rs528285	61660704	0.955	1	1	a	g	0.3479	0.3508	-0.0327	0.0194	0.0908	--
GLA	rs2072114	rs2072114	61605215	1	1	1	a	g	0.8992	0.9235	0.1401	0.0828	0.09084	++
MUFA	rs174620:61629747:A:G	rs174620	61629747	0.996	1	1	a	g	0.7131	0.7207	0.0222	0.0131	0.09089	++
OLE	11:61585144-A-G	rs174562	61585144	1	1	1	a	g	0.8145	0.8156	0.0245	0.0145	0.09109	++

DHA	rs2524296:61601378:C:T	rs2524296	61601378	0.997	1	1	t	c	0.0634	0.0773	-0.0748	0.0443	0.0913	--
DHA	rs174451:61642763:C:T	rs174451	61642763	0.983	1	1	t	c	0.1266	0.1271	-0.0661	0.0391	0.09131	--
OLE	rs174576	rs174576	61603510	1	1	1	a	c	0.1866	0.1976	-0.0245	0.0145	0.09147	--
DTA	rs2845573:61601908:A:G	rs2845573	61601908	0.997	1	1	a	g	0.9226	0.9366	0.0959	0.0568	0.0915	++
DTA	rs2845574:61601872:C:T	rs2845574	61601872	0.997	1	1	t	c	0.0634	0.0774	-0.0959	0.0568	0.09153	--
LIG	rs7394579:61581450:A:G	rs7394579	61581450	0.984	1	1	a	g	0.8415	0.8467	-0.0643	0.0381	0.09169	--
LA	rs174590:61616977:G:C	rs174590	61616977	0.981	1	1	c	g	0.1133	0.122	0.0887	0.0526	0.09179	+-
LIG	rs174538	rs174538	61560081	0.998	1	1	a	g	0.1683	0.1741	0.0615	0.0365	0.09179	++
DTA	rs138766446:61602458:CCCA:C	rs765564188	61602458	0.992	1	1	cc ca	c	0.9218	0.9365	0.0956	0.0567	0.09186	++
ALA	rs93923:61613745:C:T	rs93923	61613745	0.961	1	1	t	c	0.0971	0.1119	0.1555	0.0923	0.09187	++
DHA	rs2845574:61601872:C:T	rs2845574	61601872	0.997	1	1	t	c	0.0634	0.0774	-0.0745	0.0442	0.09208	--
EDA	rs138766446:61602458:CCCA:C	rs765564188	61602458	0.992	1	1	cc ca	c	0.9218	0.9365	-0.0841	0.0499	0.09212	--
DHA	rs2845573:61601908:A:G	rs2845573	61601908	0.997	1	1	a	g	0.9226	0.9366	0.0745	0.0442	0.09215	++
DHA	11:61605499-T-A	rs174578	61605499	1	1	1	a	t	0.1844	0.192	-0.0522	0.031	0.09233	--
DHA	rs174577	rs174577	61604814	1	1	1	a	c	0.1844	0.192	-0.0522	0.031	0.09233	--
DTA	rs2524296:61601378:C:T	rs2524296	61601378	0.997	1	1	t	c	0.0634	0.0773	-0.0957	0.0569	0.09234	--
STE	rs174462:61657666:G:A	rs174462	61657666	0.979	1	1	a	g	0.2403	0.2451	0.0304	0.0181	0.09242	++
ELA	rs174464_61657926	rs174464	61657926	0.987	1	1	a	g	0.3487	0.358	0.0816	0.0485	0.09248	++
EDA	rs2845573:61601908:A:G	rs2845573	61601908	0.997	1	1	a	g	0.9226	0.9366	-0.0841	0.05	0.0927	--
EDA	rs2845574:61601872:C:T	rs2845574	61601872	0.997	1	1	t	c	0.0634	0.0774	0.0841	0.05	0.09273	++
DHA	rs3834458:61594920:CT:C	rs774882452	61594920	0.996	1	1	ct	c	0.8157	0.8165	0.0527	0.0314	0.09283	++
EIC9	rs174450:61641542:G:T	rs174450	61641542	0.959	1	1	t	g	0.5806	0.5931	0.0499	0.0297	0.09296	++
PUFA	rs174590:61616977:G:C	rs174590	61616977	0.981	1	1	c	g	0.1133	0.122	0.0788	0.0469	0.09302	++
LIG	rs108499:61547237:C:T	rs108499	61547237	0.985	1	1	t	c	0.1734	0.1744	0.0619	0.0368	0.09304	++
OLE	rs2845573:61601908:A:G	rs2845573	61601908	0.997	1	1	a	g	0.9226	0.9366	0.0347	0.0206	0.09307	++
OLE	rs174535	rs174535	61551356	1	1	1	t	c	0.7936	0.803	0.0239	0.0142	0.09311	++
EDA	rs2524296:61601378:C:T	rs2524296	61601378	0.997	1	1	t	c	0.0634	0.0773	0.0841	0.0501	0.09316	++
AA	rs76095489:61664711:A:T	rs76095489	61664711	0.907	1	1	a	t	0.9297	0.9365	-0.0605	0.036	0.09318	--

OLE	rs2845574:61601872:C:T	rs2845574	61601872	0.997	1	1	t	c	0.0634	0.0774	-0.0347	0.0206	0.09323	--
DGLA	rs174595:61619893:C:T	rs174595	61619893	0.996	1	1	t	c	0.1167	0.1239	0.0701	0.0418	0.09336	++
EIC9	rs174618	rs174618	61629322	1	1	1	t	c	0.714	0.72	0.0487	0.029	0.09336	++
ARA	rs174635:61647427:T:G	rs174635	61647427	0.969	1	1	t	g	0.355	0.3658	0.0348	0.0208	0.09393	++
MUFA	rs2845573:61601908:A:G	rs2845573	61601908	0.997	1	1	a	g	0.9226	0.9366	0.0344	0.0206	0.09423	++
MUFA	rs2845574:61601872:C:T	rs2845574	61601872	0.997	1	1	t	c	0.0634	0.0774	-0.0344	0.0206	0.0944	--
EPA	rs2727270:61603237:C:T	rs2727270	61603237	0.998	1	1	t	c	0.0765	0.1012	-0.1278	0.0764	0.09444	--
OLE	rs174567:61593005:A:G	rs174567	61593005	0.998	1	1	a	g	0.8151	0.8152	0.0242	0.0144	0.0945	++
PAL	rs174537	rs174537	61552680	1	1	1	t	g	0.194	0.2002	-0.0252	0.0151	0.09468	--
ALA	rs174538	rs174538	61560081	0.998	1	1	a	g	0.1683	0.1741	0.1164	0.0697	0.09472	++
EPA	11:61603358-A-T	rs2727271	61603358	1	1	1	a	t	0.8983	0.9235	0.1276	0.0764	0.09476	++
PUFA6	rs174589:61615803:C:G	rs174589	61615803	0.973	1	1	c	g	0.8774	0.8859	-0.0798	0.0478	0.09485	--
PLA	11:61647288-G-A	rs7942717	61647288	1	1	1	a	g	0.9008	0.9077	-0.1086	0.065	0.095	+-
LIG	rs174559:61581656:G:A	rs174559	61581656	0.999	1	1	a	g	0.1566	0.1611	0.0625	0.0374	0.0952	++
OLE	rs2524296:61601378:C:T	rs2524296	61601378	0.997	1	1	t	c	0.0634	0.0773	-0.0345	0.0207	0.09534	--
PLE	rs174601	rs174601	61623140	1	1	1	t	c	0.2455	0.265	0.0565	0.0339	0.09536	++
OLE	11:61593816-C-T	rs174568	61593816	1	1	1	t	c	0.1826	0.1827	-0.0245	0.0147	0.0954	--
ARA	rs174634:61647387:G:C	rs174634	61647387	0.97	1	1	c	g	0.6341	0.6449	-0.0346	0.0208	0.09565	--
PLE	rs143352979:61620628:AAACAAC:A	-	61620628	0.934	1	1	a	aaacaac	0.2703	0.2915	0.0573	0.0344	0.09609	-+
DHA	rs138766446:61602458:CCCA:C	rs765564188	61602458	0.992	1	1	ccca	c	0.9218	0.9365	0.0734	0.0441	0.09618	++
DGLA	rs4564341:61573540:C:T	rs4564341	61573540	0.935	1	1	t	c	0.0606	0.0673	-0.0772	0.0464	0.09622	--
PAL	rs174533:61549025:G:A	rs174533	61549025	0.986	1	1	a	g	0.1988	0.2053	-0.0246	0.0148	0.09641	--
PAL	rs1151139:61671156:C:T	rs1151139	61671156	0.986	1	1	t	c	0.2938	0.3011	0.021	0.0126	0.09661	++
MUFA	rs2524296:61601378:C:T	rs2524296	61601378	0.997	1	1	t	c	0.0634	0.0773	-0.0342	0.0206	0.0967	--
LIG	rs72643557:61579427:C:T	rs72643557	61579427	0.936	1	1	t	c	0.0607	0.0734	-0.107	0.0644	0.09671	--
STE	rs174454:61650747:G:A	rs174454	61650747	0.963	1	1	a	g	0.6633	0.6834	-0.0263	0.0158	0.09738	--
MYR	rs2727270:61603237:C:T	rs2727270	61603237	0.998	1	1	t	c	0.0765	0.1012	0.0917	0.0553	0.09753	++
DTA	rs174535	rs174535	61551356	1	1	1	t	c	0.7936	0.803	0.0665	0.0402	0.09763	+-

ARA	rs174455	rs174455	61656117	1	1	1	a	g	0.5959	0.6002	-0.0327	0.0197	0.09776	--
MUFA	11:61622227-T-C	rs174600	61622227	0.996	1	1	t	c	0.7814	0.7847	0.0223	0.0135	0.09776	++
MYR	11:61603358-A-T	rs2727271	61603358	1	1	1	a	t	0.8983	0.9235	-0.0916	0.0553	0.09795	--
LLA	rs174618	rs174618	61629322	1	1	1	t	c	0.714	0.72	-0.0686	0.0415	0.0981	--
DHA	rs7394579:61581450:A:G	rs7394579	61581450	0.984	1	1	a	g	0.8415	0.8467	0.0547	0.0331	0.09815	++
STE	rs4564341:61573540:C:T	rs4564341	61573540	0.935	1	1	t	c	0.0606	0.0673	-0.0478	0.0289	0.09817	--
OLE	rs174564:61588305:A:G	rs174564	61588305	1	1	1	a	g	0.8146	0.8152	0.0239	0.0144	0.09822	++
DPA6	rs174579	rs174579	61605613	0.998	1	1	t	c	0.0912	0.1069	-0.0799	0.0484	0.09854	--
LIG	rs174564:61588305:A:G	rs174564	61588305	1	1	1	a	g	0.8146	0.8152	-0.0588	0.0356	0.0986	--
OLE	rs174601	rs174601	61623140	1	1	1	t	c	0.2455	0.265	-0.0212	0.0128	0.0988	--
EIC9	rs174458:61656995:C:T	rs174458	61656995	1	1	1	t	c	0.6214	0.6259	0.0482	0.0292	0.09913	++
DPA	11:61647288-G-A	rs7942717	61647288	1	1	1	a	g	0.9008	0.9077	0.1064	0.0646	0.09933	++
MUFA	rs174582	rs174582	61607168	1	1	1	a	g	0.8891	0.9021	0.0343	0.0208	0.09942	++
PLE	rs174463:61657838:G:A	rs174463	61657838	0.988	1	1	a	g	0.6465	0.6513	0.0531	0.0323	0.09943	++

All SNP x FA combinations in the FADS1/2/3 region are listed where p<0.1

All fatty acids were log(FA) phenotypes *<u>except</u>* sqroot(PUFA6/PUFA3) - SQRAT63

NB: Effect size (beta) and standard errors have NOT been transformed from the log() or sqroot() transformed phenotypes

Columns:

FA	Fatty Acid Phenotype
MarkerName	Unique marker name as tested (for consistency between two studies)
rs#	dbSNP rs# If available
pos	position
minInfo	Minimum IMPUTE info between the two studies

InProv	SNP in PROVIDE set (passed QC) 1/0
InCryp	SNP in Crypto (passed QC) 1/0
A1	Ref Allele
A2	Alt Allele
MinFreq	Minimum frequency of A1
MaxFreq	Maximum frequency of A1
Effect	untransformed beta for A1
StdErr	untransformed beta standard error
P-value	P-value for SNP
Dir	direction of effect for two studies
