# Supplementary material for: Multiplex genomewide association analysis of breast milk fatty acid composition extends the phenotypic association and potential selection of FADS1 variants to arachidonic acid, a critical infant micronutrient
Source: J Med Genet. 2018 Mar 7;55(7):459–68. doi: 10.1136/jmedgenet-2017-105134 (PMC6047159; doi:10.1136/jmedgenet-2017-105134)
Supplement: Supplementary file 9 [file jmedgenet-2017-105134supp009.pdf]

Supplementary Table S6.

Affymetrix MalChip results for common SNPs (MAF > 0.05) associated with FA concentration in breast milk at a significance level of $1x10^{-6}$, ranked by maternal p-value, for tests of association in any of 26 fatty acids and 7 derived FA fractions (PROVIDE Study only).

							Maternal				Infant			
Gene	**FA**	**SNP**	**Chr**	**Position (bp)**	**Minor**	**Major**	**N**	**β[a]**	**SE β**	**P-value**	**MAF**	**β[a]**	**P-value**	**P-value (HW)[b]**
FADS1	AA	rs174546	11	61569830	T	C	616	1.29	1.04	$2.1x10^{-11}$	0.19	1.13	$5.8x10^{-11}$	0.90
FADS1	AA	rs174550	11	61571478	C	T	616	1.29	1.04	$2.1x10^{-11}$	0.19	1.13	$5.8x10^{-11}$	0.90
FADS1	AA	rs174547	11	61570783	C	T	614	1.29	1.04	$2.6x10^{-11}$	0.19	1.13	$7.5x10^{-11}$	0.90
FADS2	AA	rs99780	11	61596633	T	C	616	1.28	1.04	$6.1x10^{-11}$	0.19	1.12	$1.0x10^{-10}$	1
FADS2	AA	rs1535	11	61597972	G	A	616	1.28	1.04	$6.9x10^{-11}$	0.19	1.12	$9.5x10^{-11}$	0.90
FADS1	AA	rs174537	11	61552680	T	G	616	1.28	1.04	$6.0x10^{-10}$	0.20	1.11	$5.8x10^{-9}$	0.22
FADS1	AA	rs174548	11	61571348	G	C	615	1.27	1.04	$8.1x10^{-10}$	0.17	1.12	$1.2x10^{-9}$	0.78
TMEM258	AA	rs102275	11	61557803	C	T	616	1.27	1.04	$1.1x10^{-9}$	0.21	1.11	$9.6x10^{-9}$	0.27
FADS2	AA	rs174576	11	61603510	A	C	613	1.25	1.04	$2.1x10^{-9}$	0.20	1.11	$3.1x10^{-9}$	0.37
FEN1	AA	rs4246215	11	61564299	T	G	615	1.24	1.04	$3.1x10^{-9}$	0.20	1.11	$3.3x10^{-9}$	1
FADS2	AA	rs174570	11	61597212	T	C	616	1.30	1.05	$6.5x10^{-9}$	0.13	1.12	$7.3x10^{-8}$	0.49
MYRF	AA	rs174535	11	61551356	C	T	616	1.25	1.04	$9.0x10^{-9}$	0.21	1.10	$7.4x10^{-8}$	0.28
FADS2	AA	rs174583	11	61609750	T	C	615	1.22	1.04	$1.7x10^{-8}$	0.21	1.10	$1.5x10^{-8}$	1
FADS2	AA	rs174572	11	61598288	T	C	616	1.30	1.05	$1.1x10^{-7}$	0.12	1.12	$4.0x10^{-7}$	0.032
FADS2	AA	rs174575	11	61602003	G	C	616	1.30	1.05	$1.1x10^{-7}$	0.12	1.12	$4.0x10^{-7}$	0.032
PLEKHA7	MUFA	rs7933299	11	16926899	T	C	616	1.24	1.04	$1.2x10^{-7}$	0.07	1.06	$4.5x10^{-5}$	1
PLEKHA7	MUFA	rs7944146	11	16927022	C	T	616	1.25	1.05	$4.6x10^{-7}$	0.06	1.07	$1.4x10^{-4}$	0.43

All beta and SE estimated values were transformed from log(FA) phenotype under additive genetic model to multiplicative model for FA as the outcome. MAF, minor allele frequency; N, Number of samples analyzed. Results significant at the experiment-wise error rate as shown in bold.

[a] Association beta values are for the major allele and measure the multiplicative change on %FA per copy of SNP major allele

[b] p-value for Hardy-Weinberg equilibrium
