# Supplementary material for: Multiplex genomewide association analysis of breast milk fatty acid composition extends the phenotypic association and potential selection of FADS1 variants to arachidonic acid, a critical infant micronutrient
Source: J Med Genet. 2018 Mar 7;55(7):459–68. doi: 10.1136/jmedgenet-2017-105134 (PMC6047159; doi:10.1136/jmedgenet-2017-105134)
Supplement: Supplementary file 10 [file jmedgenet-2017-105134supp010.pdf]

Supplementary Table S7.

Replication of the arachidonic acid breast milk association observed in this study from previous published studies.

Reference	Age at BM Sample	N(SNPs) Tested	FAs Studied	N	SNP	SNP Position	r^2 with rs174556	Model/Test Used	β(AA)	SE β	p-value (AA)	Comments
This study	3-43 days	180	26	1,142	rs174556	61,580,635	1.0	Log-additive	-17.6%[a]	1.03	$1.5x10^{-10}$	AA most significant
Xie, 2008	1 month	1	19	54	rs174553	61,575,158	0.91	Non-parametric Kruskal-Wallis	N/A	N/A	0.003	AA most significant omega-6
Moltó-Puigmartí, 2010	1 month	3	14	309	rs3834458	61,594,921	0.92	Additive	-0.07	0.007	$4x10^{-24}$	AA most significant[b]
Lattka, 2011,	1.5 month	8	26	713	rs174547	61,570,783	0.91	Additive	-0.039	0.013	0.003	AA most significant omega-6
Lattka, 2011	6 months	8	26	423	rs174547	61,570,783	0.91	Additive	-0.084	0.023	0.0002	AA most significant omega-6
Morales, 2011	2-4 days (colostrum)	13	5[c]	270	rs174537	61,552,680	0.83	Log-additive	-10%		0.0002	AA most significant
					rs174570	61,575,158	0.59	Log-additive	-13%		0.0003	AA most significant

All SNP positions are on chromosome 11, hg19/build 37. SNPs Tested in the *FADS1/2/3* region.

[a] Transformed to percentage with minor allele (T) as reference from Table 2 for consistency with Morales et al. [26]

[b] Computed from summary data for 14 FAs in published article table.

[c] FAs tested LA, AA, DGLA, DPA (all omega-6) + DHA (omega-3)
