# Supplementary material for: Multiplex genomewide association analysis of breast milk fatty acid composition extends the phenotypic association and potential selection of FADS1 variants to arachidonic acid, a critical infant micronutrient
Source: J Med Genet. 2018 Mar 7;55(7):459–68. doi: 10.1136/jmedgenet-2017-105134 (PMC6047159; doi:10.1136/jmedgenet-2017-105134)
Supplement: Supplementary file 11 [file jmedgenet-2017-105134supp011.pdf]

Supplementary Table S8.

Tissues for which rs174556 is a significant cis-eQTL, version 6 GTEx, ranked by p-value.

Gencode Id	*Gene Symbol*	P-Value	Effect Size	Tissue
ENSG00000134824.9	*FADS2*	6.3×10^{-32}	0.81	Whole Blood
ENSG00000149485.12	*FADS1*	1.2×10^{-18}	-0.79	Pancreas
ENSG00000134824.9	*FADS2*	3.1×10^{-14}	0.57	Esophagus Muscularis
ENSG00000134824.9	*FADS2*	2.5×10^{-13}	0.27	Fibroblasts
ENSG00000134824.9	*FADS2*	3.9×10^{-13}	0.42	Colon Transverse
ENSG00000149485.12	*FADS1*	1.8×10^{-12}	-0.73	Brain Cerebellum
ENSG00000149485.12	*FADS1*	1.0×10^{-11}	-0.42	Nerve Tibial
ENSG00000134824.9	*FADS2*	2.7×10^{-11}	0.43	Thyroid
ENSG00000134824.9	*FADS2*	4.1×10^{-11}	1.1	Spleen
ENSG00000149485.12	*FADS1*	1.2×10^{-10}	-0.43	Esophagus Mucosa
ENSG00000149485.12	*FADS1*	1.6×10^{-9}	-0.4	Stomach
ENSG00000134824.9	*FADS2*	1.7 x 10-9	0.41	Heart LV
ENSG00000149485.12	*FADS1*	5.1 x 10-9	-0.23	Muscle Skeletal
ENSG00000134824.9	*FADS2*	3.1 x 10-8	0.82	Small Intestine
ENSG00000134824.9	*FADS2*	3.7×10^{-8}	0.39	Aorta
ENSG00000134824.9	*FADS2*	1.1×10^{-7}	0.37	Nerve Tibial
ENSG00000134825.9	*TMEM258*	1.8×10^{-7}	0.2	Skin Sun Exposed
ENSG00000134824.9	*FADS2*	2.2×10^{-7}	0.26	Muscle Skeletal
ENSG00000149485.12	*FADS1*	2.4×10^{-7}	-0.57	Brain CerebHemi
ENSG00000134824.9	*FADS2*	5.5×10^{-7}	0.51	Esophagus GJ
ENSG00000134824.9	*FADS2*	1.2×10^{-6}	0.52	Colon Sigmoid
ENSG00000134824.9	*FADS2*	1.6×10^{-6}	0.25	Artery Tibial
ENSG00000149485.12	*FADS1*	1.7×10^{-6}	-0.28	Thyroid
ENSG00000221968.4	*FADS3*	2.4×10^{-6}	-0.44	Brain Cerebellum

ENSG00000124915.6	*RP11-467L20.10*	2.6×10^{-6}	-0.69	Liver
ENSG00000134825.9	*TMEM258*	4.1×10^{-6}	0.16	Artery Tibial
ENSG00000149485.12	*FADS1*	6.1×10^{-6}	-0.33	Testis
ENSG00000134824.9	*FADS2*	7.4×10^{-6}	0.33	Heart Atrial
ENSG00000134825.9	*TMEM258*	9.9×10^{-6}	0.16	Whole Blood
ENSG00000149485.12	*FADS1*	1.2×10^{-5}	-0.25	Heart LV
ENSG00000149485.12	*FADS1*	4.3×10^{-5}	-0.22	Artery Tibial
ENSG00000134825.9	*TMEM258*	4.9×10^{-5}	0.13	Fibroblasts
ENSG00000134824.9	*FADS2*	5.3×10^{-5}	0.22	Lung
ENSG00000134825.9	*TMEM258*	6.4×10^{-5}	0.15	Adipose Sub
ENSG00000134780.5	*DAGLA*	8.4×10^{-5}	-0.14	Fibroblasts
ENSG00000134825.9	*TMEM258*	8.9×10^{-5}	0.17	Nerve Tibial

Tissue abbreviations: Fibroblasts: Cells - Transformed fibroblasts; Esophagus GJ: Esophagus - Gastroesophageal Junction; Heart Atrial: Heart - Atrial Appendage; Heart LV: Heart - Left Ventricle; Brain CerebHemi: Brain - Cerebellar Hemisphere; Skin Sun Exposed: Skin – Sun Exposed (Lower leg); Small Intestine: Small Intestine - Terminal Ileum.
